# Building Resilient and Responsive Health Research Systems:Responses and the Lessons Learned from the COVID-19 Pandemic

**DOI:** 10.1186/s12961-024-01229-0

**Published:** 2025-03-26

**Authors:** Mark Embrett, Andrea Carson, Meaghan Sim, Aislinn Conway, Emily Moore, Kristy Hancock, Iwona Bielska

**Affiliations:** 1 Research, Innovation, and Discovery, Nova Scotia Health, 90 Lovett Lake Ct, Halifax, NS B3S 0H6 Canada; 2 London Health Sciences Centre, 800 Commissioners Rd E, London, ON N6A 5W9 Canada; 3 Department of Health Policy and Management, Jagiellonian University Medical College, 8 Skawińska Street, 31-066 Kraków, Poland; 4 Department of Health Research Methods, Evidence, and Impact, McMaster University, 1280 Main Street West, Hamilton, ON L8S 4L8 Canada; 5 Evidence Synthesis Ireland, Evidence Synthesis Ireland Áras Moyola, University of Galway, University Road, Galway, Ireland; 6 Stanford University School of Medicine, 291 Campus Drive Li Ka Shing Building Stanford, Stanford, CA United States of America; 7 Maritime SPOR SUPPORT Unit, 5790 University Avenue, Halifax, NS B3H 1V7 Canada

**Keywords:** COVID-19, Health research systems, Pandemic response, Interdisciplinary collaboration, Strategic planning

## Abstract

**Background:**

The coronavirus disease 2019 (COVID-19) pandemic highlighted the crucial role of robust health research systems (HRSs) in supporting effective public health responses. Understanding the responses and lessons learned from HRS during the pandemic is vital for future preparedness.

**Methods:**

This environmental scan examined high income Countries with a HRS that responded to the COVID-19 pandemic using both academic and grey literature sources to gather comprehensive insights into these areas. The analysis was structured using an organizing framework to facilitate systematic extraction and synthesis of relevant information. A total of 5336 sources were identified of which 3609 were screened following duplicate removal. A total of 117 full-text sources were reviewed leading to 65 being included.

**Findings:**

Effective interdisciplinary and cross-sector collaborations significantly enhanced the capacity to respond to the pandemic. Clear and streamlined governance structures were essential for coordinated efforts across various entities, facilitating swift decision-making and resource allocation. The robustness of pre-existing research infrastructures played a crucial role in the rapid mobilization of resources and execution of large-scale research projects. Knowledge mobilization efforts were vital in disseminating research findings promptly to inform public health responses. Continuous tracking and evaluation of health research activities enabled real-time adjustments and informed decision-making. Rapid identification and funding of research priorities, including vaccine and therapeutic development, were critical in addressing urgent public health needs. Effective resource allocation and capacity-building efforts ensured focused and accelerated research responses. Comprehensive strategic planning, involving stakeholder engagement and robust monitoring tools, was essential for aligning research efforts with health system needs.

**Conclusion:**

The findings underscore the necessity of flexible funding mechanisms, enhanced data-sharing practices and robust strategic planning to prepare for future health emergencies. Policy implications emphasize the need for sustained investments in health policy and systems research (HPSR) and the development of comprehensive governance frameworks. Research implications highlight the importance of community engagement and interdisciplinary partnerships. For decision-makers, the study stresses the importance of rapid response mechanisms and evidence-based policy making. Health research systems must prioritize maintaining adaptable infrastructures and strategic planning to ensure effective crisis response. Despite potential biases and the rapidly evolving context, this comprehensive analysis provides valuable lessons for strengthening HRSs to address future public health challenges.

**Supplementary Information:**

The online version contains supplementary material available at 10.1186/s12961-024-01229-0.

## Background

The coronavirus disease 2019 (COVID-19) pandemic highlighted the value of health research systems (HRSs) in supporting the pandemic response and the relational role that a well-functioning HRS can play in optimizing health system performance. HRSs across the globe have participated in endeavours focussed on reducing the impacts of COVID-19 on society – from surveillance to accelerating the application of rapid research methods for evidence products leading to timely evidence needed for health systems planning and decision-making to ensure funding for the discovery of COVID-19 vaccines and therapeutics. Collectively, the activities of the research ecosystem played a central role in determining the success (or lack thereof) of the response.

In Canada, the Canadian Institutes of Health Research (CIHR) serves as the principal health research investment agency of the Government of Canada [1]. In 2022, the CIHR established the Centre for Research on Pandemic Preparedness and Health Emergencies (CRPPHE) with the goal of “Protect[ing] the health of all Canadians by developing and mobilizing health research for pandemic and health emergency preparedness, prevention, response and recovery that contributes meaningfully to timely, equitable and effective responses and recovery” [2]. To contribute to these goals and provide lessons learned to the CRPPHE and CIHR that may be used to better inform a Canadian response to the next public health emergency, we conducted an environmental scan in search of “system-level perspectives” of HRS pandemic responses in high-income countries with health research funding organizations similar to CIHR.

Our objectives were to provide CRPPHE with evidence-based report that (1) described the HRS responses, lessons learned and emerging recommendations related to the COVID-19 pandemic in Canada and other high income countries, and (2) identified the underlying mechanisms and principles of the organizational, programmatic and governance structures that were required to support an effective research response to the pandemic, such as for research prioritization, coordination, sharing, or mobilization.

## Methods

The approach to this environmental scan was informed by the steps outlined by Tricco and colleagues [3, 4] and Strauss and Peters, Godfrey and colleagues [5]. The steps included protocol development, literature search screening, data extraction and knowledge synthesis. The search strategy was reported according to the Preferred Reporting Items for Systematic Reviews and Meta-Analyses (PRISMA) Statement for Reporting Literature Searches in Systematic Reviews [3]. It is noteworthy that to inform Centre for Research on Pandemic Preparedness and Health Emergencies (CRPHHE) in a timely fashion, this study was conducted within a time frame of 4 months (November 2023 to February 2024); therefore, certain trade-offs such as single screening and extraction were made. Only high-income countries were included as they would have most relevant lessons learned for Canada.

### Search strategy

Published and unpublished (grey) literature was eligible for inclusion in this review. To identify published literature, a library scientist developed and executed database searches on 22 November 2023, in MEDLINE (Ovid) and Embase (Elsevier). The database searches were developed using the following key concepts: COVID-19, research response and lessons learned. A modified version of the CADTH (Canadian Agency for Drugs and Technologies in Health) COVID-19 search filter for MEDLINE was used for the COVID-19 concept and was adapted to Embase. Publications reporting on clinical trials were removed from the results in both databases using a modified version of the CADTH clinical trial search filter for MEDLINE, which was adapted to Embase. No geographic or language limits were applied to the searches. Appendix E lists the Medline and Embase searches.

The grey literature search was prioritized based on targeted countries expected to contain the most relevant lessons learned for Canada. These countries (Canada, United States, Australia, New Zealand) were selected on the basis of initial searches and consultations between authors and members of CRPPHE. Authors conducted website searches for provincial governments and their health authorities, major universities and health research organizations. Examples of organizations searched included CIHR, Natural Sciences and Engineering Research Council of Canada (NSERC), Social Sciences and Humanities Research Council (SSHRC), Health Canada, Research Nova Scotia, Nova Scotia Health, US Centers for Disease Control and Prevention (CDC; United States), National Institutes of Health (NIH; United States), National Institute for Health Research (NIHR; United Kingdom), Medical Research Council (MRC; United Kingdom) and the National Health and Medical Research Council (Australia). We searched for reports or briefs on the impact or assessment of the health research system supporting the research activities. Additionally, advanced Google search strings were used to identify relevant grey literature not captured in the website searches. Search terms included “health research system”, “COVID-19”, “lessons learned” and “evaluation”. The full Google search strings are included in Appendix E. We completed reference chaining on all academic and grey literature.

### Screening

The research team conducted single screening of titles and abstracts followed by single reviewer full-text screening based on eligibility criteria. Each author, except for the scientific librarian (K.H.), were involved. Eligibility criteria (detailed in Appendix E) were discussed among authors and in collaboration with CRPHHE to ensure the most relevant sources were included. All eligible sources were extracted for key data points. *Covidence* software was used to remove duplicate database records, organize the screening and facilitate extraction. Key data points included: study characteristics (country, design, methods, aim), responses (including categories), evaluative outcomes of responses, lessons learned (including categories) and recommendations (including categories). Responses, lessons learned and recommendations were extracted into descriptive paragraphs by the authors.

### Analysis

We used an organizing framework for our analysis. The use of an organizing framework for reviews facilitates the extraction and analysis of information while also enhancing dissemination to an audience of policymakers and health systems managers [6]. The HRS organizing framework used here guided us during the organization and extraction of data by providing us with definitions of types of HRS and potential ways to categorize the data. This initial framework included the following categories: (1) collaboration; (2) governance; (3) knowledge mobilization; (4) monitoring and evaluation; (5) prioritization; (6) research infrastructure; (7) resource allocation and capacity; (8) strategic planning and (9) efficiency and waste. Working definitions of the categories and other key terms are included in Appendix A.

### Findings

We identified 5336 sources in the MEDLINE and Embase databases, and through grey literature (*n* = 53) and citation searches (*n* = 23). Of these, 1727 were duplicates, leaving 3609 sources for title and abstract screening. During title and abstract screening, 3492 sources were removed as per our exclusion criteria. The full texts of the remaining 117 sources were reviewed and an additional 52 sources were excluded. In total, 65 sources were included in the final analysis (inclusive of 16 grey literature sources). PRISMA diagram of the screening process is included in Appendix B. Full information for each study organized by responses, lessons learned and recommendations are included in Appendices B, C and D, respectively.

Findings were summarized across the organizing framework categories with the removal of “(9) efficiency and waste” as it did not have findings associated with it. In the next section we provide an overview of the pandemic responses, lessons learned and recommendations, as identified within the included sources. A detailed description of all sources and findings can be found here.

#### Collaboration

A key aspect of the HRS response to the COVID-19 pandemic within the included sources was collaboration – at multiple levels (local, national and international) and across disciplines, including examples of the University of Alabama at Birmingham (UAB) COVID19 Collaborative Outcomes Research Enterprise (CORE) [7] and the World Health Organisation [8]. Furthermore, several local collaborative efforts revealed their intention or practice of sharing data globally to improve rapid response times to the ever-changing threats of the pandemic [9, 10]. High-level governance structures and policies were crucial for coordinating collaborative research, given the unprecedented nature of the pandemic [8, 11].

Cross-sector collaboration was demonstrated by the National Institutes for Health (NIH), which engaged organizations across industry and government [12, 13], as well as communities [34], to achieve mutually beneficial outcomes. The University of Alabama at Birmingham (UAB) established the COVID-19 Collaborative Outcomes Research Enterprise (CORE) [7]. While some initial challenges to inter-disciplinary collaboration were described, such as perceived competition between clinical practice and research, these conflicts diminished over time and with more collaborative activities [14].

Funding was a facilitating factor in fostering innovative collaborations in the HRS during COVID-19. One such example, led by the National Institute of Health Research (NIHR) Oxford Biomedical Research Centre (BRC), played a significant role in supporting COVID-19 response work, including the development of the Oxford vaccine, trials and infection surveys. The BRC was a partnership between the University of Oxford and Oxford University Hospitals National Health Services (NHS) Foundation Trust that significantly supported COVID-19 response efforts, including the development of the Vaxzevria vaccine (AstraZeneca/Oxford), the RECOVERY trial and the ONS Coronavirus Infection Survey. This partnership highlights the value of research funding and infrastructure for supporting collaboration during health crises [15].

#### Collaboration: lessons learned

From the sources, we identified the following lessons learned pertaining to improved HRS collaboration during future pandemics and/or public health emergencies:

Strengthen partnerships and trust with local communities: Reducing barriers to collaboration is essential for maximizing its potential [16]. Collaboration should include not only scientific and industry partners but also communities, particularly those most affected by the pandemic. Building trusting relationships with diverse communities should be an ongoing effort, not only during public health emergencies [17, 18]. Engaging communities, particularly underserved groups, is vital. Digital technologies can facilitate broader participation in research, ensuring that diverse perspectives and needs are considered in pandemic responses [19].

Strengthen academic and clinical partnerships: The development of learning health systems that integrate medical and academic centres is essential for effective health emergency responses [7]. National and international collaborations are also necessary [20, 21]. Novel collaborative efforts to bring together interdisciplinary teams from various disciplines, institutions and nations were crucial during the COVID-19 response [22]. Partnerships between academia and industry are essential for effective research responses and should be supported through cross-sectoral collaborations and fellowship programs [23].

Improve data sharing: Effective information sharing across jurisdictions through open data, common core metadata and data-sharing platforms, as well as leveraging existing infrastructure, support research and mitigate resource waste, thereby providing a more targeted and effective response [7, 9, 24]. National strategies to invest in and expand shared research resources, such as data platforms, are necessary [25].

Improve coordination and communication to streamline research efforts: Coordinated decision-making and resource allocation are necessary for responding to public health emergencies. Eliminating system fragmentation, reducing duplication and involving experts from research communities, community representatives and funding agencies are essential for effective research efforts [18, 26, 27].

#### Governance

The COVID-19 pandemic underscored the critical role that governance structures played in guiding and facilitating research initiatives. Effective governance, characterized by the ability to manage and coordinate research activities rapidly and in a multidisciplinary manner, supported the navigation of pandemic-induced disruptions [9]. As an example, the University of Alabama at Birmingham’s (UAB) COVID-19 Collaborative Outcomes Research Enterprise (CORE) exemplified a well-coordinated governance model employing a multilayered structure and a learning health system (LHS) framework [7]. The adaptability of governance structures was also demonstrated by the NHS, which streamlined research processes in response to the crisis [28]. However, systemic inefficiencies in governance processes also persisted, as evidenced by the Canada Research Coordinating Committee’s (CRCC) ongoing fragmentation issues [29]. Similarly, smaller organizations faced challenges aligning with national governance systems, highlighting the need for flexible approaches [14]. Overall, effective governance remained a work in progress throughout the pandemic, emphasizing the need for continued development of adaptive structures to navigate complex research landscapes.

#### Governance: lessons learned

The establishment of clear governance structures played a significant role in streamlining resource allocation and prioritizing research topics to address the urgent needs arising from the pandemic [26]. The sources described how the governance of the HRS supported navigating the complexities of COVID-19 response efforts. Effective coordination across various entities and systems, both nationally and internationally, was essential to ensure a cohesive and efficient approach to research processes, particularly in the face of multi-jurisdictional health emergencies [23, 30]. Moreover, the pandemic underscored the importance of reducing barriers and accelerating processes within governance frameworks to swiftly mobilize research funds and expedite ethical approval procedures for critical studies, including clinical trials [9, 31].

Additional lessons on the critical role of governance structures in shaping the trajectory of health research responses during pandemics included the following:(1) formation of clear governance structures; (2) coordination of governance across entities and systems; (3) reduction of barriers and acceleration of processes; (4) involvement of diverse communities and consideration of data rights; (5) readiness; and (6) adaptability.

Formation of clear governance structures: Strong leadership and explicit governance structures were required to efficiently coordinate efforts across research groups, institutions and health systems. Governance of health research also played a significant role in the prioritization of topics and resources [26]. The importance of coordinating efforts across research groups and institutions, as well as health systems, was a key component of efficient governance [26]. To achieve strong leadership, sources suggested that governance structures need to be clearly defined in terms of positions, roles, responsibilities and accountability [32]. A “whole-of-government approach” applied to the advancement of science policy and research governance at the national and international levels was suggested as a means of enhancing preparedness, responsiveness and recovery from public health emergencies [33].

Coordination of governance across entities and systems: Coordination across multiple levels of government is necessary to support research processes in an effective manner, particularly during health emergencies that are multi-jurisdictional in nature [23]. In Canada, it is important to engage federal and provincial/territorial governments [30]. Coordination efforts can focus on the harmonization of communication, responsibilities, research infrastructure, platforms and programming across jurisdictions [23, 30].

Reduction of barriers and acceleration of processes: Strong and efficient governance structures were required to accelerate research processes to rapidly respond to the challenges posed by the pandemic. One such area was the swift mobilization of research funds during the pandemic [31]. In addition, the expedition of the research ethics approval procedures was critical to the initiation of research studies, including recruitment for clinical trials [26]. To address the research challenges posed by the COVID-19 pandemic, it was necessary to prioritize the establishment of oversight and steering committees for the research initiatives undertaken [9].

Involvement of diverse communities and consideration of data rights: The pandemic revealed the need for systematic data collection that was inclusive and comprehensive to inform tailored responses to public health challenges [17]. Future governance strategies should promote community trust and inclusivity to effectively respond to emergent situations.

Readiness: It will be important to ensure that the research enterprise is ready to respond to future pandemics through strategic planning. This includes ensuring alignment of the existing research system (considerations for funding mechanisms, existing research infrastructure and building and maintaining collaborative networks) [9, 19, 25, 32, 34] and ensuring that research is embedded as a central component of relevant organizations [14]. Efficient governance structures were required to accelerate research processes and address challenges posed by the pandemic. Involving regulators and establishing clinical guidelines may help establish an effective response.

Adaptability: The urgency of COVID-19 required that research systems operate in “real time” to support the use of evidence for the pandemic response [35]. Research systems had to maintain agility in their response within the context of working in rapidly evolving political, social and environmental conditions, necessitating that researchers adapt as part of this process [36]. COVID-19 has highlighted the importance of maintaining flexibility through research response mechanisms and strategies, with adaptations made to suit current needs [22]. This flexibility is counter to usual research system practices, where there is often a misalignment between institutional practices and values versus what is required to support a public health emergency [37]. Funding mechanisms had to be adapted to support rapid research responses [19, 27].

#### Knowledge mobilization

During the COVID-19 pandemic, various knowledge mobilization responses were rapidly initiated to facilitate evidence and knowledge sharing. In Canada, CIHR and PHAC collaboratively hosted Virtual Investigator Meetings to facilitate dialogue between funded researchers and federal departments on research challenges and opportunities, while CIHR also supported government partners through virtual Best Brains Exchanges (BBE), providing access to evidence for pandemic response efforts. Key international examples included the National Institutes of Health (NIH) “Director’s Blog” [13], the American Society for Microbiology's COVID-19 Research Registry [37] and Europe's Population Health Information Research Infrastructure (PHIRI). These initiatives developed rapid research platforms and established communities of practice for sharing evidence, clinician expertise and experiences through workshops, webinars and professional groups. Notable communities of practice in the UK included The Intensive Care Society, the National Deterioration Forum and the Oxford Academic Health Science Network’s “Getting It Right First Time” programme, which provided guidance for non-COVID-19 services [38]. Knowledge mobilization activities varied across regions globally, targeting diverse audiences such as local communities, health professionals and researchers.

In response to rapidly changing health system demands, the University of Alabama established the UAB COVID-19 Collaborative Outcomes Research Enterprise (CORE) to develop an institutional learning health system (LHS) guided by the knowledge to action framework (KTA) [7]. CORE integrated patient and provider data analysis with institutional research capabilities, fostering scientific collaboration, innovation and knowledge sharing. Further, a notable KM platform was the WHO’s “The Hive”, which was developed to support communities by building partnerships and promoting local knowledge and expertise [39]. The Hive utilized machine learning to gather information via web crawling, community-uploaded resources and suggested reliable sources, providing customized outputs based on user queries.

#### Knowledge mobilization: lessons learned

During the COVID-19 pandemic, the urgent need for timely and evidence-based decision-making became evident, underscoring the crucial importance of real-time knowledge mobilization. Despite extensive KM efforts, evidence on the impact and outcomes of these responses is limited. The sustainability and scalability of COVID-19-specific KM initiatives for future pandemic preparedness remain uncertain. The included sources identified the following lessons learned related to KM: (1) fostering rapid response; (2) enhancing knowledge mobilization and decision-making amid uncertainty; and (3) adaptable knowledge systems.

Fostering rapid response: Investments in implementation science, rapid learning systems, mass public participation in research and data infrastructure were recognized as essential to ensure that research-based evidence could be accessed and applied rapidly to inform public health responses [21, 35, 40]. The development of national databases and cross-jurisdictional research platforms was noted to be needed to assist with future research responses [31]. Investments in building research capacity – particularly in low-resourced areas – was identified as being required to support impactful research [41].

Enhancing knowledge mobilization and decision-making amid uncertainty: Sources identified the need for simpler and more streamlined approaches to disseminating and applying research findings, ensuring that solutions can be quickly understood and implemented by decision-makers and practitioners. Furthermore, the pandemic highlighted the importance of communicating uncertainty in scientific knowledge in a transparent manner [35]. Future knowledge mobilization efforts will need to emphasize the continuum of evidence and provide context to facilitate informed decision-making in rapidly evolving situations.

Adaptable knowledge systems: Systems for generating and using research knowledge need to be designed to adapt quickly to changing circumstances, incorporating both supply–push and demand–pull approaches [40]. The pandemic also highlighted the importance of maintaining successful research infrastructures, networks and registries outside of pandemic contexts. The development of national databases and cross-jurisdictional research platforms was also recognized as essential for supporting future research responses.

#### Monitoring and evaluation

Lessons from past infectious disease emergencies informed monitoring and evaluation strategies during the COVID-19 pandemic. For example, the US Centers for Disease Control and Prevention (CDC) and the Department of Agriculture collected gene sequencing data to evaluate human and animal viruses, monitoring public health professionals working in veterinary services for illness symptoms indicative of transboundary animal diseases [42]. Additionally, the Organization for Economic Cooperation and Development (OECD)’s Fundstat platform, established prior to the pandemic, was used to monitor government-funded R and D, tracking data on project types (e.g. vaccine development, virus transmission) and funding amounts [23]. Another approach involved adapting the Grading of Recommendations Assessment, Development and Evaluation (GRADE) framework to appraise pandemic-focused evidence rapidly and iteratively, underscoring the need for continuous evidence evaluation [43].

#### Monitoring and evaluation: lessons learned

The lessons learned related to monitoring and evaluation in health research systems emphasized several key points, including: (1) comprehensive data tracking and analysis and (2) accelerating practice-based evidence.

Comprehensive data tracking and analysis: Regular and comprehensive tracking and analysis of health research and development data can highlight progress and identify areas for improvement [24, 31]. Monitoring is important to ensure that adequate funding is provided to strengthen health research systems across nations [44]. Despite the need for rapid research for real-time decision making, caution and care are necessary when reviewing evidence, especially for policymaking, to minimize duplication and waste, while promoting collaborative and interdisciplinary work [18].

Accelerating practice-based evidence: Real-time examples of effective monitoring and evaluation, such as the partnership between OSHIN practice based research network (PRBN) and the BRIDGE-C2 Centre during the COVID-19 pandemic, demonstrate the benefit of pairing implementation science centres with research networks to facilitate rapid evaluation and generation of practice-based evidence [45]. The collaboration with the emerging BRIDGE-C2 Centre expedited research efforts, enabling real-time evaluation of practice changes and generating valuable evidence for future primary care strategies. This highlighted the crucial role of practice-based research networks in supporting cutting-edge research and informing healthcare practices through real-world evidence.

#### Prioritization

The COVID-19 pandemic necessitated rapid research prioritization to address urgent therapeutic and vaccination needs. Early initiatives, such as those by the UK Research and Innovation (UKRI) and the Department of Health and Social Care (DHSC), issued research calls in February 2020 and later expanded them to include disciplines such as social sciences and anthropology [28, 36]. This cross-governmental coordination was informed by the Scientific Advisory Group for Emergencies (SAGE).

Rapid mobilization efforts were evident globally. The UKRI issued swift research calls, Canada's funding opportunities were promptly created, and the United Nations (UN)’s roadmap was informed by rapidly mobilizing researchers [27, 36, 46]. These efforts led to the rapid identification of new therapies, such as dexamethasone [26]. However, tight timelines and balancing research commitments with clinical demands posed significant challenges [14, 28]. National funding initiatives, such as those of the CIHR, supported diverse COVID-19 research areas, outlining the value of collective action and knowledge sharing [27, 28].

#### Prioritization: lessons learned

The COVID-19 pandemic highlighted research areas that were prioritized for rapid investigation, including the development of vaccines and therapeutics, as well as public health initiatives [26]. This was possible through the creation of mechanisms for setting research priorities involving collaboration between different levels of government [20, 47]. Moreover, research results were often disseminated promptly to inform decision making among health care providers and policymakers [36]. Such focused responses to the demands that arose were necessary within short timelines [27, 41]. Therefore, there was a need for the identification of issues at the ethical, legal and social levels, as well as the development of trust at the “science–policy–society” interface [47]. Furthermore, with scarce research resources available, it was important to reduce the duplication of studies [48].

To prepare for future health emergencies, learning includes establishing processes to proactively allow for the rapid evaluation of research priorities, review of funding proposals and research needs [30]. These learnings include:

- Existing scientific networks should be mobilized within short timeframes, while groups of topic experts and other interested parties should be established to allow for quick consultations when such a need arises [19].
- The establishment of standardized mechanisms to guide critical research processes [30], including study approval and recruitment, can be expedited due to the pressing demand for information [19].
- Pre-established processes and frameworks from the pandemic can be leveraged post-pandemic, as well as in response to future health emergencies [19]. To assist with this, standardized mechanisms should be set up to guide critical research processes within rapid timelines.
- Clinical Trial Networks should be stabilized and sustained to maintain large-scale global clinical trial networks for swift mobilization of resources.

In addition, several key priorities for future pandemic preparedness of HRSs were identified, including prioritizing open science initiatives, increasing the adoption of FAIR data principles [47], making sustainable investments in research, improving the viability of research processes, increasing the accessibility of resources and ensuring that taxpayer returns on research investment are optimized [25]. To warrant that these lessons regarding priorities remain relevant and important, short-term (5 years) and long-term (10–15 years) impact assessments of funding and investment in research infrastructure were recommended [49].

#### Research infrastructure

The NIHR identified infrastructure as a critical focus for enhancing its core workstreams [35]. Their strategic plans aimed to boost patient and public involvement in research by enabling studies in real-world settings, thereby increasing staff participation and fostering industry collaboration. The NIHR intended to develop new infrastructure and support innovators, particularly UK-based small and medium-sized enterprises, to stimulate economic growth and advance health technologies. It recognized the infrastructure’s role in translating discoveries, building capacity and skills and supporting other funded research.

The key aspects of the NIHR strategy included the following:

- Multidisciplinary working and collaboration centres: promoted collaboration across academia, the healthcare system and industry, fostering a holistic approach to research and knowledge mobilization.
- Health protection research units: focused on areas such as healthcare-associated infections and antimicrobial resistance, generating evidence to inform public health policies.
- Patient Safety translation centres: disseminated research findings into practical applications to improve patient safety, bridging the gap between research and practice.

#### Research infrastructure: lessons learned

The research response to the COVID-19 pandemic necessitated the rapid mobilization of research infrastructure, including the expedited sharing of knowledge, materials, equipment,and data across disciplines [33]. To support research on COVID-19, many HRSs established special access channels to expedite the use of their equipment and services to streamline access and allow researchers to utilize specific instruments and services without undergoing the usual lengthy evaluation procedures. This necessitated the development of new processes to swiftly assess the quality of research proposals, especially considering the influx of proposals from non-traditional research areas. The pandemic also highlighted the areas of research infrastructure that needed to be strengthened to ensure preparedness for future responses to public health emergencies [33]. These included:

- *Recognition and support*: governments should consider research infrastructure as an asset for crisis preparedness, response and long-term resilience.
- *Systemic approach*: investment decisions that incentivize and promote the development of research infrastructures, capacities and relationships are needed to address challenges raised by public health emergencies to optimize resilience.
- *Engagement*: relevant research institutes and user communities should be included in developing strategies and policies for addressing crises and complex societal challenges.
- *Long-term agreements*: establishing long-term agreements and securing funding commitments with national RIs are advised to proactively integrate emergency response activities into operations.
- *Adaptation and flexibility*: rapid evolvement of operations to address shifting priorities and accommodate new users during the pandemic. This included operating at a distance, providing virtual access for users and dealing with a shortage of specialized personnel.
- *Resilient research systems*: long-term commitment to supporting and funding research capacity and infrastructure at the national level is needed, involving funding agencies and governments [15, 32].

In addition, a greater understanding of the economic impact and value generation potential of the research infrastructure is needed [15]. The public’s support for building research infrastructure is crucial and should be obtained.

#### Resource allocation and capacity

Resource allocation and capacity responses to the COVID-19 pandemic primarily involved the rapid mobilization and redistribution of funding to address urgent research needs. Initiatives such as the US “Operation Warp Speed” (OWS) and Canada’s COVID-19 Immunity Task Force exemplified this approach, with the former focusing on vaccine development and the latter on funding seroprevalence studies [17, 37]. A study on US COVID-19 research funding identified US$ 12.59 billion spent across 11 886 projects, with a significant focus on biomedical topics such as therapeutics and vaccines, while social science research received less funding [16].

The strength of pre-existing research infrastructure facilitated the rapid mobilization and redistribution of funds for COVID-19 research [26]. The strength of pre-existing research infrastructure facilitated the rapid mobilization and redistribution of funds for COVID-19 research [26]. Key examples included the NIHR and the NIHR Biomedical Research Centre (BRC). [15, 31, 50]. Additionally, resources such as the Canadian COVID-19 Genomics Network (CanCOGen) matched expertise and resources to project needs, as well as developed data portals through collaboration with universities, provincial labs and cloud service providers [33].

#### Resource allocation and capacity: lessons learned

The lessons learned related to resource allocation and capacity building underscore several critical challenges and considerations for HRSs to better respond to future pandemics, including the following:

Funding challenges: The pandemic has exposed funding gaps within the health research landscape, particularly for rapid research on COVID-19 and its mitigation strategies. Researchers faced financial hardships due to halted or delayed projects, and health charities experienced reduced donations, impacting funding for non-COVID-19 research. There was concern about potential funding cuts post-COVID-19, which could hinder the ability of the health research system to address future public health emergencies effectively [22]. This could have potential long-term impacts on development budgets, direct donations and government and charitable grants. Addressing these challenges will be essential for ensuring the sustainability of funding for health research [51].

Impact on non-COVID-19 research: Redirecting funding towards COVID-19 research has affected non-COVID-19 research projects, potentially leading to delays and long-term implications for health outcomes. Understanding these impacts is crucial for ensuring the resilience of HRSs and maintaining progress in addressing broader health issues beyond the pandemic [22]. HRSs need to balance strategic research with open research for innovation and discovery during emergencies.

Need for advocacy and resilience: To support the resilience of the HRS, strategies should focus on advocating for integrated and shared data, ensuring consistent access to quality data and promoting adherence to Indigenous research principles [17]. Addressing funding gaps and potential cuts is essential to sustain ongoing health research efforts and ensure preparedness for future public health emergencies. The response capacity of the HRS should be enhanced through practical response plans, interhospital coordination and resource management systems.

Prioritize investment in HPSR*:* Investments in health policy and systems research (HPSR) are crucial, as highlighted by the increase in funding calls until 2019. The reversal of this trend in 2020 due to the redirection of resources to COVID-19 assessments underscores the importance of understanding and addressing this decrease in funding [21]. Adequate funding is necessary to strengthen health research systems across nations [44]. Investment in key areas such as the health workforce, laboratories, data systems and risk communication is needed. The response capacity of the HRS can be enhanced through practical response plans, inter-hospital coordination and resource management systems.

#### Strategic planning

The NHS’s 2021 strategic plan exemplifies a comprehensive approach to addressing the evolving landscape of health and social care research, focusing on capacity building, responsiveness to changing needs, post-pandemic recovery and promoting equality, diversity and inclusion. The key strengths of this framework include collaboration, innovation and impact tracking. However, potential implementation challenges, resource constraints and the need for focused research priorities were also identified [46].

In British Columbia, the Strategic Research Advisory Committee (SRAC) facilitated effective communication between researchers and government decision makers, successfully identifying provincial COVID-19 research priorities. However, early efforts lacked a patient perspective and an equity lens, and coordination issues persisted [11]. Similarly, the WHO's strategic planning emphasized legal frameworks for health research, national strategic plans and health research management forums to strengthen regulatory frameworks and strategic planning within HRS [52].

Key strategic planning activities facilitating rapid COVID-19 research responses included substantial research funding from various federal and provincial sources, rapid research competitions, alignment with provincial priorities, establishment of strategic committees and networks, development of research frameworks and streamlining existing processes such as ethical review and data access protocols. These initiatives aimed to enhance collaboration, prioritize research efforts and expedite evidence generation to support the health system’s pandemic response.

#### Strategic planning: lessons learned

Lessons from strategic planning during the pandemic offer valuable insights for policymakers, researchers and stakeholders and can be summarized under the themes of collaboration and communication, inclusion and equity, rapid response mechanisms and regulatory frameworks.

Collaboration and communication: Effective communication and collaboration between researchers and decision-makers are crucial for timely and relevant research outcomes. The pandemic highlighted the importance of breaking down silos between different sectors, fostering a culture of openness and ensuring that information flows seamlessly. Regular interdisciplinary meetings, transparent data sharing and integrated digital platforms were instrumental in aligning goals and facilitating swift, coordinated actions.

Inclusion and equity: Early integration of patient perspectives and equity considerations is essential to ensure comprehensive and inclusive research approaches. This means actively engaging underserved communities, incorporating diverse viewpoints in study designs and ensuring that research benefits are distributed equitably. Addressing health disparities and recognizing social determinants of health are fundamental to building trust and achieving more universally applicable research results.

Rapid response mechanisms: The ability to rapidly establish research teams and expedite review processes is vital for addressing emergent health threats. This involves developing flexible funding mechanisms, streamlining ethical and regulatory approvals and creating emergency task forces that can quickly pivot to emerging priorities. Such mechanisms ensure that critical research can proceed without unnecessary delays, which is crucial during public health emergencies.

Regulatory frameworks: Strengthening legal and regulatory frameworks enhances the overall strategic planning and management of health research systems. Robust frameworks provide clear guidelines for conducting research, protecting participant rights and ensuring that ethical standards are met. They also facilitate international collaboration by harmonizing regulations across borders, enabling a more effective global response to health crises.

## Discussion

The COVID-19 pandemic had an unprecedented impact on society, including the health research systems (HRS) used to fund critical research that informs recovery, A full list of recommendations are included in Table 1. stabilization efforts and planning for future public health emergencies. Therefore, it is essential to understand the lessons learned from HRS responses. This paper contributes to this emerging body of evidence by summarizing the published and grey literature on the HRSs’ responses and lessons learned from the COVID-19 pandemic using an environmental scan approach.

**Table 1:** List of recommendations

Category	Recommendations
Knowledge mobilization	Investment in KM: Allocate resources to enhance knowledge dissemination mechanisms, innovation diffusion and research translation strategies. Strengthen the capacity to disseminate critical information and best practices to stakeholders, improving response capabilities during future health crises [10, 35] Data availability and integration: Improve data sharing practices by promoting early posting of research protocols and results. Develop standardized protocols for data sharing, implement data integration platforms and foster a culture of open science to enhance research, surveillance and decision-making [35] Trust and public commitment: Engage communities through transparent communication, involve them in research design and promote citizen science initiatives to build trust, enhance participation and promote responsible data sharing. This approach strengthens community resilience and facilitates effective health emergency responses [48] Technology for mass public participation: Involve the public in research activities to increase the diversity of perspectives and enhance research relevance. Utilize technology, such as smartphone applications, to capture patient-generated data and enable mass public participation. Invest in technology infrastructure, ensuring data privacy and security and promoting user-friendly interfaces for public engagement [35]
Collaboration	Global coordination and accountability: Collaborate with institutions such as the WHO and global health systems to enhance pandemic preparedness and response on a global scale [42] Partnerships with private institutions: Diversify funding sources for health research systems by collaborating with private institutions and charitable foundations, especially in regions like Latin America and the Caribbean [18, 44] Enhancing research tools and methods for collaboration: Invest in research tools, methods and standards to expedite pandemic response. Utilize technologies like artificial intelligence (AI) to improve collaboration and embrace agile editorial formats to foster transparency and trust in research outcomes [40] Collaboration with implementation science centres: Partner with implementation science centres to accelerate research efforts and identify effective healthcare strategies during public health crises [45] Community engagement and digital technologies: Strengthen community engagement and use digital technologies to lower barriers to collaboration, ensuring diverse perspectives and needs are considered in pandemic response efforts [19] Exchange of good practices and science-industry collaborations: Promote collaboration among research infrastructure managers and foster partnerships between academia and the private sector to optimize resource allocation and accelerate crisis response. Agile funding mechanisms can address conflicting priorities and incentivize rapid research efforts [19]
Monitoring and evaluation	Review and revise indicators for decision-making: Update indicators related to health research and development to align with evolving global health priorities. This ensures research investments meet current needs and improve decision-making processes [24, 45, 53] Improve data collection and monitoring: Systematically collect and monitor key health research indicators. This enables effective resource allocation, informed policy decisions and enhanced preparedness for future health emergencies [13, 53] Encourage routine reporting on health research indicators: Promote regular reporting on health research and development indicators to track progress, ensure accountability and support evidence-based decision-making [41, 54] Support global surveillance: Enhance local and global surveillance efforts for early detection of emerging pathogens. This reduces the risk of widespread transmission and strengthens preparedness and response capabilities [13, 24] Transparent processes and feedback loops: Implement transparent processes, establish expert panels and incorporate feedback loops for setting research priorities and monitoring progress against research agendas [50, 55]
Research infrastructure	Develop research infrastructure for crisis response: Establish mechanisms to enable proactive mental health research responses to emerging crises. Invest in infrastructure designed for crisis preparedness and resilience and promote collaboration among diverse stakeholders [18] Recognize and support research infrastructures: Invest in both human and technological capacities to ensure research infrastructures (RIs) can respond effectively to emergencies. Support RIs with innovations in technology and governance to enhance resilience and adaptability [16] Coordinate national, regional and international research infrastructures: Coordinate efforts among RIs to address global science needs and regional priorities, leveraging complementary strengths and resources [47] Maintain successful infrastructures: Sustain existing infrastructures, such as clinical research networks and patient safety translation centres, to ensure readiness for both minor and major health threats. Prioritize ongoing maintenance and operational support to preserve essential capabilities and collaborations for efficient responses to future health emergencies
Prioritization	Stabilize clinical trial networks: Maintain and sustain large-scale global clinical trial networks for rapid resource mobilization and international collaboration [13] Involve experts and networks: Engage experts and inclusive independent networks to address systemic issues and develop tailored pandemic response strategies [18] Align funding with global health priorities: Coordinate research and development (R and D) funding to align with global health priorities for effective resource allocation [23] Encourage coordination and data sharing: Promote coordination between funding bodies and prioritize alignment with public health needs [56]
Governance	New governance mechanism: Establish a new governance structure to complement the existing Canadian Research Coordinating Committee (CRCC), focusing on enhancing research coordination and rapid response to pandemics [29] Involvement of regulators and establishment of clinical guidelines: Engage regulators early to avoid delays and establish trustworthy clinical guidelines to translate research findings into practice in real time [13] Integration with healthcare systems: Integrate health research directly into healthcare systems to ensure seamless translation of research into clinical practice, improving patient outcomes during pandemics [26]
Resource allocation and capacity	Increased funding for health policy and systems research (HPSR): Invest in research that informs health policy and system improvements and broad research across disciplines such as virology, genomics and epidemiology to enhance pandemic preparedness [13, 44] Investment in research infrastructure: Financially support research infrastructure, including ethics boards, research coordinators, standard operating procedures (SOPs) and lab services. Creating dedicated funding mechanisms and incentivizing hospitals to grow research programs are also recommended [50] Prioritizing core capacities: Invest in the health workforce, laboratories, data systems and risk communication to strengthen health research systems. This includes training skilled personnel, upgrading labs and improving data and communication strategies [42] Improving response capacity: Enhance HRS response capacity by developing practical response plans and inter-hospital coordination systems to manage resources efficiently during emergencies [42] Addressing brain drain: Provide training in research methodology, ethics and priority-setting to enhance research capacity in low- and middle-income countries (LMICs) and other regions, and invest in educational programs to retain local talent [41]
Strategic planning	Develop a comprehensive and coherent national health research system (NHRS) strategy that includes: • Context analyses: Conduct thorough analyses to understand the specific challenges, needs and opportunities within the national health research system • Comprehensive strategies: Address governance, financing, capacity-building and research production processes through multifaceted strategies • Stakeholder engagement: Involve policymakers, researchers, healthcare providers, patients, community representatives and international partners • Monitoring and evaluation: Implement tools to track progress and assess the impact of NHRS strengthening initiatives • Develop partnerships: Form collaborations with international organizations, academic institutions, NGOs and other stakeholders for resource mobilization, knowledge exchange and collaborative research [41] Long-term planning for broader health system implications: Establish long-term plans to address the broader implications of pandemics on health outcomes, health equity and overall health system performance Anticipate and plan for post-pandemic health, social and economic policies to mitigate long-term impacts [27] Alignment of research system for pandemic research studies: • Ensure the research system is prepared to develop, fund and conduct pandemic research studies • Incentivize platform studies for rapid evaluation of multiple interventions against new infectious diseases [19] Develop national strategies for research infrastructure management • Align infrastructure investments with national research priorities and ensure efficient resource allocation • Develop clear guidelines for infrastructure management to optimize resource use and enhance research capacity [32]

This paper identifies the underlying organizational, programmatic and governance structures that were required in some high-income countries to support an effective research response to the pandemic, which can be used to inform decision-making and enhance preparedness for future public health emergencies, including pandemics. It provides critical insights by identifying key responses implemented during the crisis, the lessons learned from these responses. Each entry captures a specific aspect of the pandemic response, ranging from knowledge mobilization and data integration to governance and research infrastructure enhancements.

### Recommendations

In this section, we provide several recommendations for stakeholders within health research systems (e.g. policymakers, government, researchers, funding administrators) Recommendations are based on our analysis of included sources. Implementing such recommendations may strengthen the response of the health research system during future public health emergencies that evolve rapidly and require a well-coordinated and collaborative research response.

- Adaptive funding practices: Governments should prioritize creating flexible funding mechanisms that can support rapid mobilization of resources, which has proven crucial during the pandemic. Such a mechanism includes the ability to quickly allocate funds for urgent research needs and ensure sustained investment in non-COVID-19-related research to maintain overall health system resilience.
- Cross-industry partnerships: The pandemic highlighted the necessity of interdisciplinary collaboration to address complex health challenges. Research systems should promote partnerships between different scientific disciplines, as well as between academia, industry and public health organizations[13]. Such collaborations can enhance the comprehensiveness and applicability of research outcomes.
- Community engagement in research: Engaging communities in research is crucial for generating relevant and applicable findings. The inclusion of patients, the public and under-represented populations ensures that diverse perspectives are considered and that the research benefits are distributed equitably [17].
- Data-sharing policies: The pandemic underscores the necessity for robust data integration and sharing practices. Policies should promote the early posting of research protocols and results, the development of standardized protocols for data sharing and the implementation of data integration platforms. Open science initiatives and a culture of transparency are critical to facilitating rapid research and decision-making during health emergencies.
- Governance frameworks: Effective governance characterized by the ability to rapidly manage and coordinate research activities across multidisciplinary teams was pivotal during the pandemic. Policies should focus on establishing clear governance frameworks that can efficiently coordinate efforts across various entities, ensuring a cohesive and unified response to public health crises. This includes engaging different levels of government within a jurisdiction, such as the federal and provincial/territorial/state governments to harmonize communication, responsibilities and resource allocation.
- Investment in knowledge dissemination tools and strategies: The rapid response to COVID-19 emphasized the importance of real-time data and knowledge mobilization in informing public health decisions. Investment in infrastructure that supports the rapid generation, dissemination and application of evidence is vital. This includes the development of robust data integration platforms and the promotion of open science initiatives to facilitate real-time sharing of research findings.
- Strategic planning: The development of comprehensive strategic plans that include context analyses, stakeholder engagement and monitoring and evaluation tools is crucial for strengthening health research systems [41]. Strategic planning should also anticipate and address broader health system implications, including health equity and overall system performance. The ability to adapt operations quickly to changing circumstances was a key lesson from the pandemic. Health research systems should be designed to incorporate flexibility in their operations, allowing for rapid shifts in priorities and resources as needed [22]. Effective coordination and collaboration across local, national and international levels are critical. This includes fostering partnerships and aligning efforts across various stakeholders to optimize resource use and accelerate response efforts [19].

### Implications

The COVID-19 pandemic had an unprecedented impact on society and lessons learned from its impacts are being examined across multiple systems, including HRSs, for recovery and stabilization and to support planning for future public health emergencies. This paper contributes to this emerging body of evidence by summarizing the published and grey literature on the HRSs’ responses and lessons learned from the COVID-19 pandemic using an environmental scan approach.

#### Implications for policy

The COVID-19 pandemic has highlighted critical areas for policy improvement to strengthen HRSs. Key policy implications from the environmental scan include the need for enhanced funding mechanisms, streamlined governance structures and improved data-sharing practices. Investments in HPSR are essential, particularly for ensuring preparedness and responsiveness to future health emergencies [41]. Governments must prioritize creating flexible funding mechanisms that can support rapid mobilization of resources, which has proven crucial during the pandemic. Such a mechanism includes the ability to quickly allocate funds for urgent research needs and ensure sustained investment in non-COVID-19-related research to maintain overall health system resilience. Effective governance characterized by the ability to rapidly manage and coordinate research activities across multidisciplinary teams was pivotal during the pandemic. Policies should focus on establishing clear governance frameworks that can efficiently coordinate efforts across various entities, ensuring a cohesive and unified response to public health crises. This includes engaging different levels of government within a jurisdiction, such as the federal and provincial/territorial governments in Canada, to harmonize communication, responsibilities and resource allocation. The pandemic underscores the necessity for robust data integration and sharing practices. Policies should promote the early posting of research protocols and results, the development of standardized protocols for data sharing and the implementation of data integration platforms. Open science initiatives and a culture of transparency are critical to facilitating rapid research and decision-making during health emergencies.

#### Implications for research

The rapid response to COVID-19 emphasized the importance of real-time data and knowledge mobilization in informing public health decisions. Investment in infrastructure that supports the rapid generation, dissemination and application of evidence is vital. This includes the development of robust data integration platforms and the promotion of open science initiatives to facilitate real-time sharing of research findings. The pandemic highlighted the necessity of interdisciplinary collaboration to address complex health challenges. Research systems should promote partnerships between different scientific disciplines, as well as between academia, industry and public health organizations [13]. Such collaborations can enhance the comprehensiveness and applicability of research outcomes. Engaging communities in research is crucial for generating relevant and applicable findings. The inclusion of patients, the public and under-represented populations ensures that diverse perspectives are considered and that the research benefits are distributed equitably [17].

#### Implications for health research systems

The results of this environmental scan are intended to assist the CRPPHE with the development of a strategic prioritization and investment plan. They are intended to provide the CRPPHE with information related to the functions and mechanisms that are necessary to foster an emergency-ready health research system in Canada.

Health research systems must enhance their capacity for rapid response through strategic planning and investments in infrastructure. Maintaining resilient research infrastructures, such as clinical trial networks and knowledge mobilization platforms, is essential for swift activation during emergencies [45]. Continuous support and maintenance of these infrastructures ensure readiness for both minor and major health threats. The development of comprehensive strategic plans that include context analyses, stakeholder engagement and monitoring and evaluation tools is crucial for strengthening health research systems [41]. Strategic planning should also anticipate and address broader health system implications, including health equity and overall system performance. The ability to adapt operations quickly to changing circumstances was a key lesson from the pandemic. Health research systems should be designed to incorporate flexibility in their operations, allowing for rapid shifts in priorities and resources as needed [22]. Effective coordination and collaboration across local, national and international levels are critical. This includes fostering partnerships and aligning efforts across various stakeholders to optimize resource use and accelerate response efforts [19].

## Strengths and limitations

This review analysed 65 full-text sources from national and international academic bodies and organizations. It presents a wide range of health research system responses to the pandemic, including those related to collaboration, governance, infrastructure, knowledge mobilization, monitoring and evaluation, prioritization, research allocation and capacity, and strategic planning. The report provides information on efforts conducted at various levels of research governance, thereby supplying a strong evidence base for health research systems to form their own pandemic preparedness guidelines. Due to the heterogeneity of the included sources, the results are presented in narrative form. Furthermore, the sources included in this review did not undergo an assessment of the quality of their information. It is important to keep in mind the limitations of the review’s methodology when forming conclusions about global health research system responses. The review followed established guidelines for its methodology with a library scientist determining and conducting the literature search. Due to its rapid nature, however, the review process included a single screening of the abstracts and full-text sources, which may have led to some sources being missed. The reporting structure for the report was determined based on established processes with a data extraction tool developed by the research team.

It is possible that certain published reports were missed if their organization did not make the list of reviewed websites, or if the report was not well tagged for Google searches. Also, the grey literature search was targeted at countries expected to have the most relevant lessons learned for Canada. Although the list included international organizations, the government websites were those of mostly English-language countries, including Canada, the United Kingdom, the United States and Australia, thereby excluding publications in other languages. However, the literature search had no language limitations. Nonetheless, it was determined a priori that the focus of the environmental scan would be on countries that have similar research infrastructure and systems to those of Canada. Last, not all national and international organizations make their material publicly available on their websites, and as such, this information is missing from this report.

## Conclusion

The results of the environmental scan suggest that several next steps may be taken by governments to recognize and support research infrastructures as critical assets for crisis preparedness and response for future public health emergencies. This includes incentivizing and promoting the development of infrastructures, capacities and relationships required to address cross-cutting grand challenges and to optimize resilience. HRSs may develop a systemic approach to integrating research infrastructures and user communities to establish strategies and policies for addressing crises and complex societal challenges. Collaborating with relevant stakeholders was a prominent finding in the review and could indicate a starting point for HRSs by working together to develop strategies and policies for crisis response. Establishing long-term agreements and securing funding commitments with national research infrastructures can enable the proactive integration of emergency response activities into HRS operations. Enhancing interdisciplinary collaboration within research infrastructures is key to effectively responding to pandemics. Collaborations across scientific disciplines and sectors can facilitate a coordinated and comprehensive response to health crises. By acting on these next steps and more, health research systems can enhance their preparedness and response capabilities for future pandemics, ensuring a more effective and coordinated approach to managing public health emergencies.

Due to the absence of formal evaluations of HRS responses, the results of this environmental scan suggest that more evaluations and assessments of HRS responses to the COVID-19 pandemic (and their accompanying lessons learned and recommendations) will soon emerge. Conducting further environmental scans will be imperative. These scans may delve into the broader landscape surrounding health research, exploring factors such as funding mechanisms, institutional capacities, policy frameworks and collaboration networks. By further investigating these elements, decision-makers and researchers can gain a more comprehensive understanding of the contextual factors influencing the effectiveness and efficiency of HRSs during a public health emergency. Additionally, these scans will help identify potential gaps, challenges and opportunities within the research ecosystem, paving the way for informed decision making and targeted interventions to enhance research capabilities and outcomes.

## Supplementary Information

Supplementary Material 1.

## Data Availability

Data will be accessible in supplemental appendixes. No datasets were generated or analysed during the current study.

## Abbreviations

CIHR: Canadian Institutes of Health Research
DHSC: Department of Health and Social Care
HRS: Health Research System
IHSPR: Institute of Health Services and Policy Research
KM: Knowledge mobilization
KTA: Knowledge to action framework
LHS: Learning health system
NHRS: National Health Research Systems
NHS: National Health System
NIHR: National Institute for Health Research
NIH: National Institutes of Health
OECD: Organization for Economic Cooperation and Development
BRC: Oxford Biomedical Research Centre
PHIRI: Population health information research infrastructure
SAGE: Scientific Advisory Group for Emergencies
SRAC: Strategic Research Advisory Committee
CRPPHE: The Centre for Research on Pandemic Preparedness and Health Emergencies
UKRI: UK Research and Innovation
WHO: World Health Organization
