# Supplementary material for: Building Resilient and Responsive Health Research Systems:Responses and the Lessons Learned from the COVID-19 Pandemic
Source: Health Res Policy Syst. 2025 Mar 26;23:38. doi: 10.1186/s12961-024-01229-0 (PMC11938576; doi:10.1186/s12961-024-01229-0)
Supplement: Supplementary file 1 — Supplementary Material 1. [file 12961_2024_1229_MOESM1_ESM.docx]

### APPENDIX A: Definitions of key terms

Below are the definitions of key terms used in the report. Unless otherwise cited, definitions were created by research team after exploration of the health research systems and their role in the COVID-19 pandemic.

**Citizen science**: involve the public in the research process including data collection and analysis to generate genuine scientific outcomes (Fraisl et al., 2002)

**Collaboration** The intentional involvement and collaboration with diverse stakeholders, including communities affected by the emergency. It emphasizes an equity lens to ensure fair representation, participation, and consideration of the unique needs and perspectives of different population groups.

**Data Governance:** Data Governance is a system of decision rights and accountabilities for information-related processes, executed according to agreed-upon models which describe who can take what actions with what information, and when, under what circumstances, using what methods. (Data Governance Institute, 2020)

**Health research system (HRS):** “the people, institutions, and activities whose primary purpose in relation to research is to generate high-quality knowledge that can be used to promote, restore, and/or maintain the health status of populations; it should include the mechanisms adopted to encourage the utilization of research.” (Hanney et al., 2020, p.2).

**Efficiency and waste:** The assessment of how resources (financial, human, and technological) are utilized in the health research system during emergencies. Efficiency involves maximizing the output and impact of research activities while minimizing waste and redundancies, ensuring cost-effectiveness in achieving objectives.

**Governance:** The formalized systems, structures, and processes that define roles, responsibilities, decision-making authority, and accountability within the health research system during emergencies. Governance structures establish a framework for effective coordination and oversight.

**Knowledge mobilization:** The systematic and strategic efforts to translate, disseminate, and apply research findings into practice. Knowledge mobilization activities aim to make research accessible, relevant, and impactful for a wide range of stakeholders, including policymakers, healthcare practitioners, and the public.

**Mechanisms of a HRS response:** operational structures, processes, and strategies put in place to effectively address and mitigate the impacts of public health emergencies or crises through research activities (Dalkin et al., 2015). These mechanisms encompass various components such as knowledge mobilization, collaboration frameworks, data governance protocols, funding mechanisms, governance structures, infrastructure development, capacity building initiatives, monitoring and evaluation frameworks, prioritization strategies, and strategic planning approaches.

**Monitoring and evaluation:** Ongoing and systematic processes of collecting, analyzing, interpreting, and disseminating data related to the health emergency. Monitoring and evaluation activities provide real-time information to guide decision-making, resource allocation, and the implementation of public health interventions.

**Principles of a HRS response:** fundamental beliefs or values that guide the actions and decisions of stakeholders involved in health research during a crisis or public health emergency (World Health Organization, 2022). These principles are based on best available evidence related to best practice, ethical considerations, and lessons learned from previous responses to similar situations. They encompass various aspects such as knowledge mobilization, collaboration, transparency, equity, funding, governance, infrastructure, capacity building, and effectiveness.

**Prioritization:** The systematic process of identifying, assessing, and ranking the most critical and urgent research questions and objectives during a health emergency. It involves considering the potential impact of research on public health outcomes and resource allocation.

**Public Health Emergency:** A present or imminent event or phenomenon, of a temporary, acute nature, with actual or potential significant negative impacts on human health, resulting in: 1) A serious disruption of the functioning of a geographic, social or other community; and/or 2) The declaration of a state of emergency by a municipal, provincial/territorial, federal or Indigenous government, or international partner/s; and that, 3) Requires prompt research action to address the immediate/acute health impacts. (Government of Canada, 2023)

**Research Infrastructure:** Research Infrastructure includes the built environment and supporting elements: equipment, access, information technology (IT), systems and processes, sustainability initiatives and staff.

**Resource Allocation and Capacity:** the process of distributing financial, human, and material resources to support various aspects of health research within a given system or organization. This includes allocating funds for research projects, Research Infrastructure development, capacity building, personnel salaries, equipment procurement, and other related expenses. It may involve strategic planning, budgeting, and other approaches that ensure that resources are used effectively to address key health challenges, promote scientific discovery, and improve healthcare outcomes.

**Strategic Planning:** Strategic planning is the continual organizational endeavor of leveraging existing knowledge to articulate a business's intended trajectory. It involves prioritizing endeavors, efficiently distributing resources, aligning stakeholders and employees with the organization’s objectives, and validating these goals with data and reasoned judgment (Cote, 2020).

**Systems-level Perspective:** a focus on evaluations of the health research system as opposed to evaluations of research investments by project or initiative.

### APPENDIX B: PRISMA Diagram of Screening Process

### APPENDIX C: Table of HRS Responses to Pandemic

| **Author, Year** | **Category** | **Description** | **Outcomes**  **Strengths or Successes**  **Limitations or Challenges (if described)** |
| --- | --- | --- | --- |
| Anderson 2022 | Collaboration | To respond to the COVID-19 pandemic, University of Alabama at Birmingham (UAB) established the UAB COVID-19 Collaborative Outcomes Research Enterprise (CORE) to develop an institutional learning health system (LHS) as a coordinated response to the COVID-19 pandemic, at a local level. CORE laid out four fundamental principles, encompassing: (a) enabling data access and offering research assistance to investigators, (b) preventing redundant efforts and minimizing burdens on patients and participants, (c) promoting interdisciplinary collaborations, and (d) endorsing thorough and transparent research processes to promptly generate solutions for COVID-19. The Learning Health System (LHS) framework facilitated the seamless integration of various academic and medical centers into a unified institutional-level learning system. | Intentionally, the LHS framework incorporated members from diverse disciplines who had not collaborated before, drawing from both the health system learning and academic research sectors.  Before the onset of the COVID-19 pandemic, certain components of the LHS were already established at UAB, with various clinical sectors successfully implementing localized LHS practices that encouraged continual dialogue on the interplay between healthcare delivery and research. Leveraging these pre-existing programs within the LHS framework offered a tangible framework to steer the COVID-19 CORE in executing robust and high-quality research studies, aligning with the structure and functions associated with the LHS. |
| Ayano Ogawa 2019 | Collaboration | Health research systems have prioritized investment in One Health research to improve understanding of the emergence, prevention, detection, and control of pandemic influenza viruses. Additionally, there has been a focus on strengthening coordination between public health and veterinary services and engaging in joint preparedness planning to enhance collaboration between human and animal health sectors. | |
| Bandera 2020 | Collaboration | In response to COVID-19 in Italy, central research governance of a leading hospital experienced a number of challenges, including: (a) managing numerous research proposals from different groups focused on the same patients; (b) coordinating ethical considerations in the absence of standardized procedures and approved treatments; (c) gathering relevant bio-specimens for basic science and translational research initiatives; and (d) reallocating staff from their usual research tasks to COVID-19-related projects aligned with public health objectives. In response, they promptly established a COVID-19 steering committee to guide institutional research endeavors. Subsequently, they created an institutional registry, the COVID-19 Network, designed to address current and future research inquiries centered on the epidemiology and clinical aspects of the disease. A secondary aim of the registry was to establish a biobank of biological samples necessary for research.  The authors highlight the importance of data sharing in order to exploit the full potential of the registry created. Following the lead of similar initiatives, they plan to share their data with research groups worldwide. | This hospital's response to the COVID-19 outbreak was swift and well-organized to effectively guide institutional research activities on COVID-19 in an integrated and multidisciplinary manner. In addition to medical personnel, 15 data managers, one biostatistician, and one ethicist were engaged in this initiative. They report that at the time of publication, 103 studies across various patient groups are at different developmental stages, including 58 observational, 19 non-pharmacologic interventional, and 6 pharmacologic interventional studies. Their ethics committee convenes daily to evaluate the scientific and ethical merit of the studies.  Furthermore, they have extended access to the registry to several regional and national centers, facilitating data collection for institutions with limited research resources and fostering a network of diverse hospital units. To date, over 984 patients have been enrolled, with 641 observed at the coordinating center. Additionally, more than 1000 biological samples have been preserved. Researchers from this study group have authored 112 research articles on COVID-19, either currently in press or already published.  The COVID-19 Network is also poised to serve as a platform for future national and international multicenter studies and investigator-initiated satellite projects.  **Strengths:** The authors identify several instrumental factors that have facilitated research activities. These include: (1) effective leadership across various disciplines, proving pivotal in managing COVID-19 patients; (2) utilization of IT resources such as RedCap, i2b2, and teleconferencing systems; (3) seamless coordination among different research units; (4) engagement of data managers and technical personnel dedicated to tasks like data collection, sample collection, processing, and storage; (5) collaboration with other research institutes and clinical centers; and (6) the strong motivation and altruism exhibited by clinical, research, and support staff.  **Weaknesses:** They report that obstacles were encountered due to administrative challenges associated with document creation and approval processes. Additionally, the scarcity of data within clinical electronic records necessitated significant human effort, particularly from data managers, for the input of detailed information. Furthermore, the execution of interventional pharmacologic studies posed challenges, primarily due to the insufficient preclinical and clinical data available for many proposed drugs, as well as the centralized nature of drug trials at the national level. |
| Blomberg 2020 | Collaboration | ELIXIR's strategy is based on the support given by its national nodes to research projects and on partnering with community initiatives to drive development and adoption of good data practice and community-driven standards.   ELIXIR is collaborating with a range of stakeholders, including national and European infrastructures, research institutes, and community initiatives, to develop a federated European data ecosystem for COVID-19 research. ELIXIR's strategy builds on the developing COVID-19 actions across its member states and supports the convergence of these into interconnected European Data Spaces for COVID-19 research.   ELIXIR is also collaborating with H3Africa Bionet to understand how resources such as usegalaxy.eu can provide additional support for African researchers. ELIXIR will continue to work with appropriate community initiatives to help connect researcher-driven initiatives with the emerging European data spaces. | |
| Collins 2022 | Collaboration | Accelerating COVID-19 Therapeutic Interventions and Vaccines (ACTIV). This was a private-public partnership set up to support clinical trials for therapeutic agents for COVID-19. ACTIV also influenced the master protocols for vaccines. | States that 20 companies, multiple NIH institutes, the CDC, and the FDA all came together in this partnership, and no one was interested in who received credit. |
| Collins 2023 | Collaboration | The paper highlights several major cross-sector initiatives led by the NIH and its partners, including efforts to develop vaccines, therapeutics, and diagnostics, as well as initiatives to promote diversity in clinical trials and to provide guidance to healthcare workers.  The NIH also worked closely with international partners to share knowledge and resources, emphasizing the importance of international coordination in addressing public health challenges. The NIH-led research response to COVID-19 involved thousands of creative researchers, administrators, and community partners who were supported by much-needed resources and provided with rapid, free access to decades of discoveries made by their scientific forebears .  Overall, the collaborative response of the NIH and its partners was characterized by a shared commitment to the tenet that diversity is an essential feature for an effective rollout of a vaccine, and the groups developed and implemented new approaches for promoting inclusive participation in research in communities of color . The NIH-led research response to COVID-19 provides a model for future collaborative efforts to address global health crises. | 1. Rapid Development of Vaccines: The collaborative efforts led to the rapid development of mRNA vaccines with more than 90% efficacy in preventing symptomatic disease and an excellent safety record, leading to emergency use authorization from the US Food and Drug Administration (FDA) within 11 months of the pathogen's identification.  2. Diversity in Clinical Trials: Efforts to ensure diversity in vaccine and therapeutic clinical trials led to the inclusion of volunteers representing the diversity of the US population, particularly those in underserved communities, with at least 30% of enrollees in most vaccine trials being from racial or ethnic minority groups.  3. Therapeutic Trials: Prioritization of therapeutic trials for both outpatients and inpatients to find effective treatments for COVID-19.  4. International Collaboration: The collaborative response involved sharing knowledge and resources with international partners, emphasizing the importance of international coordination in addressing global health challenges. |
| Kayano 2023 | Collaboration | Enhancing scientific evidence for Health EDRM promoted global collaboration, WHO established the WHO Thematic Platform for Health EDRM Research Network (Health EDRM RN) in 2018. The network has guided research themes, influenced WHO Health EDRM research funding, and published the WHO Guidance on Research Methods for Health EDRM in 2021 through collaboration with over 100 global experts. The Health EDRM RN will continue to strengthen multidisciplinary collaboration, research capacities, and evidence translation into policy and practice, particularly in light of lessons learned from the COVID-19 pandemic and other emergencies. | |
| NHS 2021 | Collaboration | 1. Public Funders, Charities, and Industry: The document highlights the collaborative efforts with partners from across the public sector, charities, and industry to create an integrated research system that meets the needs of the public and remains at the forefront of international research. This collaboration aims to shape a cohesive and globally competitive research system, attract inward investment, and drive advancements in health research .  2. UK Research and Innovation (UKRI) and Devolved Administrations: Collaboration with UKRI and its constituent bodies, as well as the devolved administrations in Scotland, Wales, and Northern Ireland, is emphasized. These collaborations involve joint funding of programs, interdisciplinary working, and co-funding and participation in research initiatives.  3. Regulators and Bodies Responsible for Digital Technology and Data Science: The document underscores the collaborative relationships with regulators, digital technology, and data science bodies. These collaborations have been instrumental in accelerating and adapting research processes during the COVID-19 pandemic, as well as in enhancing data infrastructure and research capacity in public health and social care.  4. NHS and Other Healthcare Systems: The NIHR collaborates closely with the NHS, public health, and social care systems to streamline research processes, enhance data infrastructure, and maximize collective impact. The aim is to simplify research processes, strengthen research engagement with communities, and foster equitable partnerships with researchers and institutions in low and middle-income countries.  5. Industry and Trade Associations: Collaboration with industry, both directly and through trade associations, is highlighted to coordinate, streamline, and expedite end-to-end research processes. This collaboration supports the government's life sciences industrial strategy, strengthens the environment for commercially supported studies, and ensures the UK remains a globally attractive destination for high-quality clinical research. | Maximizing Impact: Collaboration is expected to maximize the impact of research by bringing together diverse perspectives, expertise, and resources to address complex health and social care challenges. This is expected to lead to more effective and efficient research outcomes that better meet the needs of patients, service users, carers, communities, and the public .   Improved Quality of Research: Collaboration is expected to improve the quality of research by promoting the use of rigorous research methods, ensuring that research is relevant and responsive to community needs, and enhancing the dissemination and uptake of research findings. This is expected to lead to a stronger evidence base in health and social care research and better health outcomes for patients and communities.  Enhanced Innovation: Collaboration is expected to enhance innovation in health and social care research by promoting the exchange of ideas, knowledge, and expertise across disciplines and sectors. This is expected to lead to the development of new and innovative approaches to addressing health and social care challenges, as well as the translation of scientific discoveries into new or improved treatments, diagnostics, medical technologies, and services.  Increased Efficiency: Collaboration is expected to increase efficiency in health and social care research by reducing duplication of effort, promoting the sharing of resources, and streamlining research processes. This is expected to lead to more effective use of resources and a greater impact of research outcomes.  **Strengths:** 1. Synergy and Resource Sharing: Collaboration allows for the pooling of resources, expertise, and knowledge from diverse stakeholders, leading to a more comprehensive and effective approach to addressing complex health challenges.  2. Enhanced Innovation: Collaborative efforts often foster innovation by bringing together different perspectives, skills, and resources, leading to the development of novel solutions and approaches to health research and care.  3. Improved Research Quality: By engaging a wide range of stakeholders, collaborative research initiatives can benefit from diverse insights, leading to higher-quality research outcomes and a greater likelihood of real-world impact.  4. Increased Impact and Reach: Collaborative responses can extend the reach and impact of research efforts, enabling broader dissemination of findings and more effective implementation of research outcomes.  **Weaknesses:** 1. Coordination Challenges: Managing collaborations among multiple stakeholders can be complex, leading to potential challenges in aligning goals, timelines, and resources.  2. Decision-Making Delays: Collaborative processes may involve multiple layers of decision-making, potentially leading to delays in implementing research initiatives or responding to rapidly evolving health challenges.  3. Conflict of Interest: Balancing the interests and priorities of diverse stakeholders within collaborative efforts can be challenging, potentially leading to conflicts of interest or competing agendas.  4. Resource Inequities: Collaborations may face challenges related to resource inequities among participating organizations, potentially impacting the distribution of funding, expertise, and influence. |
| Penny Cooper & Associates, 2021 | Collaboration | Early in the pandemic, the research community received guidance from the Provincial Health Officer (PHO) and leadership from the BC Centre for Disease Control (BCCDC), Michael Smith Foundation for Health Research (MSFHR) and UBC. | **Strengths:** Stakeholders commented on the strong leadership for success coordination of research, which included:   Specifically:   - PHO - in early days of the pandemic, directed the requirement for research coordination. - BCCDC - external coordination leadership of its director of research - MSFHR - offered leadership with its long-standing and respected role as a broker between research community and decision makers. - UBC - provided leadership through the strength and breadth of its research community and academic leadership. |
| Roope 2021 | Collaboration | The BRC, a partnership between the University of Oxford and Oxford University Hospitals National Health Service (NHS) Foundation Trust, has played a significant role in supporting COVID-19 response work, including the development of the Oxford vaccine, the RECOVERY trial, and the Office for National Statistics (ONS) Coronavirus Infection Survey. The BRC's funding flexibility has allowed researchers to respond quickly and effectively to uncertain major health issues, enabling them to divert resources from existing projects to address the emerging COVID-19 emergency. Additionally, the BRC's data infrastructure, such as NHS DigiTrials, has facilitated rapid analysis by linking administrative data to outcomes of participants in clinical trials. | The collaborative response to the pandemic, particularly exemplified by the National Institute for Health Research (NIHR) Oxford Biomedical Research Centre (BRC), has been crucial in addressing the COVID-19 crisis.  The collaborative response has led to significant outcomes, such as the development of the Oxford vaccine, which has been found effective in Phase II/III trials and is substantially cheaper and more easily stored than other effective vaccines. The BRC's support has also contributed to evidence from the RECOVERY trial, showing that dexamethasone reduces death by up to one third in hospitalized patients with severe respiratory complications of COVID-19. These outcomes underscore the critical role of research infrastructure in responding to the pandemic and its potential economic impact.  **Strengths:** The collaborative response has demonstrated the value of research funding and infrastructure in addressing health crises and highlights the need for continued investment in research infrastructure to better prepare for future emerging problems. |
| Whitehouse et al (2022) | Collaboration | Through three reflective discussions, seven key themes surfaced, seemingly defining the responses of research teams to the pandemic. The central principle that consistently guided every decision-making process during this period was the pursuit of balance through compassionate leadership, grounded in ethical decision-making.  Collaboration - The international research community collaborated to rapidly design, establish, and execute research using innovative methodologies, involving regular protocol changes. Initially, integrating the research response into COVID-19 care pathways proved challenging, with some clinical organizations perceiving research as an additional rather than a fundamental aspect of pandemic resolution, making it difficult for research teams to balance clinical service expectations with their capacity. As the pandemic response progressed, organizational hierarchies within the trusts at both sites became more horizontal, and traditional factors linked to operational structures, departmental responsibilities, and role limitations faded away.  Other info: Protecting staff and participants - The impact of COVID-19 on non-COVID research raised concerns for research teams regarding patients or service users involved in those studies. Staff faced moral distress due to redeployment, shielding, or exclusive focus on a single disease, leading to a sense of leaving behind important work and patient care. Staff's prolonged working hours to meet deadlines resulted in psychological ill health, prompting organizations to prioritize well-being and adopt more realistic work patterns during the later phases of the pandemic. | |
| WHO 2020 | Collaboration | Collaborative efforts with Member States and the development of policies on research for health have strengthened structures that administer and supervise how research is managed and financed, protect research participants, and ensure accountability. This has led to the establishment of laws regulating research for health, national strategic health research plans, and health research management forums, enhancing the regulatory framework and strategic planning within NHRS. | |
| Anderson 2022 | Governance | An Executive Leadership Committee and Advisory Board was established as well as a Research Coordinating Committee, Programmatic Working Groups the UAB Informatics Institute, an Oversight and Ethics Committee, a Patient and Stakeholder Advisory Group and multiple Scientific Working Groups (See figure 2, page 4). The LHS framework was applied to evaluate the governance structures. | The seven components of the LHS framework were thought to be effective at evaluating governance structures. |
| Bandera 2020 | Governance | Rapidly establish a COVID-19 research steering committee to guide institutional research activities. | Facilitation of Research Activities: The COVID-19 Network's tools and strategies, such as strong leadership, IT resources, coordination between research units, involvement of data managers and technical personnel, and collaboration with other research institutes and clinical centers, have facilitated research activities and contributed to the production of numerous research articles on COVID-19. |
| Bouchard et al., 2023 | Governance | The Canada Research Coordinating Committee (CRCC) was launched in 2017, to bring greater coordination to research-related programs and policies in Canada. | **Strengths:** Contributed to a better understanding of the needs and constraints of the various actors in the system  **Weaknesses:** Has not fully achieved harmonization and research support system remains fragmented. Its design does not resolve inefficiencies and inequalities, barriers for researchers in Canada and international partners, high levels of burden on researchers, challenges with addressing urgent needs and strategic issues (esp. during a pandemic). |
| Chinnery et al (2021) | Governance | Through changes to the data governance regulations, access to confidential patient data at NHS was allowed so that data linkage could be done for COVID-19-related research leading to important findings and paving the way for future medical research; Governance Structure - the COVID-19 pandemic brought about changes to the adjudication process and funding structure for grants. Similarly, the clinical trial approval structure was revised. The COVID-19 Therapeutics Advisory Panel was created to guide the Chief Medical Officer on the drugs to go on to Phase II and Phase III testing. Strengths - removal of duplication, inclusion of newly discovered drugs | |
| Whitehouse et al (2022) | Governance | Limitation of local autonomy - In response to national organizations establishing a COVID-19 research prioritization system, there were perceived expectations across the research system that smaller NHS trusts would need to deliver equivalent numbers of studies and participants without corresponding staffing or patient numbers, presenting challenges in practical application during each phase of the pandemic due to stretched resources and staff issues such as leave, isolation, or illness with COVID-19. | |
| NHS 2021 | Infrastructure | 1. Maximizing Opportunities for Patient and Public Involvement: The document emphasizes the importance of infrastructure in maximizing opportunities for patients and the public to influence and participate in research. This involves integrating research delivery into real-world health and social care settings, reducing barriers to staff contributing to research, and increasing collaboration across the NIHR infrastructure to drive progress in priority areas.  2. Development of New Infrastructure: There is a focus on developing new infrastructure to support public health and social care research, as well as increasing collaboration across the NIHR infrastructure to build research capacity across the country.  3. Support for Innovators and SMEs: The infrastructure response includes building capacity to help innovators, particularly small and medium-sized enterprises (SMEs), build the evidence required for the development, evaluation, and uptake of new medical technologies and diagnostics. This support contributes to stimulating wider economic growth and supporting the development and evaluation of new medical technologies.  4. Role in Translating Discoveries: The document highlights the role of infrastructure in translating scientific discoveries into improved treatments, diagnostics, medical technologies, and services. It also emphasizes the importance of infrastructure in nurturing talent, building capacity and skills, and underpinning research funded by other organizations, including UK Research and Innovation, medical research charities, and the life sciences industry. | 1. Improved Translation of Scientific Discoveries: The infrastructure response has transformed the health and social care system's ability to translate scientific discoveries into new or improved treatments, diagnostics, medical technologies, and services. This has been achieved through the provision of specialist facilities and support services that enable the design and delivery of clinical trials and applied health and social care research across the nation.  2. Enhanced Uptake of Innovations: The infrastructure response has enabled the uptake of innovations across the nation by providing a crucial environment for nurturing talent and building capacity and skills. This has been achieved through the provision of research delivery workforce and support services that enable the dissemination of high-quality research throughout the health and social care system.  3. Increased Collaboration with Industry: The infrastructure response has encouraged the life sciences industry to conduct their clinical studies in the UK by offering support in designing, setting up, and delivering high-quality, innovative research. This has enabled researchers, patients, and the public to access potential new treatments and has contributed to the growth of the UK's share of the global market.  4. Contribution to the UK Economy: The infrastructure response has made a significant contribution to the UK economy by supporting home-grown SMEs to establish themselves and thrive. This has been achieved through the provision of research expertise, specialist facilities, research delivery workforce, and support services that enable companies large and small to conduct high-quality research studies.  **Strengths:** 1. Enhanced Research Capacity: Strategic investment in infrastructure can lead to the development of new research facilities, technologies, and resources, which can enhance research capacity and support the translation of scientific discoveries into improved treatments, diagnostics, and services.  2. Improved Research Quality: Infrastructure investment can lead to the development of high-quality research facilities and resources, which can support the conduct of rigorous and impactful research studies.  3. Increased Collaboration: Infrastructure investment can facilitate collaboration among researchers, institutions, and stakeholders, leading to the development of new partnerships and the sharing of resources and expertise.  4. Support for Innovation: Infrastructure investment can support the development and evaluation of new medical technologies and diagnostics, fostering innovation and contributing to economic growth.  **Weaknesses:** 1. High Costs: Infrastructure investment can be expensive, requiring significant financial resources to develop and maintain research facilities, technologies, and resources.  2. Limited Accessibility: Infrastructure investment may not be accessible to all researchers or institutions, potentially leading to disparities in research capacity and quality.  3. Limited Flexibility: Infrastructure investment may be inflexible, requiring long-term planning and commitment, which may limit the ability to respond to rapidly evolving research needs or priorities.  4. Maintenance and Sustainability: Infrastructure investment requires ongoing maintenance and sustainability efforts, which can be challenging to maintain over time, potentially leading to the deterioration of research facilities and resources. |
| Anderson 2022 | Knowledge Mobilization | The Learning Health System (LHS) framework comprises seven organizational components, propelled by two dynamic engines—learning and research—derived from the Knowledge to Action Framework (KTA). Each component specifies areas for expert contributions, while each engine outlines the process of gathering, organizing, and disseminating evidence within the system. The university medical system acts as the learning engine by applying the action cycles of the KTA. This involves analyzing patient and provider data from clinical practice to pinpoint areas of disparity, low performance, or potential expansion. Concurrently, the university research infrastructure functions as the research engine, utilizing institutional research capabilities and applying them to specific clinical conditions or areas of interest. | A rich environment for scientific collaborations while fostering innovation and providing a unique institutional environment for knowledge sharing. |
| Briand et al. 2023 | Knowledge Mobilization | Creation of a platform (the Hive) to support communities, build partnerships, and promote local knowledge and expertise before, during, and after public health emergencies. It aims to ensure that all people everywhere have access to credible and trustworthy information, in a timely manner, in the right format. Machine learning includes a feedback loop for continuous learning. | Utilization of the platform/response to the platform is not described. The authors suggest that it will support developing and maintaining community trust, collaboration to address concerns, and sharing of trustworthy and relevant information. |
| Collins 2022 | Knowledge mobilization | Director of NIH participated in a "Director's Blog" twice weekly. During COVID-19, the Blog posts mostly focused on COVID-related knowledge dissemination in an accessible way. | |
| Forum on Microbial Threats 2023 | Knowledge Mobilization | The COVID-19 Research Registry - a platform was created to aggregate all information on COVID-19 research. | More than 155K unique visitors viewed the registry each week (between March 2020 - October 2022). The Registry hosted virtual journal clubs (over 1000 attendees across 55 countries).  **Strengths:** The success of the registry highlights the importance of professional societies and the role that publishers can play in bringing together the global community during an epidemic or public health crisis. |
| Penny Cooper & Associates, 2021 | Knowledge mobilization | Health Data Platform (HDP) had been in development for several years but accelerated during COVID-19. The platform provided rapid access to protocols and ethics review for clinical research. | **Strengths:** Getting the HDP setup required urgent collaboration between the Ministry of Health, health authorities, PHSA, and Population Data BC that worked well.  **Weaknesses:** According to individuals involved in the development of the HDP, the impact to do for researchers has been "negligible". The aim for the HDP for the future is that it will address some of the multi-institutional operational approvals that are current barriers for multi-organizational research (e.g., privacy review and approval, contracts around data access and sharing). |
| Sarmiento-SuÃ¡rez et al (2022) | Knowledge Mobilization | Creation of The Population Health Information Research Infrastructure (PHIRI) | The PHIRI allowed for the rapid mobilization of knowledge nationally and among European countries. |
| Sidhu et al., 2022 | Knowledge mobilization | The authors provide three examples of communities of practice that were mobilized during COVID-19 in the UK. All demonstrate how new clinical learning networks can rapidly deliver change when facilitated by an administrative infrastructure. (1) The Intensive Care Society (ICS) formed a collaboration with UCL Partners - an academic health sciences partnership, to share emerging clinical experience between intensivists across the UK. Held weekly webinars for ICS members for sharing emerging experiences (e.g., value of proning and early recognition of thromboembolic disease). Sessions were recorded and thematically analyzed. Within 24 hours, the findings were distributed by email to ICS members.  (see rows below for #2 and 3) | The networks generated new learning through interdisciplinary collaboration. Networks generated learnings through evidence and tacit knowledge from experts/clinicians/patients. Evaluation and research were grounded in service need, with clinical innovation driving research agendas.  **Strengths:** Clinicians stepping up and focusing on a clinical priority, bringing together multidisciplinary experts around a common priority.  - Speed of dissemination, as the result of engagement between applied Health Researchers and The networks.  - Regional NHS services provided leadership to support system-wide change  **Weaknesses:** Lack of national planning or coordination of clinical learning and innovation during initial stages of the pandemic  - No national plan to use existing administrative structures to support emerging clinical networks.  - Many of the networks developed during initial waves of COVID-19 have been discontinued (rather than continue with revised goals) because lack of ongoing national or regional NHS support and direction  - Lack of staff with expertise to support rapid evaluation of frontline innovation. Could be due to competing pressures from academic and policy worlds, while many staff were deployed to respond to national research agendas. |
| Sidhu et al., 2022 | Knowledge mobilization | (2) NIHR rapid evaluation teams provided valuable evidence such as how remote home monitoring with pulse oximetry (virtual wards) as implemented in the first wave of the COVID pandemic. Findings from the evaluation were disseminated during Fall 2020 through networks such as the COVID-19 oximetry community of practice group, the National Learning Network, and its regional forums. Some communities of practice were established from scratch, others built on established networks such as the National Deterioration Forum (NHS funded improvement community of practice). | |
| Sidhu et al., 2022 | Knowledge mobilization | (3) Oxford academic health science network and programs such as Getting It Right First Time took a learning network approach to produce guidance and resources for clinicians and health system planners for non-COVID-19 services during the pandemic. These networks synthesized existing evidence on remote evaluation and management. | |
| Whitehouse et al (2022) | Knowledge Mobilization | Reduced bureaucracy: The approval process for COVID-19 research was streamlined, with faster bureaucratic procedures and real-time data analysis, allowing swift approval of urgent public health studies by research and development departments in less than seven days. However, the rapid implementation of COVID-19 studies led to a reactive approach, resulting in a backlog in the administration and approval of non-COVID studies. Ethical challenges emerged as questions were raised about prioritizing one disease over others lacking an evidence base or facing research pauses.  Transformation of process - Conducting comprehensive feasibility reviews was challenging due to rapid approval processes, causing certain research team members to be apprehensive about participant safety. To address these concerns, despite receiving 'green light' approvals for recruitment, patients and caregivers were invited to join studies only when the research delivery teams were thoroughly acquainted with the protocol, and all safety measures were implemented. | Weaknesses: Ethical questions also arose over the prioritization of pandemic-related research over research on other diseases |
| Ayano Ogawa 2019 | Monitoring and Evaluation | Health research systems have focused on improving surveillance efforts, including the development of a Global Early Warning and Response System for trans-boundary animal diseases, and the establishment of comprehensive surveillance for infection and disease in occupational groups that work closely with animals. | |
| OECD 2023a | Monitoring and evaluation | Comprehending the scale and focus of government R&D funding responses during crises such as the COVID-19 pandemic, along with having appropriate data infrastructures, is crucial to achieving that vision. The OECD Fundstat initiative was established to address this need, prompted by the 2015 OECD Daejeon ministerial declaration and the 2016 OECD Blue Sky Forum. It aims to develop a versatile international analytical infrastructure for studying the direction of government R&D funding, utilizing data on publicly funded R&D projects to enable detailed, granular, and timely analyses of specific policy priorities through a combination of quantitative and qualitative information. | |
| Yousefi Nooraie et al (2021) | Monitoring and Evaluation | The "evidence pipeline" in research was modified to be responsive to emergent situations and the rapid need for information, as well as addressed the social aspects, including equity. The authors cite the example of how Grading of Recommendations Assessment, Development and Evaluation (GRADE) was modified to be used for evaluating evidence in response to the COVID-19 pandemic, particularly with respect to the need for a shorter timeline | |
| Chinnery et al (2021) | Prioritization | The authors describe the research prioritization that occurred during the early days of the pandemic, including the first research call of the UK Research and Innovation (UKRI)/ Department of Health and Social Care (DHSC) in February 2020, which was directed at studies of therapeutics and vaccinations. Other calls later followed, including one in March 2020 which encompassed more disciplines, including those that were never previously involved in emergency research funding calls, such as social sciences and anthropology through the Economic and Social Research Council. A subsequent call for research applications had a rolling deadline and was informed by the priorities of the Scientific Advisory Group for Emergencies (SAGE) to ensure cross-governmental coordination of efforts. In October 2020, the National Core Studies program was announced. | The funding of studies in rapid research calls. The creation of sustainable models of programs, such as the National Core Studies.  **Strengths:** The inclusion of a wide number of disciplines in the calls (including anthropology and social sciences for the first time in an emergency situation) led to the funding of projects involving various research perspectives and interdisciplinary studies, evolving with changes in the pandemic - The projects submitted to the calls were reviewed quickly, including 10 days to decision from submission for the rolling deadline call - The calls did not include limitations on budget - The calls allowed for submissions from companies and encouraged international teams - The funding for the projects was 18 months for the earlier calls and 12 months for the subsequent rolling call (versus the usual 3 to 5 years) - The adjudication process involved the usual peer review and a decision from the Chief Medical Officer to assess for the policy implications of the research - The peer review process went from in person to virtual due to pandemic restrictions - The results of the studies had to be publicly available in a short period of time, leading to an increase in preprint publications  **Weaknesses:** The 12-month research timeline requirement for proposals was more complicated to plan out with impactful research  The organization saw weaker submissions leading to a 10% funding rate despite an unrestrained budget. |
| Forum on Microbial Threats 2023 | Prioritization | Opportunities to partner with clinical research for effective pharmaceutical and non-pharmaceutical interventions. | Workshop attendees compared the US response to the UK system (having a national health care system and coordination of clinical research through RECOVERY).  **Weaknesses:** The fragmented system in the US did not facilitate coordination of efforts to understand the best therapeutic options. Attendees suggested a research platform that can coordinate, and evolve with information change, throughout the response of a future epidemic. |
| Forum on Microbial Threats 2023 | Prioritization | The workshop attendees noted the speed at the advancement of research in treatment and management. Debate within workshop attendees around the speed of research response to vaccine vs therapeutic development. guidelines. | **Weaknesses:** Comments from workshop attendees suggested that the investments in vaccine development did not match investments in therapeutics, as well as coordination efforts. |
| Hanney 2022 | Prioritization | The responses involved efforts to enhance research coordination, prioritization, and expedited ethics approval, which contributed to the rapid identification of new therapies, such as the case of dexamethasone in the United Kingdom and Brazil | **Strengths:** Efficient research coordination and prioritization contributed to the rapid identification of new therapies, such as the case of dexamethasone in the United Kingdom and Brazil, demonstrating the effectiveness of coordinated efforts within the HRSs. |
| Kayano 2023 | Prioritization | Given the progress of the Health EDRM RN and the evolving global situation, two major projects are underway: the 2nd phase of the WHO Health EDRM Knowledge Hub and the formulation of research priorities for Health EDRM. These initiatives aim to provide a broader understanding of WHO Health EDRM policy, practice, and advanced research areas crucial for disaster risk management. The WHO Health EDRM Research Network facilitates effective multidisciplinary and multisectoral collaboration for policy, practice, and research development, offering an opportunity for engagement in these endeavors. | Provided wider understanding of the WHO Health EDRM policy, practice and several advanced research area |
| McMahon 2020 | Prioritization | CIHR and its partners have launched rapid research response funding opportunities, resulting in the funding of numerous research projects focused on COVID-19. These projects cover a wide range of areas, including clinical epidemiology, mental health, substance use, and more. Additionally, the government allocated substantial funding to support these research initiatives, demonstrating a strong commitment to addressing the pandemic through research.  Furthermore, the CIHR's Institute of Health Services and Policy Research (IHSPR) has played a significant role in this response. IHSPR conducted a rapid-cycle priority identification process to identify seven COVID-19 priorities for health services and policy research, along with three cross-cutting themes. This process involved input from leading HSPR experts in Canada, a brief survey of the HSPR community, and validation with the Institute Advisory Board.  Overall, the response has been characterized by collaboration and coordination among various stakeholders, rapid mobilization of research funding, and a focus on addressing the most pressing research needs related to the pandemic. This concerted effort aims to provide evidence-based guidance to inform decision-making and policy development in Canada's health system response to COVID-19. | 1. Rapid Mobilization of Funding: The response demonstrated the ability to quickly mobilize substantial funding for COVID-19 research, enabling researchers to address critical aspects of the pandemic. 2. Collaborative Approach: The involvement of various stakeholders, including funding partners, researchers, experts, and the broader health research community, reflects a collaborative and coordinated approach to addressing the pandemic. 3. Targeted Priority Identification: The rapid-cycle priority identification process conducted by the CIHR's Institute of Health Services and Policy Research (IHSPR) allowed for the identification of specific COVID-19 priorities for health services and policy research, ensuring a focused response to the most pressing research needs.  **Strengths:** 1. Potential Overemphasis on COVID-19: While the response focused on addressing the immediate challenges posed by the pandemic, there may be concerns about the potential neglect of non-COVID-19-related research during this period. 2. Resource Allocation: There may be challenges in ensuring equitable resource allocation across various research areas, especially as the response prioritizes COVID-19-related research. 3. Long-Term Planning: The response may benefit from a more explicit emphasis on long-term planning to address the broader health system implications of the pandemic beyond immediate research needs. |
| McMahon et al., 2021 | Prioritization | UN developed a Research Roadmap for the COVID-19 recovery. The focus of this commentary is how rapid mobilization of scholars (HSIF) helped to inform the roadmap. | The roadmap was completed in a timely and robust manner because of the rapid mobilization of highly trained science and research workforce (e.g., scholars within the CIHR Health System Impact Fellowship), with the ability to rapidly distill and learn from the existing evidence base, actively engage with health system decision makers to understand evidence needs, and identify evidence-informed solutions and considerations  **Strengths:** The CIHR HSIF provided access to a cohort of trained scientists who could quickly respond and contribute their expertise to an urgent global health crisis. |
| Richardson 2021 | Prioritization | In response to the pandemic, the UK Research and Innovation (UKRI) launched a rapid response fund to support impactful research across various fields, including the humanities, social, and natural sciences. The funding landscape was designed to produce policy-relevant research, which required the academic community to work rapidly, remotely, and responsively. The authors of the article responded to this call by using rapid response research methods to study the criminal justice system's response to domestic abuse during the pandemic. They prioritized the dissemination of findings for effective and speedy impact with policymakers and practitioners rather than the publication of academic literature. The research aimed to produce findings that could be used to make changes to policy and practice, with the production of knowledge of interest to academic audiences as a secondary aim | The prioritization of rapid response research methods and the dissemination of findings for effective and speedy impact with policymakers and practitioners resulted in the production of two academic publications, four working papers, two submissions to government inquiries, a presentation to regional senior police leads, and two sets of policy recommendations within the first six months of funding. The research users, including police officers, court managers, victims of domestic abuse, and the charities that support them, required the delivery of robust recommendations so that practice could be changed and improved as quickly as possible. The fast delivery of research results and the transformation of analysis into recommendations involved concentrated work for the research team over shorter periods of time than that taken in normal academic research |
| Whitehouse et al (2022) | Prioritization | Prioritizing - Researchers had to navigate the delicate balance between their research commitments, clinical priorities, and the ongoing responsibility of caring for existing research participants. At Site 1, the rapid and extensive influx of COVID-19 admissions during the initial phase of the pandemic necessitated the immediate deployment of research staff to support frontline clinical activities. A minimal research team was retained to assist existing research participants and offer senior support for the remaining research staff. In contrast, Site 2 experienced its first COVID-19 case about two to three weeks after Site 1. This time lag allowed for a limited but valuable period of pragmatic planning, particularly in devising a tiered approach to the redeployment of research staff. It also provided an opportunity to maintain contact with participants involved in studies unrelated to COVID-19. | |
| Kayano 2023 | Resource Allocation and Capacity | The World Health Organization (WHO) has initiated efforts to develop and support the implementation of health emergency and disaster risk management (Health EDRM) globally. The Health EDRM Framework, published in 2019, outlines the essential functions across various disciplines and sectors to mitigate hazards, vulnerabilities, and exposures, ensuring effective preparedness, response, and recovery. Evidence and research play crucial roles in advancing Health EDRM, informing risk understanding, shaping health services, fostering workforce development, and facilitating monitoring and evaluation. | To ensure effective preparedness and readiness, response, and recovery provide effective multi-disciplinary and multi-sectoral collaboration for engagement in development of policy, practice and research |
| OECD 2023b | Resource Allocation and Capacity Building | The study describes the funding responses to COVID-19 R&D in terms of the allocation of government funding to various areas of research. It highlights that funding for R&D on COVID-19 was largely focused on the biomedical response to the coronavirus pandemic, with vaccines and therapeutics attracting most of the funding. Additionally, efforts to build understanding of the virus in terms of biology, diagnostics, and clinical characterization received significant funding. The study also notes that funding for social science research was significant but less than implied by the sheer number of projects. Furthermore, funding for R&D in support of platforms and capabilities appears to have been disseminated across a wider range of WHO topics | Funding outcomes are described in terms of the total government funding allocated to COVID-19 R&D projects, the average funding per project, and the distribution of funding across different topic clusters. The study reports that the 11,886 identified projects add up to a total government funding of USD 12.59 billion, with an average funding per project around USD 1.20 million. It also highlights the distribution of funding across different topic clusters, indicating that most of the funding was concentrated on biomedical topics, particularly on coronavirus understanding, therapeutics, and vaccine development. The study also discusses the co-occurrence patterns of funding, indicating the pivotal role of R&D platforms and capabilities across different areas of COVID-19 R&D |
| Ayano Ogawa 2019 | Resource allocation and Capacity Building | Efforts have been made to develop infrastructure to complement national testing with rapid international verification in WHO-certified laboratories, prioritize interventions in high-risk areas such as wet markets, and monitor the effectiveness of health and non-health interventions in real time | |
| Collins 2022 | Resource Allocation and Capacity Building | The NIH extended timelines for scientists. Specifically, they provided extensions on early-career investigator status (extending the timeline from final degree to end of early career investigator), and for the intramural program, extending the tenure-track timetable and also advocacy for other grantee institutions to do the same. | |
| daSilva 2022 | Resource Allocation and Capacity Building | COVID-19 Technology Access Pool (C-TAP). This initiative is a "voluntary pool of IP, clinical and regulatory data, know-how and other types of knowledge for the development and production of technologies for the detection, prevention, control, and treatment of COVID-19. Launched in 2020, Launched in May 2020, its goal is to accelerate the development of these technologies and their availability by mobilizing  additional production capacity and removing barriers to access  through non-exclusive licensing and technology transfer to  boost local production of generic drugs, particularly by middle-  and low-income countries." | By November 2021, C-TAP had its first licensing agreement for a COVID-19 serology antibody test (Spanish National Research Council). In May 2022, two licensing agreements were signed with the NIH (United States) focused on the development of 11 therapeutics, early stage vaccines, and diagnostic tools.  **Strengths:** The initiative consolidates existing mechanisms that have expanded to include a COVID-19 remit.  A voluntary initiative, commitment from the pharmaceutical innovation system is paramount to success. As of May 2021, the initiative was supported by 40 countries, other organizations within the United Nations system, NGOs, and individuals.   AstraZeneca supported C-TAP through bilateral technology transfer agreements.   C-TAP scored well on initial criteria (13 of 14 points).   Using the scoring criteria, C-TAP aligns with CEWG recommendations most broadly (scoring 7 out of 12 points, expanded criteria).  **Weaknesses:** The following jurisdictions were not listed as supporting C-TAP:  1) United States, 2) the European Union, 3) United Kingdom of Great Britain and Northern Ireland, and 4) Japan. Additionally, pharmaceutical companies that produced COVID-19 vaccines - Pfizer and Moderna, were also not supporting. |
| daSilva 2022 | Resource Allocation and Capacity Building | Access to COVID-19 Tools Accelerator (ACT-A). ACT-A is a collaborative, coordinated, and multi-lateral response (umbrella initiative, shared partnership model) with partnerships across global health organization, foundations, civil society, academia, and the private sector. The components of ACT-A are: 1) diagnostics, 2) treatments, and 3) vaccines (COVAX). Funding is provided by government donations, private sector, philanthropic institutions, multilateral organizations. | ACT-A received $20.5 billion (as of April 2022) - the majority (66%) of funds were allocated to the vaccines pillar. Public funds contributed 90%.  **Strengths:** Using the initial criteria, the ACT-A scored well (13 out of 14 points).  **Weaknesses:** Using the expanded criteria (CEWG) the score went to 0 of a possible 12 points. The authors found that ACT-A did not implement CEWG recommendations into its structure. |
| Forum on Microbial Threats 2023 | Resource Allocation and Capacity Building | Operation Warp Speed (OWS) which was the name for US federal spending on vaccine development and connecting researchers with federal employees. Also included unprecedented public-private partnerships for collaboration between academia and industry. | OWS had knock on impacts to the HIV research community whereby redeployment of HIV research and infrastructure to COVID-19 efforts (this negatively impacted progress in HIV research).  **Strengths:** Rapid development of COVID-19 vaccines.   Federal funding was supplied through Biomedical Advanced Research Development Authority (BARDA) and the Administration for Strategic Preparedness and Response (ASPR) (along with other organizations and agencies) to support researchers proceeding without usual delays in grant applications and receipt of awards.  **Weaknesses:** Funding that focused on vaccine development occurred at the expense of understanding transmission and other considerations of viral pathogenesis, patient needs and critical questions that still remain unresolved about COVID-19. |
| Hanney 2022 | Resource Allocation and Capacity Building | Efforts were made to build and coordinate clinical trial capacity within healthcare systems, with examples from the United States, British Columbia, and Brazil, highlighting the importance of infrastructure for clinical research and the ability to mobilize clinical researchers across the country  The responses involved the rapid mobilization of existing primary and secondary research capacity, along with enhanced interdisciplinary cooperation and clinical research integrated into healthcare systems, making important contributions to the pandemic research response. | **Strengths:** The rapid mobilization of existing primary and secondary research capacity, along with enhanced interdisciplinary cooperation and clinical research integrated into healthcare systems, made important contributions to the pandemic research response, showcasing the strength of existing research capacity within HRSs |
| Mishra 2023 | Resource Allocation and Capacity Building | Canada's resourcing response to the pandemic faced some limitations and gaps. The early COVID-19 funding left gaps in resourcing critical areas such as implementation science, qualitative or mixed methods research, and One Health or zoonotic research. This meant that some important areas of research that could have informed the public health response were not adequately supported.  However, there were also positive examples of resourcing efforts. For instance, in April 2020, Canada's national public health agency established a COVID-19 Immunity Task Force to fund seroprevalence studies, combined with data harmonization and data sharing. The task force was among the first, in August 2020, to call for research with and among communities experiencing the highest risks. Additionally, Statistics Canada began to develop a disaggregated data action plan in December 2021 to break down data according to sex, gender, race, ethnicity, age, sexual orientation, and disability and combinations of these to reveal uneven economic and social realities in Canada. | **Weaknesses:** Despite these positive examples, the authors of the study suggest that more could have been done to adequately resource critical areas of research and engage diverse communities in research efforts. The lack of person-level data collection inclusive of social determinants of health, racial identity, and exposure risks, alongside critical governance issues, demonstrated that Canada was not fully prepared to respond systematically to a pandemic shaped by health inequalities |
| OECD 2023a | Resource Allocation and Capacity Building | The Canadian COVID-19 Genomics Network (CanCOGeN) is a collaborative initiative involving federal, provincial, and regional health authorities, academia, research institutes, hospitals, and industry partners in Canada. The network focuses on sequencing and sharing viral samples from positive COVID-19 tests (VirusSeq) and the genomes of patients diagnosed with COVID-19. CanCOGeN represents a strategic investment in developing and sharing data assets to support various research activities related to the pandemic.  In terms of resource allocation, CanCOGeN has evolved its priorities to address challenges related to the effective sharing of data across Canada and internationally. The network has established partnerships with universities, provincial labs, and companies providing cloud services and expertise for data portal development, dashboard creation, and data visualization. These collaborations leverage the strengths of different stakeholders within the network, with public health labs generating data, academics contributing analytical capacity, and the private sector developing visualization tools.  By strategically allocating resources and fostering partnerships across different sectors, CanCOGeN aims to enhance data sharing, analysis, and visualization capabilities to support research efforts related to COVID-19. This collaborative approach ensures that the network can effectively leverage the expertise and resources of various stakeholders to address the challenges posed by the pandemic and contribute to the development of data-driven solutions for managing and controlling the spread of the virus. | |
| Penny Cooper & Associates, 2021 | Resource Allocation and Capacity Building | There was a rapid infusion of significant funding for COVID-19 research in BC. This included seed funding for rapid response public health questions (e.g., MSFHR, Genome BC), direct infusions of funding to research organizations, and research competitions. | **Strengths:** Rapid infusion of research funding was identified by many stakeholders in this study as the most important contributor to success of the research response. |
| Roope 2021 | Resource Allocation and Capacity Building | The research infrastructure response to the pandemic, particularly exemplified by the National Institute for Health Research (NIHR) Oxford Biomedical Research Centre (BRC), has been instrumental in addressing the COVID-19 crisis. The BRC, a partnership between the University of Oxford and Oxford University Hospitals National Health Service (NHS) Foundation Trust, has provided critical support for COVID-19 response work, including the development of the Oxford vaccine, the RECOVERY trial, and the Office for National Statistics (ONS) Coronavirus Infection Survey. | The outcomes of this research infrastructure response have been significant. The development of the Oxford vaccine, supported by the BRC, has been found effective in Phase II/III trials and is substantially cheaper and more easily stored than other effective vaccines.  **Strengths:** The BRC's funding flexibility has allowed researchers to respond quickly and effectively to uncertain major health issues, enabling them to divert resources from existing projects to address the emerging COVID-19 emergency. Additionally, the BRC's data infrastructure, such as NHS DigiTrials, has facilitated rapid analysis by linking administrative data to outcomes of participants in clinical trials. |
| Scholten 2020 | Resource Allocation and Capacity Building | Quickly secure funding for research: The CIHR institute of Infection and Immunity committed to taking $250,000 from the institute’s operating funds to launch its programs. In this process, a new model of funding for public health emergencies was established, allowing for rapid mobilization of research efforts. | |
| Singh et al (2020) | Resource Allocation and Capacity Building | The authors give examples of research activities that were suspended or deprioritized due to the COVID-19 pandemic. These included the ongoing studies by the HIV Prevention Trials Network, which were suspended while new studies were not initiated, as well as the clinical trials funded by the British National Institute for Health Research, which were suspended due to the redeployment of personnel to frontline work and the prioritization of COVID-19 research. The authors also note the impact of public health restrictions on contact with study participants. | |
| Tsang et al., 2022 | Resource Allocation and Capacity Building | In the UK, a coordinated health research system exists. Health services and health research intersect through the National Institute for Health Research (NIHR). NIHR provides the NHS with research support to coordinate efforts of networks, NHS trusts, and universities. The NHR Clinical Research Network (CRN) provides funding to support research infrastructure at individual hospital sites. It provides funding for staff, facilities, equipment, and support services for research, as well as specialist training, information system, patient and public engagement. | NIHR was successful in engaging UK hospitals in COVID-19 pandemic studies. E.g., the Randomized Evaluation of COVID-19 Therapy (RECOVERY) trial that received funding from the NIHR CRN in March 2020 as an urgent public health study. RECOVERY enrolled over 11,000 patients at 176 hospitals in 100 days. This was the first study to identify the benefits of corticosteroids in treating hospitalized covid patients, and to confirm that treatment with hydroxychloroquine and lopinavir/ritonavir had no benefits.  **Strengths:** Timeliness/pace at which urgent pandemic trial research was funded (facilitated by the existing research infrastructure, and connection with health services). Recruitment into trial and engagement with stakeholders. |
| Tsang et al., 2022 | Resource Allocation and Capacity Building | The Australia and New Zealand Intensive Care Society (ANZICS)-Clinical Trials Group support clinical studies across its 74 adults and pediatric ICUs. It includes financial support for research coordinators to enable ICUs to undergo research programs. Additionally, Australian Partnership for Preparedness Research on Infectious Diseases Emergencies (APPRISE). | |
| Tsang et al., 2022 | Resource Allocation and Capacity Building | The EU is currently building a network of 600+ primary care sites and 600 hospital sites across 27 EU member states to conduct large-scale clinical research studies on emerging infectious diseases - the Platform for European Preparedness Against (Re)emerging Epidemics (PREPARE). | No outcomes described. The aim is to build clinical trial capacity in the EU, that is available and ready to respond to future epidemics. |
| WHO 2020 | Resource Allocation and Capacity Building | Regional partnerships and consortiums of funders have provided capacity-building training on research methodology, ethics, and priority-setting, aiming to enhance research capacity in LMICs and other regions | The responses have focused on enhancing the four functions of NHRS, including stewardship and governance, financing, capacity-building, and producing and using research . This has led to improved governance and management, capacity-building initiatives, and the development of policies and research agendas, resulting in more effective and coordinated health research activities |
| WHO 2020 | Resource Allocation and Capacity Building | NHRS have demonstrated the ability to quickly mobilize research efforts to address the urgent needs posed by the pandemic. This has led to the rapid generation of scientific knowledge and evidence to inform public health responses. | |
| Chinnery et al (2021) | Stakeholder Engagement Collaboration | The Medical Research Council was established to award UK government funding for medical research per the terms of the National Insurance Act. In 2020, various components of the research landscape in the UK were mobilized to address the challenges posed by COVID-19. This involved MRC core-funded research centers and institutes with diverse missions, covering areas like virology, protein structure, disease modeling, cell biology, population cohorts, and immunology. The UK benefits from a diverse array of life science research funding sources, including government departments, arms-length bodies, non-profit organizations like the Welcome Trust, large pharmaceutical companies, and small biotech start-ups. Additionally, the government's launch of the Life Sciences Industrial Strategy in 2017 has fostered regular collaboration among senior individuals from government, funders, regulators, pharma, and biotech, facilitating effective cross-sector communication. All these entities played a significant role in addressing the challenges posed by the COVID-19 pandemic.  The pandemic clearly constituted a global health crisis, and various initiatives to compile and disseminate information internationally were significant. One illustration is the GloPID-R-UKCDR COVID-19 Research Tracker, which included all MRC awards, enhancing awareness of the funding portfolio. | |
| Elliott et al., 2021 | Stakeholders  Engagement Collaboration | Seniors Helping as Research Partners (SHARP) was established in 2013. During COVID-19, the GHS research group engaged with SHARP members through focus groups, to understanding their experiences and suggestions for research and policy priorities/planning. | This article presents findings from focus groups, but minimally discusses how these findings/how the engagement with SHARP informed research priorities, policy, planning, etc. The authors indicated that they have received funding to explore one of the themes from the focus groups, "call for increased funding and appropriate care for older adults".  **Strengths:** The existence of/partnership with SHARP prior to COVID-19 allowed for GHS to engage with older adults early on in the pandemic and later in follow up. The existing and established relationship with SHARP members facilitated open and honest conversations, according to the authors.  **Weaknesses:** The authors describe limitations related to engagement and representation - e.g., majority of SHARP members are white. The authors acknowledge compensation for patient partners is one factor motivating participation in research, but they were not in a financial position to provide this. Participants required Internet access which limited participation to those who had competency and access to virtual technology. |
| NHS 2021 | Strategic planning | 1. Building Capacity and Capability in Preventative, Public Health, and Social Care Research: The strategic plan emphasizes the need to enhance research capacity in preventative, public health, and social care domains. This involves incentivizing researchers to engage in these areas, accessing new thinking from a broader range of disciplines, and supporting career pipelines in global health .  2. Addressing Changing Health and Social Care Needs: The strategic plan focuses on accelerating the pace of development in health, public health, and social care research to fully address the changing needs of people and communities. This includes a commitment to improve ways of working, strengthen communications, and collaborate with other research funders, sponsors, and regulators to reduce unnecessary bureaucracy .  3. Supporting Recovery and Learning from the Pandemic: The strategic plan aims to build on learnings from the research response to COVID-19 and support the recovery of the health and social care system. This involves working with stakeholders across the health, public health, and social care system to ensure responsiveness to research needs and promote the adoption and diffusion of proven innovations .  4. Improving Lives of People with Multiple Long-Term Conditions: The strategic plan includes a focus on improving the lives of people with multiple long-term conditions through research. This involves increasing research in populations with high disease burden across the life-course and incentivizing able researchers to get involved in these areas .  5. Embedding Equality, Diversity, and Inclusion: The strategic plan emphasizes the need to embed equality, diversity, and inclusion across NIHR's research, systems, and culture. This involves strengthening careers for research delivery staff and under-represented disciplines and specialisms, as well as expanding work with the life sciences industry to improve health and economic prosperity . | 1. Improved Research Capacity and Capability: The strategic response aims to build research capacity and capability in preventative, public health, and social care domains, as well as increase research in populations with high disease burden across the life-course. This is expected to lead to a stronger evidence base in these fields and a greater understanding of how to affect the wider determinants of health .  2. Enhanced Responsiveness to Changing Needs: The strategic response aims to accelerate the pace of development in health, public health, and social care research to fully address the changing needs of people and communities. This includes a commitment to improve ways of working, strengthen communications, and collaborate with other research funders, sponsors, and regulators to reduce unnecessary bureaucracy. These efforts are expected to enhance responsiveness to research needs and promote the adoption and diffusion of proven innovations .  3. Improved Lives of People with Multiple Long-Term Conditions: The strategic response includes a focus on improving the lives of people with multiple long-term conditions through research. This is expected to lead to better health outcomes and reduced burden on public services .  4. Greater Equality, Diversity, and Inclusion: The strategic response aims to embed equality, diversity, and inclusion across NIHR's research, systems, and culture. This includes strengthening careers for research delivery staff and under-represented disciplines and specialisms, as well as expanding work with the life sciences industry to improve health and economic prosperity. These efforts are expected to promote greater diversity and inclusivity in health and social care research .  **Strengths:** 1. Comprehensive Approach: The strategic response outlined in the document "Best Research for Best Health: The Next Chapter" reflects a comprehensive approach to addressing the evolving challenges in health and social care research, with a focus on capacity building, responsiveness to changing needs, recovery from the pandemic, and promoting equality, diversity, and inclusion.  2. Collaborative Efforts: The strategic response emphasizes the importance of collaboration and partnership with stakeholders, including patients, the public, and other research participants, to ensure that research priorities align with community needs and values.  3. Focus on Innovation: The strategic response aims to promote innovation in health and social care research, with a focus on incentivizing researchers to engage in new areas, accessing new thinking from a broader range of disciplines, and supporting career pipelines in global health.  4. Emphasis on Impact: The strategic response emphasizes the importance of tracking progress against outcomes and adjusting course as needed, ensuring that research has a positive impact on people's lives.  **Weaknesses:** 1. Implementation Challenges: The strategic response may face challenges in implementation, particularly in terms of navigating complex governance structures and ensuring effective collaboration and partnership with stakeholders.  2. Resource Constraints: The strategic response may face resource constraints, particularly in terms of funding and personnel, which could limit the scope and impact of research activities.  3. Potential for Inefficiencies: The strategic response may create inefficiencies in research processes, particularly if overly complex governance structures or bureaucratic processes are implemented.  4. Limited Focus on Specific Areas: The strategic response may have a limited focus on specific areas of health and social care research, potentially leaving gaps in research priorities and outcomes. |
| Penny Cooper & Associates, 2021 | Strategic Planning | Leveraging of new and existing infrastructure and partnerships to address BC COVID-19 research priorities. The BC COVID-19 Strategic Research Advisory Committee (SRAC) acted as a conduit between the research community and senior decision makers in the office of the PHO. | **Strengths:** Supported communications between research and government/policy decision makers. The SRAC was respected as a forum for identifying provincial COVID-19 research priorities.  **Weaknesses:** The SRAC was critiqued by participants for its lack of patient perspective and equity lens early in its formation. This was later addressed through expanded membership but continued to be a concern for stakeholders. There was also some confusion about the purpose, scope, authority, and accountabilities of different COVID-19 research coordinating initiatives (e.g., the SRAC). Overall, much of the coordination of the provincial research response was built on existing relationships and well-positioned individuals and institutions. However, it did not achieve an integrated provincial reach and suffered the same coordination issues that existed before COVID-19. |
| Scholten 2020 | Strategic planning | Early request for proposals: the CIHR Institute of Infection and Immunity launched a request for proposals for research into the causes and consequences of SARS, initiating the first phase of SARS research in Canada. Within two weeks, 18 research teams across Canada had assembled and submitted proposals. It took another 10 days for a rapid peer review to be conducted by the CIHR’s Governing Council, with four applications being approved. | |
| WHO 2020 | Strategic Planning | The establishment of laws regulating research for health, national strategic health research plans, and health research management forums has been promoted to enhance the regulatory framework and strategic planning within NHRS | |

### APPENDIX D: Lessons Learned from HRS Pandemic Responses

| **AUTHOR, YEAR** | **Category** | **Explanation of Lesson Learned & implications** |
| --- | --- | --- |
| Anderson et al 2022 | Collaboration | The University of Alabama at Birmingham (UAB) COVID-19 Collaborative Outcomes Research Enterprise exemplified valuable lessons in collaboration to enhance health research system pandemic responses. Through rapid mobilization, interdisciplinary collaboration, and network building, UAB demonstrated the importance of inclusivity, integration, and the establishment of guiding principles for effective crisis management. The adaptability, resource optimization, and sustained engagement within the system highlighted the significance of continuous learning, improvement, and evaluation in refining response strategies. By embracing these lessons, organizations can strengthen their preparedness, responsiveness, and effectiveness in addressing public health emergencies, fostering a culture of resilience and innovation in health research systems. |
| Bandera 2020 | Collaboration | The COVID-19 Network's commitment to data sharing and collaboration with basic and clinical research groups worldwide allows for the potential of conducting national and international projects, fostering a global approach to understanding and combating COVID-19 |
| Blomberg 2020 | Collaboration | The response to the COVID-19 pandemic has prompted European countries to mobilize unprecedented public health efforts, with research being rapidly redirected to understand and combat the virus. The ELIXIR initiative plays a crucial role in supporting these research endeavors by facilitating collaboration and coordination across national infrastructures and data resources. ELIXIR provides access to cloud and storage resources specifically tailored for COVID-19 research, enabling the collaborative development of open, reusable, and reproducible computational workflows. Additionally, ELIXIR promotes the annotation and deposition of data to ensure its long-term reuse and value.    As COVID-19 research projects emerge from various initiatives driven by ELIXIR Nodes, the organization has launched a support webpage to showcase the services offered by Nodes to scientists. Moreover, ELIXIR actively participates in community-driven efforts, such as the virtual COVID-19-BH-20 BioHackathon, which brings together global bioinformaticians to collaborate on tooling for COVID-19 analysis. ELIXIR Nodes contribute resources like cloud computing and storage to support such initiatives, fostering collaboration and data sharing within the research community.    The ELIXIR strategy aligns with the directed actions across member states in response to COVID-19, aiming to develop interconnected European data spaces for pandemic-related research. By leveraging existing national and European infrastructures, ELIXIR contributes to the creation of a coordinated federated environment for secure data archival, access, and analysis, essential for European collaborations in healthcare. Furthermore, ELIXIR prioritizes the development and publication of open, reproducible tools and workflows for COVID research, emphasizing sustainability to ensure long-term preparedness for future challenges. |
| Charalambakis 2021 | Collaboration | The value of team science and interdisciplinary collaboration: SRRs play a crucial role in fostering collaborative and team-based research approaches, enabling researchers to access cutting-edge technologies and expertise that may not be available within their own institutions. |
| Charalambakis 2021 | Collaboration (ethics) | The importance of equitable access: Expanding access to SRRs to institutions that do not have access to these facilities would foster a talent pipeline across the educational landscape and ensure that untapped potential is developed and applied early in the educational process. Implications: The implication of this lesson is that policymakers and funding agencies need to prioritize equitable access to SRRs to promote diversity, inclusivity, and innovation in the scientific enterprise. |
| Chen 2021 | Collaboration | The need for coordinated efforts to ensure progress in resource allocation decision-making around health research and development, particularly in response to public health emergencies of international concern. |
| Chen 2021 | Collaboration | The need for action to capitalize on the global cooperation driven by the COVID-19 pandemic and start to make gains on the vision of a more equitable global health future. |
| Collins 2023 | Collaboration | The pandemic demonstrated the need for collaborative efforts across sectors to address global health crises, and the NIH learned the importance of sustained learning and constant preparation to effectively respond to future pandemic. Implication: The implication is the need for continued collaboration and information sharing with international partners to address global health challenges effectively. This underscores the importance of sustained international coordination and preparedness for future pandemics. |
| Demkowicz 2021 | Collaboration | Swift Response and Collaboration: The mental health research community demonstrated a swift response to the pandemic, highlighting the importance of collaboration, coordination, and streamlined systems to facilitate a rapid research response |
| Dyke & Mak, 2021 | Collaboration | . Addressing Complex Challenges: The COVID-19 pandemic is a complex challenge that requires a multidisciplinary approach. Collaboration across research teams, disciplines, agencies, and countries can help address the complex challenges posed by the pandemic. This can include sharing knowledge, expertise, and resources to develop effective interventions and strategies to combat COVID-19.  2. Expedited Innovation: Collaboration can also expedite innovation by bringing together diverse perspectives and expertise. This can lead to more innovative solutions and accelerate the development of effective interventions and strategies to combat COVID-19.  3. Optimizing Resources: Collaboration can also help optimize resources by reducing duplication of efforts and ensuring that research efforts are coordinated and aligned with the most pressing needs. This can lead to more impactful research outcomes and contribute to a more effective global response to COVID-19.  Overall, the lessons learned from the pandemic underscore the importance of collaboration in addressing complex challenges, expediting innovation, and optimizing resources. By working together across research teams, disciplines, agencies, and countries, the research community can develop more effective interventions and strategies to combat COVID-19 and other public health emergencies. |
| Hanney 2022 | Collaboration | Coordination was identified as a critical factor in the effective response of health research systems (HRSs) to the COVID-19 pandemic. The document highlights that existing or rapidly established coordination was often the key to effective responses and reduced risk of wasted resources. The pandemic response involved efforts to enhance research coordination, prioritization, and expedited ethics approval, which contributed to the rapid identification of new therapies, such as the case of dexamethasone in the United Kingdom and Brazil. The document identifies at least three broad categories of research coordination: first, pre-pandemic coordination of attempts to enhance research preparedness for a pandemic and to develop a readiness to respond; second, attempts during the pandemic to build mechanisms for greater coordination in the research response once the need was realized; third, the mobilization during the pandemic of coordination mechanisms that already existed across the HRS in line with the existing health research strategies. The study emphasizes the importance of coordination mechanisms within HRSs, including the need for effective collaboration and coordination across different research groups and institutions. The document highlights the importance of governance functions, including coordination and prioritization, which were critical to the pandemic response, emphasizing the importance of governance in focusing resources within HRSs. |
| Harrison et al 2022 | Collaboration | Community members must be equal partners (e.g., not just research participants) and involved earlier in the proposal and study development process.  **Implications:** Earlier involvement might support adjustments to research to meet community needs, consider diversity/equity lens etc. |
| Harrison et al 2022 | Collaboration | Researchers often had limited awareness of basic principles of patient-centered outcomes research and community engagement, including communication in plain language, active listening skills.  **Implications:** Highlights a need to improve research team's skills in community engagement. May support development of training materials to enhance researchers' ability to connect with community stakeholders. |
| Harrison et al 2022 | Collaboration Engagement with Stakeholders | Multicenter studies were often inflexible at meeting local investigator or community needs. This resulted in potential challenges incorporating PCAB member feedback and truly operationalizing patient-centered research.  **Implications:** The limits in flexibility of multi-center studies is a barrier to the incorporation of community feedback. |
| Harrison et al 2022 | Collaboration | Incentives for research participation were often not considered, not enough or not appropriate. **Implications:** A need exists to consider and potentially adjust research participation incentives. |
| McMahon, Nidegal et al 2020 | Collaboration | The response highlighted the importance of collaboration and coordination among various stakeholders, including funding partners, researchers, experts, and the broader health research community. This collaborative approach allowed for a more targeted and effective response to the pandemic. |
| Mishra 2023 | Collaboration | 1. Building Relationships: The study emphasized the importance of building relationships with communities before emergencies. Successful engagement strategies were built on existing patient and community partnerships with people experiencing social or economic marginalization. This highlights the need for ongoing relationship-building and trust between public health entities and diverse communities.  2. Tailored Responses: Community engagement was essential for tailoring research and data-informed local responses based on community-specific data and social determinants of health. Engaging communities allowed for the development of more tailored and effective public health strategies that addressed the specific needs and challenges faced by different populations.  3. Inclusivity and Trust: Lessons for inclusivity and trust in all aspects of pandemic research, from study design to methods, interpretation of results, and dissemination, were highlighted. Inclusive engagement strategies that incorporate diverse voices and lived experiences are crucial for building trust and ensuring that public health measures are responsive to the needs of all community members.  4. Indigenous Engagement: The study also emphasized the importance of engaging Indigenous communities in the pandemic response. Efforts to involve Indigenous communities in research and data sharing were highlighted, and the need for meaningful community engagement and governance that recognized Indigenous principles of ownership, control, access, and possession for data and biological samples was underscored. |
| NIHR (2021) | Collaboration | The pandemic response has underscored the importance of strengthening the engagement of people and communities in research. Lessons learned include the need to better use digital technologies and to reach out more effectively to communities under-served by research. This includes building on heightened awareness of research as a result of COVID-19 to strengthen engagement efforts and ensure that the perspectives and lived experiences of individuals in relation to their health and care are heard and acted upon. Implications: The implication of the lesson learned on the engagement of people and communities is the need to prioritize inclusive and community-centered research approaches. This involves leveraging digital technologies to enhance engagement, addressing disparities in research participation, and ensuring that research efforts are responsive to the needs and perspectives of diverse populations. It also highlights the importance of building trust and partnerships with communities to ensure that research is relevant and impactful |
| OECD, 2020 | Collaboration | The paper underscores the value of mutual learning and exchange of good practices among research infrastructure managers. It suggests that collaboration and joint elaboration of harmonized solutions can lead to cost sharing and cost savings. Implications: The lesson on collaboration and exchange of good practices implies that research infrastructure managers should engage in mutual learning and exchange of good practices to identify cost-sharing and cost-saving opportunities. This requires the development of networks and partnerships among research infrastructure managers to share knowledge and expertise. |
| OECD, 2023a | Collaboration | Collaboration between academia and industry is essential for addressing challenges, particularly during crises like the COVID-19 pandemic. Enabling conditions such as open data, data sharing platforms, and infrastructures serve as crucial platforms for co-creation partnerships. Initiatives like fellowship programs are effective in cultivating young talent and bridging the gap between academic research and industry needs. However, barriers to mobility, such as unidirectional career perceptions, need to be addressed to encourage more individuals to engage in cross-sector collaborations.    Government support is paramount in facilitating collaboration between academia and industry. Timely allocation of additional funding and involvement of credible institutions with long-term funding play a significant role in scaling co-creation activities. Long-term investment in discovery research is also vital for fostering pioneering scientific knowledge that can be leveraged in collaborations with industry partners.    Policy instruments and funding mechanisms play a crucial role in promoting collaboration between academia and industry. Data sharing infrastructures, public research institutions, and digital platforms are essential facilitators of collaboration, enabling the rapid dissemination and utilization of research results. Flexible funding mechanisms are particularly important during crises, as they allow for swift adaptation and optimization of cross-sectoral collaboration efforts. |
| OECD, 2023b | Collaboration | Convergence Towards Shared Data Standards: The need for funding agencies to converge towards shared data standards can enhance transparency and facilitate international comparative analysis and coordination. Implications: This convergence can lead to greater analysis uniformity, replicability, and coordination in R&D funding efforts. |
| OECD, 2023c | Collaboration | Encouraging Data Openness and Common Core Metadata: The study highlights the benefits of encouraging funding agencies to converge towards data openness and the use of common core metadata for accountability and analysis purposes. Implications: This can lead to greater transparency, accountability, and improved analysis of R&D funding efforts. |
| Office of the Chief Science Advisor of Canada, 2022 | Collaboration | The report highlights the importance of international scientific cooperation in addressing global health emergencies. Collaborating with other countries and sharing scientific knowledge and best practices can enhance the global response to pandemics and other emergencies |
| Sidhu 2022 | Collaboration | Lack of formal and sustainable linkage between NHS clinical networks and academic research and evaluation community. Implications: Because NHS clinical networks were not linked to academic research or evaluation, they did not meet the criteria for a true clinical learning network. Thus, their effect on bottom-up service transformation was diminished by top-down government demands and lack of a formal implementation partner. Additionally, there is a need for an effective alliance between academic researchers and clinical services. Alignment of rapid evaluation and applied health research is essential for robust evidence. Half of all UK research funding went to underpinning and etiology, and 5.6% to health service research. |
| Sohrabi 2021 | Collaboration | International research efforts, built on collaboration, have allowed for significant breakthroughs to be made regarding our understanding of the pandemic. The open sharing of knowledge and research efforts has stimulated global collaborative bonds with common purpose. It is our hope that these will continue beyond the pandemic, for the benefit of both education and research |
| Cochrane (2022) | Communication | The rapidly changing (highly politically charged) context and rapidly evolving evidence of mixed quality have challenged research methods, tools, processes, partnerships and communication, especially without additional resources. In particular, we have struggled to convey uncertainty, what is known (right now and what is not known (yet), and how the evidence and broader response to the pandemic might evolve” Implications: Provide education and training on crisis communication. |
| Blomberg 2020 | Data management Data Governance | Fostering good data management practice to make COVID-19 data open, FAIR and reusable over the long-term |
| Dyke & Mak, 2021 | Data sharing Governance | 1. Real-time Data Access: The pandemic has demonstrated the need for consistent and reliable access to real-time data to support decision-making for emergency response and planning. This includes data on the spread of the virus, the effectiveness of interventions, and the impact on vulnerable populations. Real-time data access can help inform policy decisions and support effective emergency response efforts.  2. Integrated Information Systems: Integrated, secure information systems are also crucial for effective decision-making. This includes systems that integrate data from multiple sources, such as health records, testing data, and contact tracing data. Integrated information systems can help identify patterns and trends, support forecasting, and inform resource allocation.  3. Ongoing Innovation: The pandemic has highlighted the value of ongoing innovation in data management and sharing practices. This includes innovations in online peer review, expedited Research Ethics Board approvals, and data sharing agreements. Ongoing innovation can help ensure that data sharing and access practices remain effective and responsive to emerging needs and challenges. |
| OECD, 2023a | Data Access Governance | Unequal Progress in Adopting FAIR Data Principles: During the pandemic, data access was hindered by varying adoption rates of FAIR data principles across scientific disciplines. This lack of universally recognized standards and coordinated systems for data collection and dissemination posed challenges, particularly in clinical and epidemiological data.  Standardization and Interoperability Issues: Structural barriers such as standardization and interoperability issues impeded the generation of internationally comparable statistics. Inconsistencies in how confirmed cases, deaths, and recoveries were reported across countries or sub-national jurisdictions made it challenging to understand the evolution of the pandemic.  Sensitive Data Collection and Use: The collection, access, and use of sensitive data, including clinical studies, patient records, and surveys, were subject to ethical and legal requirements that were not always adaptable to urgent crisis situations. Aligning data practices with ethical and legal standards proved to be a critical consideration during the pandemic response.  Global Genomic Data Infrastructures: Distributed genomic data infrastructures, in conjunction with global coordination structures like GLOPID-R and GISAID, played a crucial role in supporting research efforts related to understanding, monitoring, and mitigating COVID-19. The pandemic necessitated the adaptation of Open Science approaches to ensure safe and secure sharing of sensitive data across countries.  By addressing these lessons learned and implementing strategies to enhance data access, health research systems can better prepare for and respond to future crises effectively. |
| McMahon 2021 | Embedded research training | The mobilization and completion of the rapid scoping reviews that informed the UN research roadmap, demonstrates the value of embedded research training programs for knowledge mobilization. Implications: Cultivates rapid learning systems and networks that include researchers and decision-makers, and fosters collaboration across disciplines. |
| Cochrane (2022) | Inequity | Financial Support for Evidence Generation: Providing more financial support for evidence generation, communication, networks, and infrastructure in low- and middle-income countries is crucial to address inequities in health research systems. This support can help ensure that all regions have the resources and capacity to respond effectively to global health emergencies. Invest in building research capacity in low- and middle-income countries is essential to address inequities in health research systems. By strengthening research capabilities in underserved regions, it is possible to promote more equitable access to resources, expertise, and support during pandemics and other health crises.  Advocating for Global Evidence System: Working with national and international stakeholders to describe the ideal global evidence system and advocating for the necessary conditions can help address inequities in health research systems. By promoting a more inclusive and collaborative approach to evidence generation and dissemination, it is possible to reduce disparities and improve the overall response to pandemics.  Transparency in Decision-Making: Greater transparency about how evidence is used in decision-making processes is essential for addressing inequities in health research systems. By ensuring transparency and accountability in resource allocation and decision-making, it is possible to promote fairness and equity in the response to global health emergencies. |
| Demkowicz 2021 | Equity | Inclusivity and Co-Production: here were concerns that the need for fast, reactive research may have overlooked the role of experts by experience, highlighting the importance of co-production and patient and public involvement and engagement (PPIE) to strengthen research in various ways |
| Sohrabi 2021 | Inequity | Lockdown orders have also imposed new challenges for mothers in academia, highlighting the persistence of gender inequality in science alongside the need for equity for all. Implications: Female academies are more likely to assume parenting or domestic responsibilities in comparison to their male counterparts. With the closure of universities and research institutions, researchers have been compelled to work from home, however female researchers are more likely to shoulder the added responsibilities of childcare and home-schooling.  Female researchers have also continued to battle underrepresentation in academia and the aforementioned challenges brought by COVID-19, which may have contributed to widening of the pre-existing gender gap in research. |
| Dyke & Mak, 2021 | Cultural consideration Ethics | 1. Ethical and Cultural Sensitivity: Lessons learned from the pandemic highlight the need for clear governance structures to ensure ethical and culturally sensitive research practices, particularly when working with Indigenous communities. This includes respecting Indigenous knowledge, traditions, and cultural protocols when conducting research that involves Indigenous peoples.  2. Community Engagement: The pandemic has underscored the importance of engaging with Indigenous communities in a respectful and collaborative manner. This includes involving community members in the research process, seeking their input, and ensuring that research activities align with community needs and priorities.  3. Governance Structures: Clear governance structures are essential to ensure that research practices involving Indigenous peoples are conducted in an ethical and culturally sensitive manner. This may involve the establishment of research review processes that include Indigenous representation, as well as the development of research agreements that outline the terms of engagement and data ownership.  Overall, the lessons learned from the pandemic highlight the importance of integrating respect for Indigenous research principles and ensuring ethical and culturally sensitive research practices when working with Indigenous communities. By prioritizing cultural considerations and engaging with Indigenous communities in a respectful and collaborative manner, the research community can conduct research that is aligned with community needs and priorities while upholding ethical and cultural standards. |
| Harrison et al 2022 | Culture Improvement Ethics | Researchers must acknowledge the burden experienced by Patient and Community Advisory Board (PCAB) members and the communities they represent (e.g., higher rates of COVID-19 disease, death, and inequities) and associated triggers in these discussions that evoke their experiences with cultural, racial, ethnic, and historical trauma. Cultural humility must inform research study designs.  **Implications:** this knowledge can be used to educate research teams on cultural humility and its importance which will contribute to improved research design and stakeholder relationships. |
| OECD, 2023 | Mapping Evaluation and Monitoring | Importance of Precise Concepts for R&D Directionality: Defining and implementing precise concepts for measuring R&D directionality is crucial for accurately mapping R&D funding. Implications: This can enhance transparency, stewardship, and facilitate international comparative analysis and coordination. |
| Bandera 2020 | Governance | Rapid Establishment of Research Steering Committee: The establishment of a COVID-19 research steering committee aimed to guide institutional research activities, demonstrating a proactive approach to addressing research challenges. |
| Becerra-Posada 2021 | Governance | The study's implications include the need for whole-of-government open-data policies to facilitate access to research financing data. The study also suggests that countries need to increase their budgets and strengthen national health research systems to promote evidence-informed decisions for better health policies. The study recommends that LAC countries consider partnerships with private institutions such as charitable foundations to diversify funding sources for the development of HPSR. |
| Chen 2021 | Governance | The need for more data sharing, particularly on research and development investments and capacity, to enable better coordination and informed decisions in the global health research landscape. |
| Hanney 2022 | Governance | Governance plays a crucial role in the effective functioning of health research systems (HRSs), particularly during a global health crisis such as the COVID-19 pandemic. The document emphasizes the significance of governance functions in focusing resources, including coordination, prioritization, and the acceleration of much-needed research approvals.  Coordination: Effective coordination mechanisms were identified as a key component of governance during the pandemic response. The ability to coordinate research efforts across different institutions, research groups, and healthcare systems was essential for ensuring a cohesive and efficient response to the public health crisis.  Prioritization: The document highlights the importance of effective priority-setting within HRSs. Rapidly testing new therapies, reducing the waste of resources, and considering the needs of diverse communities were critical aspects of prioritization that contributed to the pandemic response.  Ethical Approval: The ability to accelerate ethics and protocol approvals, as well as enhance data access and sharing, increased the speed and efficiency of research production. This aspect of governance was crucial in facilitating the rapid deployment of research initiatives and clinical trials.    Evaluation: The document emphasizes the need for impact assessment within HRSs. The substantial and immediate benefits from rapid research progress provided enhanced opportunities and the need for impact assessment to understand the effectiveness of research initiatives. |
| Mishra 2023 | Governance | The governance lessons learned from the responses to the COVID-19 pandemic in Canada emphasize the importance of data governance, community trust, and models of governance across diverse communities and settings. The study highlighted the need for community-led implementation and governance at the regional and community levels in the collection, sharing, and rigorous use of socioeconomic, occupational, household, and race-based data to inform and evaluate each aspect of a pandemic response.  One of the key governance lessons learned is the importance of Indigenous self-determination and rights in informing Indigenous governance of Indigenous data and the use of those data by Indigenous leadership. The study emphasized that structures and data governance systems that were in place before the COVID-19 pandemic enabled timely sharing of relevant data and information for First Nations and Metis governance, self-determination, and decision-making. This Exemplifies the importance of respecting Indigenous rights and governance structures in pandemic responses.  Additionally, the study highlighted the limitations in data collection and governance, especially in the context of person-level data inclusive of social determinants of health, racial identity, and exposure risks. The need for systematic data collection inclusive of diverse communities and settings to inform tailored public health responses underscores the importance of inclusive and comprehensive data governance frameworks.  Implications: the governance lessons learned from the responses to the COVID-19 pandemic in Canada underscore the importance of community-led implementation and governance, Indigenous self-determination and rights, and the need for inclusive and comprehensive data governance frameworks to inform tailored public health responses. These lessons can inform future governance strategies to promote community trust, inclusivity, and effective pandemic responses |
| OECD, 2020 | Governance | Effective portfolio management requires clear governance structures that define roles and responsibilities, decision-making processes, and accountability mechanisms. This ensures that research infrastructure managers are able to make informed decisions and are held accountable for their actions. Implications: The lesson on effective portfolio management implies that research infrastructure managers should establish clear governance structures, develop national strategies, and secure adequate funding to support the management and operation of research infrastructures. This requires collaboration and coordination among stakeholders to ensure that resources are allocated effectively and efficiently. |
| OECD, 2023 (July 2023 No. 155) | Governance | "Coordination and collaboration across levels of governance". Governance systems at different levels need to collaborate, cooperate, and coordinate their actions with international organizations guiding these activities. The integration and harmonization of scientific work across jurisdictions can be improved through better infrastructure, programming, and platforms, aiding collaboration. Implications: Emergent situations often affect numerous jurisdictions and require the efforts of multiple levels of governance |
| OECD, 2023c | Governance structures | "Dynamic and system-oriented governance of science for society": the advancement of science policy and research governance at the national and international levels utilizing a "whole-of-government approach" is required for actions in the domains of preparedness, responsiveness, and recovery, as well as for increasing capacity for data collection in quantitative and qualitative form. Investments are required in policy development, policy evaluation, and policy adaptation |
| PHAC, 2010 | Governance | The review highlights the need to review and streamline the federal/provincial/territorial governance structure for pandemic influenza. This lesson underscores the importance of clarifying roles and responsibilities and improving communication channels among advisory groups within the pandemic governance structure. The review suggests considering options for the development of standardized mechanisms to facilitate the rapid conduct of critical research across jurisdictions. This lesson underscores the importance of having pre-established processes to swiftly identify research opportunities, set research priorities, and review proposals for funding during public health crises. Implications: Establishing clear and streamlined governance structures and communication channels can enhance interprovincial collaboration on health research during public health emergencies. This can facilitate the efficient allocation of research resources and promote coordinated research efforts across jurisdictions. |
| Scholten et al, 2020 | Governance | "Its application of pre-mobilized development funds that would allow for teams to immediately receive funding without delays in the usual granting process exhibited CIHR’s agility in adopting new protocols. The CIHR did demonstrate its willingness for experimentation through changes to its funding practices, as well as its  initiation of off-shoot organizations such as the PPSRI. This could serve as a reference point as to how federal and /or national organizations can maneuver within bureaucratic constraints." |
| Bandera 2020 | IT infrastructure | Multidisciplinary Approach and Platform for Future Studies: The network's focus on integrated and multidisciplinary research, including precision medicine studies with immunology, virology, and omics approaches, provides a comprehensive platform for future national and international multicenter studies and investigator-initiated projects. |
| Camille 2021 | Infrastructure | 1. Resilience is Key: The pandemic has underscored the critical importance of resilience in infrastructure systems. Building resilience across multiple dimensions, including physical, operational, financial, and governance aspects, is essential to ensure that infrastructure can withstand and adapt to future challenges.  2. Interconnectedness of Infrastructure: The document highlights the interconnected nature of infrastructure and how disruptions in one sector can have cascading effects on others. Understanding these interdependencies is crucial for building resilient infrastructure systems that can continue to function effectively during crises.  3. Adaptability and Innovation: The pandemic has accelerated the need for innovative and proactive responses to resilience challenges in infrastructure. Embracing new technologies, business models, and approaches to infrastructure planning and management is essential for overcoming the current crisis and preparing for future ones.  4. Importance of Social Infrastructure: The crisis has brought renewed focus on the importance of social or "soft" infrastructure, which includes elements like healthcare, education, and social services. These infrastructures are critical for maintaining societal standards and responding effectively to crises.  5. Investment in Preparedness: The document emphasizes the need for increased investment in pandemic preparedness and response infrastructure. Countries, especially emerging and developing economies, need to bolster their health response capabilities to limit the spread of viruses and mitigate the impact of future pandemics. |
| Demkowicz 2021 | Infrastructure | 1. Fragmentation and Overlapping Priorities: The research response showed fragmentation and an overlapping emphasis on specific priorities. Efforts to develop and expand cross-institutional and interdisciplinary networks should be undertaken to support greater collaboration and more efficient resource deployment. Registries can aid coordination of activity, but they should be carefully coordinated to avoid duplication.  2. Need for Emergency-Ready Infrastructure: The infrastructure was not built for emergencies, despite calls to do so over a decade ago. The pandemic highlighted ongoing weaknesses in the infrastructure, emphasizing the need for leadership and coordination going forward. Efforts should be made to develop infrastructure that can better facilitate research responses to future emergencies.  3. Streamlined Systems: The pandemic highlighted the weaknesses of the research infrastructure, but also demonstrated the benefits of streamlined systems. Funders and HEIs should develop sustainable models with more straightforward and timely application processes while preserving quality and rigor in the review process. Streamlined systems can reduce burden for researchers and contribute to more efficient research efforts.  4. Coordination and Collaboration: Efforts to enrich existing infrastructure with an emphasis on collaboration, interdisciplinarity, and cross-sector partnerships are essential. Building longer-term mechanisms that allow the mental health research community to act proactively in the face of emerging crises is crucial. Funders should play a more active role in ensuring strategic deployment of resources and coordination of research efforts. |
| Hanney et al, 2020 | Enhancing Research Infrastructure and Culture Infrastructure | Enhancing Research Infrastructure and Culture: Lessons learned from interactions and synergies between research infrastructure and culture highlight the need for a conducive environment that supports system-level change in health research. Implication: Efforts should be directed towards fostering a research culture that promotes knowledge generation, patient benefits, and economic growth, while delivering effective health and social care |
| OECD, 2020 | Diversity of National Systems Infrastructure | The paper acknowledges the diversity of national systems and research infrastructure operation approaches. It emphasizes that there is no single model to suit every country or research infrastructure, and that guiding principles should be tailored to specific contexts. The lesson on the diversity of national systems implies that guiding principles for research infrastructure management and operation should be tailored to specific contexts. This requires a flexible and adaptable approach to research infrastructure management and operation that takes into account the unique characteristics of each national system. |
| OECD, 2020 | Infrastructure | One of the key lessons learned from the OECD Science, Technology and Industry Policy Paper on Optimizing the Operation and Use of National Research Infrastructures is the importance of effective portfolio management for national research infrastructures. Implications: The lesson on the importance of national research infrastructures implies that governments and funding agencies should prioritize investments in research infrastructures to support national research communities and address global challenges. This requires a long-term commitment to funding and support for research infrastructure management and operation. |
| OECD, 2023a | Infrastructure | 1. Mobilization of Research Infrastructures (RIs): Research infrastructures played a crucial role during the COVID-19 pandemic by providing equipment, materials, data, and expertise across various scientific domains. The rapid mobilization of RIs highlighted their potential as critical assets in national and international crisis preparedness and response. However, the effectiveness of RIs in crisis situations is dependent on the approaches taken by science policymakers and funders.  2. Need for Scalable Operations: Investing in the human and technological capacity required for crisis preparedness and response is essential to ensure that RI operations can be quickly scaled up when necessary. This shift in focus from short-term financial efficiency to long-term resilience is crucial for enhancing the responsiveness of HRS to future pandemics.  3. Enhanced Connectivity and Collaboration: Policy actions focused primarily on funding biomedical and life science RIs during the pandemic, neglecting additional support for other RIs to adapt their operations. To improve response capabilities, there is a need for greater connectivity and harmonization between RIs to facilitate collaboration across different disciplines and stages of the research and development pipeline. Coordinated and interdisciplinary research efforts are essential for effective crisis response.  4. Adaptation to Changing Environments: The pandemic necessitated the adaptation of RI operations to address new priorities and accommodate new users. RIs had to rapidly adjust to operating at a distance and providing virtual access for users. Limited technical capacity required RIs to invest resources in training for their staff and the wider community, highlighting the importance of building technical expertise and resilience within RIs. |
| Roope 2021 | Infrastructure | The document highlights the lessons learned from the COVID-19 pandemic regarding the value of research infrastructure in healthcare systems. It emphasizes the perils of overemphasizing short-term efficiency at the expense of preparedness for unpredictable crises. The concept of "option value" is introduced, emphasizing the potential benefits of investing in spare capacity and infrastructure for emergencies, even if the actual need is uncertain. The commentary discusses the role of the National Institute for Health Research (NIHR) Oxford Biomedical Research Centre in responding to the pandemic, particularly in supporting critical COVID-19 response work such as the development of the Oxford vaccine and the RECOVERY trial.    The lessons learned include the importance of research infrastructure funding flexibility in enabling rapid and effective response to emerging health crises. The document also stresses the need for a comprehensive assessment of the value of research infrastructure, particularly in the context of pandemic preparedness. It highlights the potential economic impact of research infrastructure, such as the Oxford vaccine, on global and national economies, underscoring the significant value it can generate. Furthermore, it suggests that understanding the public's willingness to invest in resilience-building infrastructure is essential for policymakers to make informed decisions.  In conclusion, the document calls for a better understanding of the option value of research infrastructure and the public's willingness to invest in it, particularly in the aftermath of the COVID-19 pandemic. It emphasizes the broader implications of research infrastructure beyond pandemic preparedness, such as addressing antibiotic resistance. The authors stress the need to develop a framework to fully account for the option value of research infrastructure to be better prepared for future emerging problems.  Implications: The implications of the paper are significant. It underscores the critical role of research infrastructure funding flexibility in enabling rapid and effective response to emerging health crises, such as the COVID-19 pandemic. The document also highlights the potential economic impact of research infrastructure, such as the development of the Oxford vaccine, on global and national economies, underscoring the significant value it can generate. Furthermore, it suggests that understanding the public's willingness to invest in resilience-building infrastructure is essential for policymakers to make informed decisions. |
| Ahmad 2022 | Knowledge mobilization | The unprecedented demand and urgency for research-based evidence during the pandemic highlighted the need for mechanisms to enable the use of evidence almost in real time. This necessitated investments in knowledge mobilization, implementation science, rapid learning systems, and scaling up data infrastructure.  The pandemic created an opportunity to reflect on simpler models for the uptake of research-based knowledge, as the scale of the problem created universal demand for solutions. The infrastructure, track record, and credibility of scientists contributed to enhanced knowledge mobilization, but there was also a unique opportunity to innovate and for people from outside the systems to help tackle multifaceted problems.  The pandemic facilitated a greater awareness of the uncertainty of knowledge and the consequences of actions, emphasizing the need for future knowledge mobilization processes to communicate the continuum of evidence and be transparent about uncertainty.  The experience of knowledge mobilization during the pandemic suggests the need for a more open approach to the full range of supply push, demand pull, and integrated approaches to routine decision making, so that systems for the generation and use of research knowledge acquire flexibility, memory, and resilience.  The pandemic highlighted the importance of maintaining successful infrastructures, networks, and registries outside the pandemic, as well as the need for adequate data on the complete patient pathway across health and social care.  The document also emphasizes the importance of investing in knowledge mobilization for other global health problems, such as antimicrobial resistance, and the need to incorporate advances in innovation diffusion and implementation to embed knowledge mobilization mechanisms into research effectively. Implications: None described. |
| Cochrane (2022) | Knowledge mobilization | Promote evidence, to counter mis/disinformation, and hold to account those intentionally creating and spreading mis/disinformation.  1. Enhancing Research Tools and Processes: There is a need to further develop or review research tools, processes, methods, and standards to effectively address the challenges posed by rapid-onset health emergencies. This includes investing in and utilizing new technologies to facilitate review processes, enhance transparency, and promote data sharing.  2. Investing in Science Communication: It is crucial to invest time and resources in science communication on an ongoing basis. This involves ensuring that people know where to access evidence, understanding effective formats and delivery methods, building trust in evidence during peacetime, enhancing information literacy, and fostering partnerships across disciplines and sectors to communicate uncertainty more effectively. |
| Harrison et al 2022 | Knowledge Mobilization | PCAB member feedback became repetitive highlighting fundamental deficiencies in researchers' understanding of the contextual factors/impact of COVID-19 on the patients and communities the studies were seeking to recruit from. Examples include a lack of culturally relevant and language concordant study materials related to recruitment, lack of attention to communities most impacted by COVID-19. **Implications:** May want to consider group based consultation rather than individual research team structure if feedback largely overlapped between sessions. |
| Office of the Chief Science Advisor of Canada, 2022 | Knowledge Mobilization | The pandemic emphasized the importance of science advice for emergency response and the need for a pre-existing system that is designed ahead of time, well understood by stakeholders, and can be quickly activated in an emergency. Establishing effective science advisory mechanisms is crucial to ensure that decision-makers have access to sound and timely science advice during emergencies. |
| Perillat 2021 | Knowledge Mobilization | Reporting of scientific findings needs to be considered and managed |
| Sohrabi 2021 | Knowledge Mobilization | The COVID-19 crisis has similarly imposed challenges on the long-­standing model of research publication, which has proven inadequate in situations requiring the rapid dissemination of data. Implications -In response to COVID-19, commercial publishers have tempo­rarily halted paywalls on coronavirus-related research to support research efforts and communications. Many traditional journals have also devised initiatives to help enhance the peer review process such as recruiting a pool of rapid review scientists and permitting extended or flexible revision timelines, all whilst ensuring that rigor and reproducibility remain paramount. Many researchers have also turned to non-peer reviewed preprint servers (e.g. medRxiv and bioRxiv servers) to facilitate the rapid dissemination of information  Conferences: The COVID-19 pandemic hit the business events industry with un­precedented force leading to the cancellation of many large-scale annual conferences and small society meetings. These decisions were enacted in light of the logistical difficulties that would be faced, including an inability to guarantee the safety of participants or to mitigate the negative effects of travel restrictions. Implications: Move to virtual conferences. This new format brings several unique opportunities but also numerous technical and organizational challenges. Virtual conferences allow attendance irrespective of travel restrictions and geographic travel constraints (reducing the associated carbon footprint), remove logistical and financial barriers to attendance, offer the advantage of accommodating thousands of attendees as opposed to hundreds, enable modera­tors to better control the flow of discussions, and are compatible with event marketing and sponsorship strategies |
| Scholten et al, 2020 | Leadership | The Canadian Institutes of Health Research (CIHR) and other national health bodies operated without a specific authority during public health crises. This lack of authority made it difficult to direct and coordinate research efforts, establish national databases and research platforms, ensure ethical and privacy safeguards, and provide swift resources for epidemic response research.  To address this issue, the National Advisory Committee recommended the establishment of a restructured national public health system with the authority to direct and coordinate research efforts during public health crises. This system would be responsible for establishing national databases and research platforms, ensuring ethical and privacy safeguards, and providing swift resources for epidemic response research.  Effective leadership is also critical in ensuring that research efforts are coordinated, and resources are allocated efficiently. The CIHR's response to the SARS outbreak highlighted the importance of having a clear and effective leadership structure in place to direct research efforts and allocate resources swiftly. This includes having dedicated funding streams and resources readily available for rapid deployment in response to emerging health crises.  Effective leadership and organization are critical in preparing for and responding to pandemics. A restructured national public health system with the authority to direct and coordinate research efforts, establish national databases and research platforms, ensure ethical and privacy safeguards, and provide swift resources for epidemic response research is essential. Additionally, having a clear and effective leadership structure in place to direct research efforts and allocate resources swiftly is crucial for effective pandemic preparedness and response. |
| Chen 2021 | Monitoring and evaluation | The importance of tracking and analyzing health research and development data in a regular and comprehensive manner to be able to highlight both progress and the lack thereof. |
| Demkowicz 2021 | Monitoring and evaluation | Call for Caution and Care: There is a recognition of the need for caution and care when reviewing the current evidence, especially when used to drive policy, and a call for research networks and organizations to minimize duplication and waste, facilitate collaborative and interdisciplinary work, and coordinate next step |
| DeVoe 2020 | Monitoring and evaluation | Real-time example of how the partnership between OSHIN PRBN and BRIDGE-C2 Center facilitated a rapid respond to evaluate the impact of COVID-19 pandemic on CHCs and their response. Implication: This example conveyed the benefit in pairing implementation science centers with PBRNs with expertise in D&I research to allow real-world, real-time evaluation to generate practice-based evidence |
| GloPID-R Secretariat (2023) | Monitoring and Evaluation | A challenge of applying research roadmaps during COVID-19 was the rapid evolution of research needs and generating evidence during a rapidly evolving pandemic. The potential implication is funding research that is no longer relevant. Implications: Some funders mitigated this risk by funding short-term grants with interim assessments for relevance and allowing grantees to pivot ongoing research. |
| Scholten et al, 2020 | Monitoring and Evaluation | Monitoring and evaluation lessons learned by health research systems from past pandemics, such as SARS and A/H1N1, emphasize the importance of timely data collection, robust surveillance systems, evaluation of interventions, capacity building, collaboration, and adaptive approaches. These lessons highlight the need for health research systems to prioritize real-time data collection, develop effective surveillance mechanisms, assess the impact of interventions, invest in capacity building, foster collaboration among stakeholders, and adopt adaptive monitoring and evaluation strategies. By incorporating these lessons, health research systems can enhance their preparedness and response capabilities for future pandemics, ultimately contributing to more effective and efficient public health responses. |
| Zakaria 2021 | Monitoring and evaluation | Three dominant paradigms continue to influence research impact assessment - 1) logic modelling, 2) systems-based approaches, and 3) realist evaluation. Implications: The authors argue that using a systems-based approach or realist evaluation approach may be most appropriate for capturing the impact of RI (above logic models - which are linear by nature). In addition, the emerging data ecosystem may provide an opportunity to support the identification and classification of platforms, supporting such an evaluation. |
| Zakaria 2021 | Monitoring and evaluation | There are various frameworks and approaches that can be used to assess RI but they are challenged with issues related to contribution/allocation, time lags, differences. Limitations exist for using number of citations, trainees trained. Implications: Focus needs to be paid to the following: 1. value of innovation, 2. team science, 3. reviewing RI through complex and systems-level change. Looking beyond traditional pipeline metrics to include platform-related metrics such as - emergence of responsible metrics and reducing waste in research agendas. Also - using qualitative metrics to support evaluation of RI. Also time lags - reframing the outcomes and outputs to align with RI and their life cycle. |
| Lane & Fauci, 2021 | Ongoing rigorous clinical research | Rigorous clinical research is important to maintain in the setting of an outbreak/epidemic. Implications: It was once widely held that the setting of an outbreak is not an appropriate venue for conducting rigorous clinical research because when people are dying, all possible therapies should be given a chance, rather than studied in rigorous ways. Such was the case during the 2014 and 2016 Ebola outbreak in West Africa, when many small studies were launched and few, if any, provided conclusive results. A thorough review of that situation by the U.S. National Academies of Sciences, Engineering, and Medicine concluded that randomized, controlled trials are the most reliable way to identify the relative benefits and risks of investigational products, and . . . every effort should be made to implement them during epidemics. These findings were endorsed by the global research community and led to an adequately powered, randomized, controlled trial during the 2018 to 2020 Ebola outbreak in the Democratic Republic of Congo that clearly identified two effective therapies. |
| Anderson et al 2022 | Organizational | Ensure all seven structural components are in place. Implications: "(a) organization and collaborations, (b) performance, (c) ethics and security, (d) scientific, (e) data, (f) information technology, and (g) patient outcomes." |
| Tille 2022 | OTHER | The significance of strengthening governance, including attention to open data sources, crisis and risk management, quality regulatory mechanisms, public sector management and communication, and policy coherence, coordination, and evaluation |
| Tille 2022 | OTHER | The effectiveness of transparent and effective public communication, policy evaluation, and dissemination of credible and consistent scientific advice by key government actors in building public trust and improving the effectiveness of governance responses to the pandemic |
| Hanney et al, 2020 | Policy development and implementation | Lessons learned from collaborative efforts in policy development and implementation underscore the significance of strong governance structures and regulatory frameworks. Implication: Governments and health authorities should prioritize the development and implementation of policies that support research for health, protect research participants, and ensure accountability within NHRS |
| Charalambakis 2021 | Prioritization | The importance of sustained investment and strategic planning: SRRs require sustained investment and strategic planning to enhance their capabilities and responsiveness, enabling them to address future scientific problems more effectively. Implications: The implication of this lesson is that funding agencies and policymakers need to prioritize collaborative research and centralize information about SRRs to improve research sustainability, broaden resource accessibility, and optimize the return of taxpayer investments |
| GloPID-R (2023) | Prioritization | Few developed research priorities during COVID-19 published updates to these priorities. E.g., the WHO updated and published their research roadmap once and engaged working groups for consultation and review. However, findings from the consultation were not made publicly available. Implications: The authors suggest there is a need to incorporate mechanisms for monitoring and evaluating progress on meeting research priorities including in research agendas, especially in fast-moving research environments/situations as with COVID-19. Planning should consider "living research roadmaps". |
| Hanney 2022 | Prioritization | Prioritization in the context of health research systems (HRSs) during the COVID-19 pandemic was a crucial aspect of the response to the public health crisis. The document emphasizes the significance of effective priority-setting within HRSs, which played a pivotal role in guiding research efforts, resource allocation, and ethical considerations. Here are some key points to expand on prioritization:  1. Rapid Response: The intense prioritization in various countries, especially in the early months of the pandemic, led to the rapid identification of research gaps and the allocation of resources to address critical areas such as vaccine development, therapeutic interventions, and public health measures.  2. Transparency and Equity: Effective prioritization mechanisms should be transparent and inclusive, incorporating an equity lens to ensure that the needs of diverse populations, including minority and underserved communities, are addressed. This approach helps in mitigating health disparities and ensuring that research efforts are aligned with the broader public health goals.  3. Coordination and Collaboration: Prioritization efforts often involved coordination and collaboration across research institutions, government agencies, and healthcare systems. This collaborative approach facilitated the pooling of resources, expertise, and data to address the most pressing research needs.  4. Ethical Considerations: Prioritization also encompassed ethical considerations, ensuring that research initiatives adhered to ethical standards and protocols. This included expedited ethics and protocol approvals to facilitate the timely implementation of research projects.  5. Impact Assessment: Effective prioritization should also incorporate routine impact assessments to evaluate the outcomes and effectiveness of research initiatives. This allows for the reallocation of resources based on the impact and success of ongoing projects.  6. Addressing Historical Factors: Prioritization efforts also recognized historical factors contributing to the vulnerability of certain populations during the pandemic, leading to the prioritization of research efforts aimed at addressing the concerns of these communities. |
| McMahon, Nidegal et al 2020 | Prioritization | The rapid-cycle priority identification process conducted by the CIHR's Institute of Health Services and Policy Research (IHSPR) demonstrated the importance of identifying specific research priorities to ensure a focused response to the most pressing research needs. |
| National Academies of Sciences, Engineering, and Medicine (2023) | Prioritization | Incentives for researchers and research institutions to participate in research-related response (recognition, funding, prestige) is misaligned with needs for a public health response (coordination and collaboration which may not result in valued incentives).  Implications: The authors suggest establishing clear, shared priorities. Flexibility in funding sources (compared with usual funding - which limits ability to respond in an emergency. |
| NIHR (2021) | Prioritization | The pandemic response has demonstrated the potential for accelerated research processes, particularly in the context of expedited research approval and delivery. Lessons learned include the need to scale up the use of digital enablers and develop new ways of realizing the potential of patient data to accelerate recruitment into clinical research studies. This includes supporting plans to develop a coordinated cross-agency response to a pandemic or other future health emergency. Implications: The implication of the lesson learned on accelerated research processes is the need to streamline and expedite research approval and delivery mechanisms. This includes leveraging digital enablers, optimizing the use of patient data for recruitment into clinical studies, and developing agile research protocols that can rapidly respond to emerging health emergencies. It also emphasizes the importance of cross-agency collaboration and coordination to facilitate a swift and effective research response to future health crises |
| OECD, 2023 (July 2023 No. 155) | Prioritization | The deployment of science capacity in a strategic and agile manner that is coordinated and timely. Continual and ongoing investments in science systems are required, as are expansions of established systems. Processes and mechanisms should be tested to allow for swift responses when needed |
| OECD, 2023 (July 2023 No. 155) | Prioritization | Open Science initiatives and the furthering of FAIR data principles is required, as is the development of new skills and methodologies. Transdisciplinary work is required with the inclusion of academic, government, and industry perspectives, as well as those provided by underrepresented groups. Implications: There is a need for integration, mobilization, and contextualization of data, knowledge, and expertise across disciplines and policy domains |
| OECD, 2023 (July 2023 No. 155) | Prioritization | Research priorities differ by geographic area, discipline, and sector, requiring trade-offs within limited funding budgets. Scientific research should cover a range of activities that result in outcomes that are societal, academic, and commercial in nature. Consideration should be given to the social, legal, and ethical issues that may arise when conducting research in emergent times, ensuring that there is trust at the "science-policy-society interface" |
| Office of the Chief Science Advisor of Canada, 2022 | Prioritization | The response to the pandemic highlighted the need for a deliberate effort to prioritize and fund research on data gaps during emergencies. Tight coordination and collaborations between the Chief Science Advisor and senior government officials are essential to ensure that funds are allocated in an agile manner and data are shared to support policy decisions |
| Perillat 2021 | Prioritization | Minimize future duplication of research works and the dispersion of scarce resources. |
| PHAC, 2010 | Prioritization | Rapid Conduct of Critical Research: the review emphasized the importance of considering options for the development of standardized mechanisms to facilitate the rapid conduct of critical research. This lesson underscores the need for pre-established processes to swiftly identify research opportunities, set priorities, and review proposals for funding during public health crises Implications: Strengths: This lesson reflects an understanding of the necessity for a structured and efficient approach to initiating critical research in response to emerging public health threats. It demonstrates a proactive stance toward ensuring that necessary research can be rapidly conducted to inform decision-making. Weaknesses: The lack of pre-established standardized mechanisms for rapid research prior to the pandemic may have hindered the ability to swiftly initiate critical research projects. This highlights a potential gap in the preparedness for rapid research responses to public health events. |
| Richardson 2021 | Prioritization | The article reflects on the experiences of the research team in conducting rapid, remote, and responsive research during the COVID-19 pandemic. Some of the lessons learned include the importance of working nimbly and being responsive to rapidly changing conditions. The authors emphasize the need for researchers to adapt to a changing social, political, and environmental context and to construct nimble responses to the research process itself. They also highlight the significance of developing good flexible working relationships across the research team and with research users, such as police officers, court managers, and victims of domestic abuse. Furthermore, the authors stress the need for early dissemination of results and recommendations to speed up the implementation process, as well as the importance of monitoring the implementation of recommendations to ensure their continuing relevance to research users. Overall, the article underscores the challenges and opportunities of conducting rapid response research and the importance of being adaptable and responsive in fast-changing research situations    **Implications:** The implications of prioritizing rapid response research methods and the dissemination of findings are significant. By prioritizing rapid research, the research team was able to produce impactful outcomes within a short timeframe, including academic publications, working papers, submissions to government inquiries, presentations to senior police leads, and policy recommendations. This prioritization allowed for the rapid dissemination of results and recommendations, enabling quick changes and improvements in practice, particularly in the context of the criminal justice system's response to domestic abuse during the COVID-19 pandemic. Additionally, the prioritization of rapid research methods underscores the need for researchers to be nimble, responsive, and adaptable to rapidly changing conditions, ultimately leading to more effective and timely impact with policymakers and practitioners. Therefore, the implications of prioritization are reflected in the ability to address urgent societal challenges and contribute to meaningful change within a constrained timeframe. |
| Sohrabi 2021 | Prioritization | The challenge of performing clinical research has been immense due to the suspension of many healthcare services. Implications: The impact of COVID-19 on clinical studies was immediate and will have long-standing consequences. Many clinical trials have been paused except for those focused on COVID-19. Enrolment into new studies has also been suspended due to the risk of spreading COVID-19. The impact has also been seen among trainees pursuing integrated clinical academic training pathways due to the shift in focus from research to patient care and the suspension of research activity. |
| Zakaria 2021 | Prioritization | Lack of clarity on what constitutes the start of the intervention (i.e., RI) and time points can be attributed to outcomes and the impacts. Especially related to the funding cycle of investment (if impacts can be measured during that lifecycle or need to be attributed longer term). Implications: Focusing on value metrics as described within Lesson 1; consideration for the development of hypothetical scenarios or projected impacts; supporting impact assessments and evaluations over a longer timeframe (10-15 years) |
| GloPID-R Secretariat (2023) | Priority Setting | Priority setting of research activities by organizations/jurisdictions had variable timeframes (days to several weeks). According to funders and researchers, delays in research roadmaps was a barrier to rapid initiation of research activities during COVID-19. Implications: The authors suggest establishing stakeholders groups ready to be consulted and engaged during similar outbreaks/pandemics. Additionally, using existing networks that can be rapidly mobilized in the event of an outbreak. Jurisdictions/organizations could also refer to the WHO R&D Blueprint mechanism. |
| McMahon, Nidegal et al 2020 | Rapid response | The response demonstrated the importance of quickly mobilizing resources to address the challenges posed by the pandemic. This included the rapid mobilization of funding, as well as the rapid identification of research priorities. |
| OECD, 2020 | Readiness and Responsiveness in Times of Crisis | While not explicitly stated, the paper implies the importance of ensuring the readiness and responsiveness of research infrastructures in times of crisis, such as the COVID-19 pandemic. This readiness requires effective management and optimization of infrastructures to address global challenges. The lesson on readiness and responsiveness in times of crisis implies that research infrastructure managers should ensure that research infrastructures are ready and responsive to global challenges, such as the COVID-19 pandemic. This requires effective management and optimization of research infrastructures to ensure their readiness and responsiveness in times of crisis. |
| Dyke & Mak, 2021 | Resilience | The pandemic has highlighted the need for adaptability and resilience within the health research system. Lessons learned include the importance of maintaining flexibility in research practices, funding mechanisms, and response strategies to address emerging needs and challenges |
| NIHR (2021) | Resilience | The pandemic response has emphasized the importance of building resilience and supporting the recovery of the health and social care system post-COVID-19. Lessons learned include the need to provide the research outputs that the NHS and wider health and social care system need to recover post-COVID-19, as well as to design and deliver services in a way that builds resilience. This includes ensuring that lessons learned from COVID-19 help to shape the future of research more generally, with a focus on greater integration of research within the NHS and wider health and social care services. Implications: The implication of the lesson learned on resilience and recovery is the need to prioritize research that supports the recovery of the health and social care system post-COVID-19. This includes directing resources towards studies that address the long-term impacts of the pandemic, as well as building capacity for future resilience through research-informed service design and delivery. Additionally, it underscores the importance of integrating research within the health and social care services to enhance preparedness for future challenges |
| daSilva et al 2022 | Resource Allocation & Capacity Building | Countries financed resources towards action that favored their national industry (in this case, vaccines and other high-technology products). Implications: This mechanism was recognized to be somewhat self-serving (serving the donor countries themselves by returning revenue to their industry). |
| Dyke & Mak, 2021 | Resource Allocation & Capacity Building | The COVID-19 pandemic has had significant impacts on health systems and funding for health research. Here's a breakdown of the key points:  1. Funding Gaps: The pandemic has created funding gaps for health research, particularly for rapid research on COVID-19 and ways to mitigate its effects. Additionally, there has been a gap in financial support for researchers whose work and livelihoods were halted, cut back, or delayed due to COVID-19. Health charities have also been significantly impacted, with reduced donations and a decrease in funding for non-COVID-19 research.  2. Potential Funding Cuts: There is also concern about the potential for substantial funding cuts as part of post-COVID fiscal restraint, despite the evident value of supporting health research in Canada. This could have long-term impacts on the health research system and the ability to address future public health emergencies.  3. Impact on Non-COVID-19 Research: The pandemic has also had an impact on non-COVID-19 research, with funding redirected to COVID-19 research and potential delays in ongoing research projects. This could have long-term impacts on health outcomes for many years.  Understanding these impacts is crucial for developing strategies to support the resilience of the health research system. This may include advocating for integrated and shared data, ensuring consistent, secure, and reliable access to quality data, and promoting adherence to Indigenous research principles in relevant funding calls and reviews. Additionally, addressing funding gaps and potential funding cuts will be essential to support ongoing health research efforts and ensure that the health research system remains resilient in the face of future public health emergencies. |
| Hanney et al, 2020 | Resource Allocation & Capacity Building | Capacity-building initiatives, particularly in low- and middle-income countries, have demonstrated the value of training in research methodology, ethics, and priority-setting. Implication: Investing in research capacity-building is essential for enhancing the skills and capabilities of researchers, particularly in resource-constrained settings, to conduct relevant and impactful research |
| Hanney et al, 2020 | Resource Allocation & Capacity Building | The challenge of securing sufficient funding for health research and supporting activities is a major obstacle globally. Lessons learned emphasize the need for countries to prioritize and allocate dedicated national budgets for health research. Implication: Governments and policymakers should recognize the importance of sustained and adequate funding for health research to address critical health challenges and promote evidence-based policy and practice. |
| Harrison et al 2022 | Resource Allocation & Capacity Building | Overall funding for community-engaged COVID-19 research is lacking. **Implications:** A need exists for additional funding focused on community-engaged research to support the implementation of many of these lessons. |
| Kentikelenis 2023 | Resource Allocation & Capacity Building | Increases in funding calls related to HPSR were observed until 2019, but this situation reversed in 2020, likely reflecting the redirection of resources to rapid assessments of the impacts of the COVID-19 pandemic.  **Implications:** The pandemic has highlighted why investing in HPSR is more important than ever to enable the delivery of effective health interventions, so this decrease is critical to understand and address. |
| PHAC, 2010 | Resource Allocation & Capacity Building | Contingency Funds for Rapid Research Projects: The review suggested the consideration of contingency funds to initiate rapid research projects. This lesson underscores the importance of having dedicated resources available to support urgent research needs during public health emergencies. Implications: The review highlight the importance of flexibility in resource allocation to address evolving research needs during a public health crisis. The ability to adapt resource allocation based on shifting research priorities and emerging challenges is critical for an effective response |
| Scholten et al, 2020 | Resource Allocation & Capacity | The Canadian Institutes of Health Research (CIHR) and other national health bodies faced limitations in the linkage of laboratory research at a national level, which was primarily confined to local and provincial levels. To address this, it was recognized that establishing national databases and research platforms is essential for improving research capacity. These platforms would allow for the effective collection, sharing, and analysis of clinical, epidemiologic, laboratory, and pathological data necessary to fully understand diseases such as SARS. By creating a national SARS database and similar platforms for future pandemics, the national collaborative effort against infectious diseases can be substantially improved. Furthermore, the establishment of the Canadian SARS Research Consortium (CSRS) by the CIHR was a step towards enhancing research capacity. The CSRS focused on bringing together funding partners, eliminating duplicative efforts, and developing more cooperative interaction between different research groups. It unraveled diagnostic assays for SARS, conducted epidemiological modeling of the disease outbreak, and examined strategies for future actions. The findings of the CSRS provided a preliminary infrastructure that could serve as a model for future outbreaks of pandemics in Canada. Enhancing research capacity for future pandemics involves establishing national databases and research platforms to facilitate the collection, sharing, and analysis of essential data. Additionally, fostering cooperative interaction between different research groups and establishing infrastructure for collaborative research efforts are crucial steps in improving research capacity for pandemic preparedness and response. |
| Sohrabi 2021 | Resource Allocation & Capacity Building | The COVID-19 lockdown has led to monumental implications for charities and funding for scientific research. Implications: The increasingly likely prospect of a long-term economic downturn could lead to longer-term  impacts on development budgets, funding from direct donations, as well as government and charitable grants. |
| Bandera 2020 | Strategic planning Strategic plan | Creation of COVID-19 Registry and Biobank: The establishment of the COVID-19 Network and its associated biobanking activities allowed for the collection of biospecimens for basic science and translational research projects, contributing to a better understanding of the disease. Facilitation of Research Activities: The COVID-19 Network's tools and strategies, such as strong leadership, IT resources, coordination between research units, involvement of data managers and technical personnel, and collaboration with other research institutes and clinical centers, have facilitated research activities and contributed to the production of numerous research articles on COVID-19. |
| Blomberg 2020 | Strategic planning Strategic plan | Providing open-source tools, workflows and computational resources to support reusable and reproducible  computational analysis of COVID-19 data |
| Charalambakis 2021 | Strategic planning Strategic plan | The need for a national strategy: A national strategy is needed to improve inter-agency coordination of SRR programs, address challenges and new opportunities, and ensure long-term research sustainability. Implication: The implication of this lesson is that policymakers and research stakeholders need to join forces and concentrate on identifying knowledge and policy gaps that impede the full potential of SRRs and addressing these missed opportunities by developing mechanisms that encourage the integration of SRRs across scientific disciplines |
| Hanney 2022 | Strategic planning | Health research strategies are critical for the effective functioning of health research systems (HRSs) during a global health crisis such as the COVID-19 pandemic. The document highlights the importance of pre-existing comprehensive health research strategies and vision in enhancing the effectiveness of specific steps and opportunities for producing research to improve policies, practice, and health.  key components of health research strategies that are essential for effective pandemic response:  Coordination: Health research strategies should prioritize the development of coordination mechanisms and strategies to enhance research preparedness for future pandemics.  Prioritization: Health research strategies should include transparent mechanisms, including using an equity lens, for wide/public engagement in priority-setting.  Evaluation: Health research strategies should incorporate routine impact assessments at the project and system level to understand where and how impact was maximized.  Financing: Health research strategies should work to determine the benefits of increased funding for COVID-19 research and the damage done by the loss of funding for non-COVID-19 research.  Translation: Health research strategies should attempt to accelerate the translation of early research into new drugs and vaccines. |
| McMahon, Nidegal et al 2020 | Strategic planning | The response highlighted the importance of long-term planning to address the broader health system implications of the pandemic beyond immediate research needs. This includes the need to consider the long-term consequences of the pandemic on health, health equity, and health system outcomes, as well as the post-COVID-19 health, social, and economic policies that are created. |
| NIHR (2021) | Strategic plan | The pandemic response has highlighted the importance of being prepared for future pandemics. Lessons learned include the need to ensure that the entire research system is aligned and ready to develop, fund, and deliver pandemic research studies. This includes incentivizing greater use of platform studies to rapidly evaluate the effectiveness of multiple interventions to tackle new infectious diseases and supporting the work of the UK Health Security Agency through close partnership working. Implications: The implication of the lesson learned on preparedness for future pandemics is the need for ongoing investment in pandemic preparedness, including research infrastructure, funding mechanisms, and collaborative networks. This involves ensuring that the research system remains agile and ready to respond to emerging infectious diseases, thereby contributing to global health security and resilience |
| OECD, 2020 | Strategic management | National strategies provide a framework for the development and management of research infrastructures. They help to align research infrastructure investments with national research priorities and ensure that resources are allocated effectively. Implications: The lesson on user-base optimization implies that research infrastructure managers should consider the diverse and numerous potential users of research infrastructures, particularly as data becomes more complex and varied. This requires the development of policies and mechanisms to optimize user access and ensure that research infrastructures are accessible to underrepresented groups. |
| Hanney et al, 2020 | Strengthening NHRS functions | Efforts to enhance the functions of NHRS, including stewardship and governance, financing, capacity-building, and producing and using research, have highlighted the importance of comprehensive and coordinated approaches. Implication: NHRS should focus on strengthening all functions in a coherent and integrated manner to ensure effective governance, sustainable financing, and impactful research outcomes |
| Whitehouse 2022 | Sustainability | The authors note that for the research advancements and changes that were made during the COVID-19 pandemic to be longstanding, there will need to be a commitment towards resources, such as funding, time, and space, and towards the recognition of an "embedded approach to research as a core care activity" in organizations. |
| OECD, 2023a | Methods | 1. Urgent Need for Rapid Knowledge Production: The pandemic highlighted the critical importance of rapidly producing new scientific knowledge and tools to address complex societal challenges. The ability of science systems to respond quickly and effectively to crises was put to the test, emphasizing the need for agility and adaptability in research processes.  2. Accelerated Access to Research Results: Institutions and publishers were encouraged to develop expedited, transparent, and trusted peer-review processes to accelerate access to important research results. Utilizing preprint platforms to make preliminary outcomes openly accessible was also recommended, with a focus on ensuring replicability through the availability of supporting data alongside preprints.  3. Ethical Data Collection and Use: Policymakers, funders, and research institutions were advised to facilitate the rapid, ethical, and secure collection, stewardship, and use of data during crisis response. Promoting the universal adoption of informed consent procedures and sharing agreements to enable ethical downstream sharing, reuse, and preservation of data was emphasized, particularly in the context of social sciences data.  4. Transparency and Accountability in Research Practices: During crisis response, it was crucial to maintain full transparency and accountability in extraordinary measures adopted for data collection and analysis. Engaging a diverse range of stakeholders, including scientists, privacy enforcement authorities, and civilians, ex ante ensured that actions were warranted, proportionate, and aligned with societal values. |
| OECD, 2020 | User-base optimization | The paper emphasizes the significance of optimizing the user-base of national research infrastructures. It underscores the need to consider the diverse and numerous potential users of these infrastructures, particularly as data becomes more complex and varied. |

### APPENDIX E: Recommendations for HRS to better respond to next pandemic

| **Author, year** | **Category** | **Recommendation Description** | **Implications/Outcomes (when described)** |
| --- | --- | --- | --- |
| Ayano Ogawa 2019 | Collaboration | There is a need for improved global coordination and accountability for country preparedness. Health research systems should work towards strengthening the World Health Organization (WHO), the United Nations (UN), and broader global health or humanitarian systems | |
| Becerra-Posada 2021 | Collaboration | Partnerships with private institutions: LAC countries could consider partnerships with private institutions, such as charitable foundations, to diversify funding sources for the development of HPSR and to address the challenges posed by the pandemic. | |
| Cochrane Convenes 2022 | Collaboration | Enhancing research tools, methods, and standards. Utilizing new technology for efficient review processes (e.g., AI, citizen science, study repositories). Exploring agile editorial formats (e.g., rapid reviews, preprints). Investing in ongoing science communication efforts to improve access, trust, and literacy. Fostering interdisciplinary partnerships to address needs and communicate uncertainty effectively. Additionally, advocating for transparency, reducing research waste, building capacity in lower-income countries, and engaging directly with evidence users to convey evolving uncertainties. | |
| Cochrane Convenes 2022 | Collaboration | Enhance decision-making: Collaborate with national and international decision-makers to align on global health emergency needs and research contributions.    Streamline research prioritization: Foster consensus on global research needs and coordination strategies, exploring options for a cohesive global evidence system.    Strategic funding for global needs: Invest in research and communication alongside short-term projects, ensuring equitable support for national and international research priorities. Increase financial backing for evidence generation, communication, and infrastructure in low- and middle-income countries. | The Covid-19 pandemic has shown why investing in HPSR is more important than ever to  enable the delivery of effective health interventions and avoid costly implementation failures. |
| Cochrane Convenes 2022 | Collaboration | Listen actively to diverse audiences and understand their communication needs. Engage with professionals from various disciplines to refine recommendations and action plans. Top-line recommendations include researching effective communication strategies, building trust through collaboration, increasing transparency in decision-making processes, and raising awareness of evolving evidence in emergencies. Consider accreditation for reliable sources, engage information scientists, use non-traditional formats, and form multidisciplinary coalitions to combat misinformation. | |
| Demkowicz 2021 | Collaboration | Efforts to further develop and expand cross-institutional and interdisciplinary networks should be undertaken to support greater collaboration and more efficient resource deployment, with funders considering how their calls can directly encourage cross-institutional collaboration | |
| Devoe 2020 | Collaboration | Collaborate with Implementation Science Centers: Partner with implementation science centers to accelerate research efforts and expand scientific work in response to public health crises. This collaboration can help in identifying effective implementation strategies and training needs for healthcare providers | |
| Hanney et al., 2020 | Collaboration | Engage stakeholders in the development and operation of the National Health Research System Strategy, including setting priorities for research, research processes, and translation. | |
| Hinton et al (2021) | Collaboration | In their original pre-pandemic recommendations, the authors noted that multisectoral collaboration requires engagement through multiple stakeholders whose involvement may differ throughout the course of the study. Therefore, the authors emphasize a sectoral analysis to be completed to assess the benefits of collaboration and each sector's potential contributions. In the context of COVID-19, the authors acknowledge that task forces that were multisectoral were often set up by governments and international organizations. The authors highlight the need for undertaking sectoral analyses to improve collaboration and determine which original structures can be leveraged. The authors further emphasize the need for identifying and including "boundary spanning stakeholders" in the collaborations due to the quick turnaround required. | |
| NIHR 2021 | Collaboration | The recommendation underscores the need to strengthen the engagement of people and communities in research by better using digital technologies and reaching out more effectively to communities under-served by research. This involves building on heightened awareness of research because of COVID-19 to strengthen engagement efforts and ensure that the perspectives and lived experiences of individuals in relation to their health and care are heard and acted upon | By strengthening engagement with people and communities, the implication is that the health research system can enhance inclusivity, responsiveness, and relevance in addressing future health emergencies. This includes leveraging digital technologies to broaden participation in research, addressing disparities in research engagement, and ensuring that research efforts are informed by and responsive to the diverse needs and perspectives of communities. |
| Norton et al (2020) | Collaboration | Diverse Expertise and Resources: Multisectoral collaboration involves bringing together stakeholders from various sectors, including government agencies, research institutions, non-governmental organizations, healthcare providers, industry partners, and community representatives. Each sector contributes unique expertise, resources, and perspectives to address complex public health challenges.  Comprehensive Approach: By engaging multiple sectors in the research process, a more comprehensive and holistic approach to addressing pandemics can be achieved. Collaboration allows for a broader range of issues to be considered, from basic science research to policy development and implementation.  Shared Goals and Objectives: Multisectoral collaboration helps align stakeholders around common goals and objectives related to pandemic preparedness, response, and recovery. By working together towards shared outcomes, stakeholders can leverage their collective strengths and resources to achieve greater impact.  Coordination and Communication: Effective collaboration requires strong coordination mechanisms and clear communication channels among stakeholders. Regular meetings, information sharing, and feedback loops help ensure that research efforts are coordinated, resources are optimized, and progress is monitored.  Capacity Building and Knowledge Sharing: Multisectoral collaboration provides opportunities for capacity building and knowledge sharing across different sectors. By sharing expertise, best practices, and lessons learned, stakeholders can enhance their skills, expand their networks, and improve the quality of research and interventions.  Policy Development and Implementation: Collaboration among sectors can facilitate the development and implementation of evidence-based policies and interventions to address public health challenges. By involving policymakers, researchers, and practitioners in the research process, findings can be translated into actionable strategies that benefit communities.  Community Engagement: Multisectoral collaboration can also involve engaging with communities and stakeholders at the grassroots level. By involving community members in research design, implementation, and dissemination, research efforts can be more responsive to local needs and priorities.  Global Impact: Multisectoral collaboration on a global scale can lead to more coordinated and effective responses to pandemics that transcend national boundaries. By working together across sectors and countries, stakeholders can address shared challenges and promote global health security. | |
| OECD 2020 | Collaboration | Exchange of Good Practices: Research infrastructure managers should engage in collaboration and mutual learning to identify cost-sharing and cost-saving opportunities. This can involve the development of networks and partnerships among research infrastructure managers to share knowledge and expertise | |
| OECD 2023c | Collaboration | Foster science-industry collaborations for rapid crisis response. Strengthen partnerships between academia and the private sector. Experiment with agile funding mechanisms to address conflicting priorities and incentives. | |
| Office of the Chief Science Advisor of Canada | Collaboration | The importance of international scientific cooperation in addressing global health emergencies. Collaborating with other countries and sharing scientific knowledge and best practices can enhance the global response to pandemics and other emergencies. This implies the need for continued collaboration and information sharing on a global scale | The lessons learned emphasize the importance of international scientific cooperation in addressing global health emergencies. Collaborating with other countries and sharing scientific knowledge and best practices can enhance the global response to pandemics and other emergencies. This implies the need for continued collaboration and information sharing on a global scale |
| Rosenthal 2023 | Collaboration | Our use of this iterative and participatory process helped to ensure staff buy-in throughout the entire process and it will facilitate institute-wide support over the next 3 years. This was primarily conducted through regular meetings with the Research Agenda Committee, comprising staff from diverse research, programmatic, and practice backgrounds who were employed at varying levels of the organization.  Group consensus whenever possible, can be used particularly in the development of the prioritization criteria and initial prioritization of research questions. | There is optimism that staff involvement throughout the Research Agenda development process and the programmatic selection of questions of interest will contribute to the success of the planned research. However, the adaptive nature of this process has continued through the process of operationalizing questions and removing those where the landscape has shifted such that there would no longer be practical results. |
| Sohrabi 2021 | Collaboration | International research efforts, built on collaboration,  have allowed for significant breakthroughs to be made regarding our understanding of the pandemic | The CKSF would foster collaboration across the system and urgent, multi and interdisciplinary research. It would also improve support for talent through better coordination and programming. |
| SRDC 2021 | Collaboration | There is a need to develop a broader understanding of what research is being funded and identify gaps in research funding is crucial to ensure that research efforts are aligned with community needs and priorities. This includes developing a joint research funding plan to address these gaps, including alternative funding models. Regular mapping and coordination of research funding during emergencies and non-emergencies are essential to ensure that research efforts are coordinated, and resources are maximized.  Regular mapping and coordination of research funding during emergencies and non-emergencies can help identify gaps in research funding and ensure that research efforts are aligned with community needs and priorities. This can involve mapping research funding by location, area, populations, pillar, COVID-19 related and non-COVID related research, and researcher career stage. From this, the health research funding partners should develop a joint research funding plan to address these gaps, including developing alternative funding models.  Developing a joint research funding plan can help ensure that research efforts are coordinated, and resources are maximized. This can involve identifying research trends, discoveries, or new priorities, stimulating multidisciplinary research partnerships, and identifying who might fill any funding gaps. Regular mapping and coordination of research funding during emergencies and non-emergencies can help ensure that research efforts are aligned with community needs and priorities, and that resources are maximized to address the challenges posed by public health emergencies. | |
| WHO 2020 | Collaboration | Incorporate features such as conducting research to meet the needs of the health-care system, collaboration with potential users, and structures encouraging research use. This could boost research use and improve the relevance and impact of research outcomes | |
| Coller et al., 2021 | CTSA Hub - virtual operations | Strengthen ability to support virtual research operations. Create a virtual clinical research website with resources and policies, including up-to-date information from US FDA and US Department of Health and Human Services (HHS) | |
| Tsang et al., 2022 | Culture change | Culture change wherein frontline clinicians and research personnel are engaged and supported to participate in research. Federal, provincial, and territorial governments must recognize central role of research in a learning healthcare system. | Government support is necessary to build and maintain a research infrastructure that can inform best care. Pragmatic, patient-centered research will reduce health system costs. |
| OECD 2023a | Data Access | Establish and utilize inclusive and ethical data infrastructures. Address barriers hindering LMICs' participation in global science efforts. Advance data management for FAIR principles and open access. Navigate conflicting interests in knowledge accessibility. Enable swift and secure data collection and utilization during crises while enhancing research access. | |
| OECD 2023a | Data driven | Emphasize Micro-Level Analysis: The article suggests the importance of complementing traditional statistical analysis of R&D funding with micro-level analysis of project data. This recommendation implies the value of delving into granular project-level details to gain deeper insights into the allocation and impact of research funding. Decision-makers may consider incorporating micro-level analysis into their R&D funding evaluation processes to enhance understanding and decision-making. | |
| Becerra-Posada 2021 | Data Governance | Implementing open-data policies at the governmental level could facilitate access to research financing data, allowing for greater transparency and accountability in the allocation of resources for health research, including responses to the pandemic. | |
| Mishra 2023 | Data Governance | The establishment of data governance across diverse communities and settings is identified as a critical factor for conducting research for a tailored public health response. The article emphasizes the importance of building community trust and developing data governance models ahead of a public health emergency. It specifically highlights the exemplar of Indigenous data systems and governance, emphasizing that Indigenous self-determination and rights should inform Indigenous governance of Indigenous data and its use by Indigenous leadership. | |
| OECD 2023a | Data Governance | Foster Data-Driven Decision-Making: The article underscores the potential of leveraging data-driven approaches to form R&D funding decisions. This recommendation implies the need for funders to prioritize the use of quantitative and qualitative data, advanced analytics, and modeling techniques to optimize resource allocation, identify research priorities, and enhance the effectiveness of R&D funding strategies. | |
| Adam 2023 | Equity | Reducing Disparities in Resource Allocation: The commentary emphasizes the need to reduce disparities in resource allocation for health research between high-income and low-income countries. Currently, there is a significant gap in funding for health research, with low-income countries receiving a very small proportion of grant funding compared to high-income countries. Addressing these disparities is crucial for ensuring that all populations have access to the resources needed to address their health challenges  Increasing Funding for Research in Low- and Middle-Income Countries: To improve preparedness for future pandemics, it is essential to increase funding for research in low- and middle-income countries. These regions often face a higher burden of diseases but have limited resources for research and development. By providing more funding to support research initiatives in these countries, policymakers can help address the health needs of underserved populations and strengthen global health security  Promoting Equity in Health Research: Efforts to address inequities in resource allocation should focus on promoting equity in health research. This includes ensuring that funding is directed towards priority areas that have the greatest impact on public health, regardless of the income level of the country. By promoting equity in health research, stakeholders can work towards narrowing the gap in health outcomes between different populations and improving overall health equity worldwide | |
| Devoe 2020 | Equity | Develop Interventions for Underserved Populations: Focus on developing interventions that aim to improve the delivery of primary care, especially for underserved populations. Understanding the impacts of practice changes on the quality of services can guide the development of targeted interventions | |
| SRDC 2021 | Equity | The recommendation to support equity, diversity, and inclusion in health research is important to ensure that research efforts are inclusive and representative of diverse populations. This includes recognizing and addressing multiple sources of dis/advantage, such as race, gender, socioeconomic status, and disability, among others.  An intersectional approach to equity, diversity, and inclusion recognizes that individuals may experience multiple forms of dis/advantage simultaneously, and that these experiences can interact in complex ways. For example, a woman of color may face unique challenges and barriers in accessing health care and participating in research that are different from those faced by a white woman or a man of color. By recognizing and addressing these intersectional experiences, health research can be more inclusive and representative of diverse populations.  Greater efforts are needed to support equity, diversity, and inclusion in health research, including the use of strategies to address inequities in the research system during COVID-19 . This can involve measures such as equalizing funding opportunities for underrepresented groups, encouraging inclusion of diverse groups on research teams, and applying an intersectional lens to equalize funding opportunities .  Monitoring the effects of these efforts is also important to ensure that they are effective in promoting equity, diversity, and inclusion in health research. This can involve tracking the representation of diverse groups in research studies, evaluating the impact of interventions to promote equity, diversity, and inclusion, and regularly reviewing and updating policies and practices to ensure that they are inclusive and representative of diverse populations. | |
| Bouchard et al., 2023 | Governance | The panel recommends creating a new, complementary governance mechanism (in addition to the existing CRCC), with a clear division of responsibilities between it and the councils. The panel calls this new mechanism the Canadian Knowledge and Science Foundation (CKSF). Would be designed to better support coordination and encourage urgent, international, multi and interdisciplinary and mission-driven research. It would build on the CRCC - which would be discontinued. | |
| Collins 2023 | Governance | Involve Regulators and Establish Clinical Guidelines: The recommendation is to closely involve regulators to avoid missteps that can cost months and establish a rigorous, trustworthy clinical guidelines system to translate research findings into clinical practice in real-time. | |
| Hanney 2022 | Governance | Enhanced Coordination: Improve coordination within HRSs, including coordination mechanisms for research preparedness and response to pandemics.   Integration with Healthcare Systems: Foster integration between health research and healthcare systems through comprehensive health research strategies.   Comprehensive Health Research Strategies: Develop and implement comprehensive health research strategies involving broad stakeholder engagement to maximize input and collaboration.   Governance and Decision-Making: Establish governance structures that facilitate rapid decision-making and resource allocation during a public health crisis.   Mitigating External Factors: Acknowledge and account for social and political factors outside the boundaries of HRSs that can impact pandemic response. | Enhanced coordination facilitates the efficient mobilization of resources, expertise, and data during a public health crisis. It allows for the rapid identification of research priorities, streamlined decision-making, and effective collaboration among research institutions, government agencies, and healthcare systems.  Integrating research directly into healthcare settings enables the seamless translation of research findings into clinical practice. This integration facilitates the rapid adoption of evidence-based interventions, treatments, and public health measures, leading to improved patient outcomes and population health during a pandemic.  Involving diverse stakeholders, including researchers, healthcare providers, policymakers, and community representatives, in the development of health research strategies ensures that the response to future pandemics is inclusive, equitable, and aligned with the needs of diverse populations. This collaborative approach fosters a more holistic and effective pandemic response.  Effective governance structures enable agile responses to emerging research needs, ethical considerations, and public health priorities. This ensures that research initiatives are aligned with the broader public health goals and are responsive to evolving challenges during a pandemic.  Recognizing and mitigating external factors, such as political influences and societal dynamics, can help HRSs adapt to unforeseen challenges and navigate complex environments during a public health crisis. This proactive approach allows for more resilient and adaptable pandemic response strategies. |
| Hanney 2022 | Governance | The recommendation to improve governance and decision-making within health research systems (HRSs) involves enhancing coordination and establishing a clear vision, establishing effective priority-setting mechanisms, streamlining ethics and evaluation processes, ensuring accountable financing and resource allocation, and mitigating the negative impacts of the pandemic.  These efforts are essential for strengthening the capacity of HRSs to respond to future pandemics, ensuring a more strategic, inclusive, and resilient public health response. | By improving coordination, prioritization, and resource allocation, HRSs can align research efforts with public health priorities, mobilize resources effectively, and respond rapidly to emerging challenges during a pandemic, fostering a more cohesive and impactful pandemic response. |
| NHS 2021 | Governance Structures | Ethical and Transparent Conduct of Research: The document emphasizes the importance of governance in ensuring that research is conducted ethically and transparently. This involves ensuring that research is conducted in accordance with ethical principles and guidelines, and that research findings are disseminated in a transparent and accessible manner.  2. Effective Management of Research Resources: The document highlights the importance of effective management and governance structures to ensure that research resources are used efficiently and effectively. This involves ensuring that research funding is allocated in a fair and transparent manner, and that research studies are conducted in accordance with best practices and standards.  3. Collaboration and Partnership: The document emphasizes the importance of collaboration and partnership in governance structures, including the involvement of patients, the public, and other stakeholders in the governance of research. This involves ensuring that research is conducted in partnership with patients and the public, and that research priorities are aligned with the needs and priorities of the wider community.  4. Continuous Improvement: The document highlights the importance of continuous improvement in governance structures, including the need for ongoing evaluation and monitoring of research activities to ensure that they are conducted in accordance with best practices and standards. | |
| OECD 2020 | Governance | Research infrastructure managers and policymakers should establish clear governance structures to define roles and responsibilities, decision-making processes, and accountability mechanisms. This can help ensure effective management and operation of research infrastructures. | |
| Singh et al (2020) | Governance | The authors provide guidelines for non-COVID-19 related studies that were ongoing during the start of the pandemic. These include the development of procedures for study participants who develop COVID-19 infection, the use of alternate communication methods including virtual options, supplying personal protective equipment, using appropriate statistical methods to account for study recruitment and follow-up issues during the pandemic, establishing contingency plans to ensure an adequate supply of study supplies, developing strategies to deal with the socioeconomic situations of participants (e.g., food insecurity), and the sharing of information on pandemic-related delays with study funders, research ethics boards, and monitoring boards | The implications highlight the need for a deliberate effort to prioritize and fund research on data gaps during emergencies. Tight coordination and collaborations between the Chief Science Advisor and senior government officials are essential to ensure that funds are allocated in an agile manner and data are shared to support policy decisions. Prioritizing research can help address critical data gaps and inform evidence-based policy decisions during emergencies |
| Tille 2022 | Governance Structures | It highlights the importance of strengthening governance during the pandemic, including attention to open data sources, crisis and risk management, quality regulatory mechanisms, public sector management and communication, and policy coherence, coordination, and evaluation . The article also notes that an increase in trust in government and health officials led to a greater acceptance of government-mandated measures and less politicization of the pandemic and its societal impacts . The article suggests that transparent and effective public communication, policy evaluation, and dissemination of credible and consistent scientific advice by key government actors were important in building public trust and improving the effectiveness of governance responses to the pandemic | |
| WHO 2020 | Governance | Develop and implement policies that support research for health, protect research participants, and ensure accountability within NHRS. This includes establishing laws regulating research for health, national strategic health research plans, and health research management forums | |
| Demkowicz 2021 | Infrastructure | There is a need to develop the research infrastructure to better facilitate responses to future crises, including the development of longer-term mechanisms to allow the mental health research community to act proactively in the face of emerging crises | |
| OECD 2023c | Research Infrastructures | Recognize and support research infrastructures (RIs) for crisis preparedness and resilience. Invest in human and technological capacity for crisis response. Empower RIs with technological and governance innovations for resilience. Foster inclusive national and regional RI ecosystems to tackle grand challenges. Coordinate national, regional, and international RIs for global science and regional needs. | |
| Office of the Chief Science Advisor of Canada | Infrastructure | Invest in science infrastructure to support research and innovation in areas such as genomics, data analytics, and artificial intelligence. This investment can help to accelerate the discovery of new therapies and vaccines and improve the response to future public health emergencies | |
| Tsang et al., 2022 | Infrastructure | Academic hospitals should be engaged in remodelling research infrastructure, via a hub-and-spoke organizational design that connects academic and community hospitals, like the Canadian Cancer Clinical Trial Network (3CTN.ca). Academic centres can help community hospitals by providing ethics expertise, training research coordinators, pharmacy and lab best practices. | |
| Ahmad 2022 | Knowledge Mobilization | Investment in Knowledge Mobilization: The document emphasizes the importance of investing in knowledge mobilization for other global health problems, including the threat of antimicrobial resistance. It suggests substantial strengthening of knowledge mobilization for global health challenges and the incorporation of advances in innovation diffusion and implementation to embed knowledge mobilization mechanisms into research effectively.  Maintenance of Successful Infrastructures: The document highlights the need to maintain successful infrastructures, networks, and registries outside the pandemic. It emphasizes the importance of sustaining existing infrastructure, such as clinical research networks, health protection research units, and patient safety translation centers, to support a ready response to smaller and larger scale threats.  Data Availability and Integration: The document stresses the importance of ensuring adequate data availability on the complete patient pathway across health and social care. It also recommends a more permanent shift in the early posting of research protocols and results for other less immediate but serious global threats to reduce duplication of effort and focus on implementation studies.  Trust and Public Commitment: The document suggests fostering trust and strong public commitment early to participate and contribute data. It also emphasizes the need to build on the positive cultural change and choreograph informed citizen science to shape and raise questions for research.  Technology for Mass Public Participation: The document highlights the potential of technology, such as smartphone applications, for capturing patient-generated data and enabling mass public participation in research. It recommends the potential use of technology for communicable and non-communicable conditions and as an early warning system. | |
| Ayano Ogawa 2019 | Knowledge mobilization | Health research systems should prioritize knowledge sharing and research to enhance understanding of the emergence, prevention, detection, and control of pandemic influenza viruses. This includes investing in One Health research, enhancing collaboration between human and animal health sectors, and developing more comprehensive surveillance for infection and disease in occupational groups that work closely with animals | |
| Bouchard et al., 2023 | Knowledge Mobilization | The panel recommends the proposed Canadian Knowledge and Science Foundation have clear linkages to future work of the Canadian Innovation and Investment Agency and the governments business-facing innovation and commercialization. | Innovation and commercialization are important for translating research results to further benefit Canadians. |
| Coller et al., 2021 | Knowledge Mobilization | Assist researchers in preparing reports for publications. Encourage researchers to accept invitations from journals to review manuscripts as rapidly as possible. | |
| Harrison 2022 | Knowledge Mobilization | Better training of both trainees, early career researchers, and established researchers on best practices for conversations with patient and community consultants. | This might include setting and evaluating investigator competencies for community-engaged research and having researchers watch a short video module on ground rules and best practices prior to attending the consultation session. |
| Kayano 2021 | Knowledge mobilization | - Establish WHO health EDRM RN for global research collaboration and develop a research agenda. - Launch WHO health EDRM knowledge hub and expedite ethical approval processes. - Standardize impact evaluation methods, streamline funding structures, and address complex health risk conceptualization. - Enhance value in health EDRM research while avoiding duplication and create an online platform for information sharing. - Involve stakeholders in agenda development, prioritize research themes, and build consensus on priorities. - Coordinate closely with stakeholders, form a working group for priority identification, and align efforts with global health frameworks. | Important to minimize damage to the public's trust in science and improve understanding as well as public health. |
| Perillat 2021 | Knowledge Mobilization | Reporting of scientific findings needs to be considered and managed. The publication of findings during the COVID-19 pandemic highlights the need to follow the five principles outlined by Smith, Upshur and Emanuel: ensuring scientific accuracy, social value (data must be released and (in)validated by the scientific community), protection of research participants, transparency and accountability on the part of journal editors. | |
| PHAC 2010 | Knowledge Mobilization | Develop plain-language approaches to convey complex scientific findings, processes, uncertainties, risks and shifts for various audiences/purposes, including:   - Health Portfolio staff, in areas such as policy, program, communications and operations decision makers/decision influencers (senior management and central agencies) - stakeholders (health professional associations, national aboriginal organizations, private sector, front-line health care workers, federal Healthcare Partnership, emergency response organizations, organizations representing at-risk populations, organized labour for the health sector, academic researchers and institutions) - the media - the public | |
| Sohrabi 2021 | Maintain new research roadmap | All facets of research (proposals, funding, basic research, clinical research, publication and translation into practice) have typically followed a slow and archaic roadmap | |
| Adam 2023 | Monitoring and Evaluation | Improve Data Collection and Monitoring  Systematic Data Collection on Health Research Indicators: Countries are encouraged to systematically collect data on key health research indicators to track progress and inform decision-making. This includes monitoring indicators related to official development assistance allocated to medical research and the share of gross domestic expenditures on health R&D. By collecting and analyzing this data, policymakers can assess the effectiveness of research investments and identify areas that require additional support  - Monitoring Progress and Identifying Gaps: By monitoring indicators related to health research funding and capacity, countries can track progress towards global health targets and identify disparities in resource allocation. This information is essential for guiding policy decisions, prioritizing research areas, and ensuring that investments are aligned with public health needs. Improving data collection and monitoring mechanisms can lead to more effective resource allocation and better preparedness for future health emergencies  Enhance Research Investments  Aligning Research Investments with Public Health Needs: The commentary highlights the importance of directing research investments towards areas that have a high burden of diseases, such as neglected tropical diseases and non-communicable diseases in low- and middle-income countries. By focusing funding on these priority areas, researchers can develop interventions and strategies that address the specific health needs of vulnerable populations  - Increasing Funding for Health Research in Low- and Middle-Income Countries: To improve preparedness for future pandemics, it is crucial to boost research funding in low- and middle-income countries. These regions often bear a disproportionate burden of diseases but receive a small share of global research investments. By increasing funding in these areas, researchers can develop solutions that are tailored to the local context and contribute to global health security | |
| Chen 2021 | Monitoring and Evaluation | Reviewing and revising some of the more long-standing indicators related to health research and development to encourage changes in decision-making strategies that would ensure progress towards the targets. | |
| Chen 2021 | Monitoring and Evaluation | Encouraging more countries to routinely report on proposed indicators related to health research and development to track progress and ensure progress towards global targets. | |
| Collins 2023 | Monitoring and Evaluation | Support Global Surveillance: The recommendation is to support local and global surveillance to achieve the earliest possible detection of emerging pathogens , . | |
| Devoe 2020 | Monitoring and Evaluation | To prepare for the next pandemic, the report suggests several recommendations based on the insights gained from studying the impact of COVID-19 on Community Health Centers:  Utilize Practice-Based Research Networks (PBRNs) for Real-Time Evaluation:  Practice-Based Research Networks (PBRNs) play a crucial role in conducting real-world, real-time evaluations to generate practice-based evidence during public health emergencies like the COVID-19 pandemic. PBRNs provide a unique infrastructure that allows for studying natural experiments and understanding the impact of such events on primary care practices. By leveraging the structure and expertise of PBRNs, researchers can quickly assess changes in care delivery, implementation strategies, and the effectiveness of interventions in response to a crisis. This real-time evaluation can provide valuable insights into what works in practice and inform evidence-based decision-making for future pandemics or public health emergencies.  Assess Multi-Level Factors:  When preparing for the next pandemic, it is essential to consider a wide range of multi-level factors that can influence the response of healthcare systems and primary care practices. These factors may include patient demographics (such as race, ethnicity, and insurance coverage), practice characteristics (location, size, patient volume), provider attributes (number of clinicians, specialties), healthcare utilization patterns, and policy changes (such as telehealth reimbursement policies).  Understanding how these factors interact and impact the delivery of care during a crisis can help in identifying disparities, barriers, and facilitators to effective response strategies. By assessing multi-level factors, healthcare systems can tailor interventions, training programs, and support mechanisms to address the specific needs of diverse populations and practice settings during future pandemics. | |
| Hanney et al., 2020 | Monitoring and Evaluation | Conduct context or situational analyses of current national position to inform strengthening activities | |
| Hanney et al., 2020 | Monitoring and Evaluation | Adopt monitoring and evaluation tools that focus on the objectives of the National Health Research System, including health system improvement | |
| Hinton et al (2021) | Monitoring and Evaluation | Use of theoretical frameworks: Research questions should be framed within theoretical frameworks to ensure relevance across sectors. Qualitative and quantitative methods are both necessary to study the unique contexts of MSCs, with theoretical generalizability being crucial.  Incorporation of sectoral analysis: Highly complex problems like sustainable development are better addressed through collaboration across diverse actors. Incorporating sectoral analysis into research methods is essential for understanding interactions within the collaborative network and identifying potential challenges and solutions.  Development of core research questions: A core set of research questions should be developed using mixed methods and contextual adaptations. Clear research quality criteria are needed to ensure rigor across disciplines and methods.  Identification of shared indicators of success and failure: Researchers should identify measures of progress relevant to all sectors involved in the MSC. This includes considering a broad spectrum of progress indicators and learning from both successful adaptations and challenges encountered during MSC implementation. | |
| Collins 2023 | Prioritization | Stabilize and Sustain Clinical Trial Networks: The recommendation is to stabilize and sustain large-scale global clinical trial networks to further enhance engagement with community hospitals and international partners, keeping these networks "warm" and ready for action when needed | |
| Demkowicz 2021 | Prioritization | The role of experts by experience should be prioritized, with co-production and patient and public involvement and engagement (PPIE) used to strengthen research in various ways | |
| Mishra 2023 | Prioritization | The establishment of more inclusive independent networks is highlighted as essential for addressing systemic issues and shaping tailored strategies for pandemic response. The article emphasizes the role of independent networks in embedding One Health perspectives, developing mitigation strategies for outbreaks among marginalized populations, and addressing how systemic racism and colonization shape epidemics and the response. It also stresses the importance of prioritizing and advocating for tailored strategies that address the specific needs of diverse communities | |
| Norton et al (2020) | Research Prioritization | Dynamic Nature of Pandemics: Pandemics, such as the COVID-19 outbreak, are dynamic and rapidly evolving situations. As new information emerges, research priorities may need to be adjusted to address emerging challenges, uncertainties, and gaps in knowledge.  Temporal Nature of Research Priorities: Understanding the temporal nature of research priorities is essential. Some research questions may be time-sensitive and can only be effectively addressed during specific phases of a pandemic, such as the transmission period. It is important to prioritize and conduct these studies promptly to generate timely and relevant evidence.  Urgency of Conducting Studies: There is a finite window of opportunity to answer critical research questions during a pandemic. Research efforts must be coordinated and expedited to maximize the impact of findings on public health interventions, treatment strategies, and preparedness measures.  Global Relevance and Contextual Considerations: Research priorities should be globally relevant while also taking into account the diverse contexts, needs, and resources of different regions and countries. Low and lower middle-income countries may face unique challenges that require tailored research priorities and interventions.  5Collaborative Approach: Engaging a diverse range of stakeholders, including researchers, policymakers, healthcare providers, and communities, in the process of assessing and updating research priorities can ensure that the most pressing issues are identified and addressed collaboratively.  Adaptability and Flexibility: The global health research system should be adaptable and flexible to respond to changing circumstances and emerging threats. Regular reviews and updates of research priorities can help align research efforts with the evolving landscape of pandemics. | |
| OECD 2023a | Prioritization | Align Funding with Global Health Priorities: The article highlights the significance of categorizing research activity and outputs in the health domain, particularly in identifying and prioritizing R&D projects related to public health emergencies. This recommendation suggests the importance of aligning R&D funding with global health priorities, such as those outlined by organizations like the World Health Organization (WHO), to ensure that funding allocation addresses pressing health challenges effectively. | |
| Office of the Chief Science Advisor of Canada | Prioritization | The report emphasizes the need for a deliberate effort to prioritize and fund research on data gaps during emergencies. Tight coordination and collaborations between the Chief Science Advisor and senior government officials are essential to ensure that funds are allocated in an agile manner and data are shared to support policy decisions | |
| Research in Low- and Middle-Income Countries Working Group, 2023 | Prioritization | - Define clear purpose to prevent misinterpretation of research agenda.  - Use transparent processes to instill confidence and trust in setting research priorities.  - Pre-position panels of experts before outbreaks for rapid identification of priorities.  - Employ systematic methodologies for developing research roadmaps.  - Ensure inclusive, transparent, and well-communicated processes for setting priorities.  - Incorporate feedback loops and plans for monitoring progress against research agendas. | |
| SRDC 2021 | Prioritization | The COVID-19 pandemic has highlighted the need for effective prioritization of research areas and allocation of resources to address the most critical needs. For example, vaccine development, treatment modalities, and public health interventions are all critical areas that require adequate attention and funding. Prioritizing these areas can help ensure that research efforts are focused on the most pressing needs and that resources are allocated effectively to address them.   Effective prioritization mechanisms can help ensure that research funding is directed towards areas that have the greatest potential to make a significant impact on the pandemic response. This can include mechanisms such as expert panels, advisory committees, and stakeholder consultations to identify research priorities and allocate resources accordingly.   By prioritizing research areas and allocating resources effectively, the research community can work towards developing effective interventions and strategies to address the COVID-19 pandemic. This can help to mitigate the impact of the pandemic on public health and the economy, and ultimately contribute to the global effort to control and eliminate the virus. | |
| Tsang et al., 2022 | Prioritization | Research regulatory body (like NIHR in the UK) that can set national research priorities and ensure clinical and research priorities are aligned. Additionally, coordination between major funding bodies (e.g., CIHR, NSERC) is needed for an integrated health system that algins health service delivery and research | |
| Kozlov 2022 | Reduce inequity | Investigate where and how any bias manifests, offer grants aimed at increasing faculty from under-represented groups, introduce bias training for peer reviewers, identify and address structural racism, support women academics who often have a heavier family -associated burden than their male counterparts. | |
| Hinton et al (2021) | Research Methods | The third recommendation that the authors propose is the use of various research methods in multisectoral collaboration, particularly mixed methods, to strengthen decision making abilities. In terms of the COVID-19 pandemic, the authors note that various restrictions could affect multisectoral collaborations leading to the use of novel approaches and methods, as well as the engagement of citizens. The authors also highlight the need for the methods to be adaptable to the changing pandemic situation. | |
| Camille 2021 | Resilience | Some lessons may be emerging from the pandemic on what types of contractual models or investment frameworks were able to withstand volatilities in markets, including demand, and also how systems, assets  and projects were able to manage risks or shocks. | |
| NHS 2021 | Resilience | The recommendation focuses on providing the research outputs that the NHS and wider health and social care system need to recover post-COVID-19, as well as to design and deliver services in a way that builds resilience. This includes a focus on greater integration of research within the NHS and wider health and social care services | |
| Ayano Ogawa 2019 | Resource Allocation & Capacity Building | Health research systems should prioritize the development and maintenance of core capacities related to prevention, detection, and response to infectious disease outbreaks. This includes investing in health workforce, laboratories, data systems, and risk communication. Improving response capacity is a key recommendation to improve health research system response to pandemics. This includes developing practical and tested hospital and inter-hospital level response plans for public health emergencies and mass casualty events and establishing systems above the hospital level that allow for coordinated management of beds and other finite resources including equipment and manpower. Improving response capacity is essential to ensure that health systems are prepared to respond effectively to pandemics and other public health emergencies. | |
| Becerra-Posada 2021 | Resource Allocation & Capacity Building | Given the recognized value of HPSR in decision-making and policy design, there may be a need for increased funding for HPSR to address the specific challenges and impacts of the COVID-19 pandemic on health systems and policies. | |
| Chen 2021 | Resource Allocation & Capacity Building | Encouraging funders of health research and development to make coordinated research prioritization decisions considering global and local public health needs and enabling informed decisions and coordinated efforts through increased data sharing of their funding activities. | |
| Collins 2023 | Resource Allocation & Capacity Building | The recommendation is to invest broadly in basic research in key disciplines such as virology, structural biology, genomics, molecular biology, immunology, and epidemiology to build fundamental knowledge and advance understanding of emerging pathogens. | |
| Rosenthal 2023 | Resource Allocation & Capacity Building | Jurisdictions considering a similar exercise emphasize the resources it will take in implementing this agenda before embarking on this process and set an agenda that is timely, realistic, and actionable. We utilized several steps that should increase the likelihood of our Research Agendas success, including pre-screening criteria, inclusion of feasibility of conducting the research and funding/ resources as 2 of our prioritization criteria, ensuring staff buy-in at multiple steps, and engaging program staff in the implementation of the agenda where practicable. | |
| Sohrabi 2021 | Resource Allocation & Capacity Building | Financial stimulation needs to be ensured, and whilst government-funded research bodies have  committed to upholding the funding of research, government spending in a time of recession is likely to have a toll on research funding. | The open sharing of knowledge and research efforts has stimulated global collaborative bonds with common purpose. It is our hope that these will continue beyond the pandemic, for the benefit of both education and research |
| SRDC 2021 | Resource Allocation & Capacity Building | During emergencies, there is often a need for rapid research to address urgent public health concerns. In these situations, strategic research may be prioritized to address the immediate needs of decision-makers. However, it is also important to continue to support open research, as it can lead to innovative solutions and new discoveries that may not have been identified through a strategic approach. | |
| Tsang et al., 2022 | Resource Allocation & Capacity Building | Research investment, including support for research ethics boards, contract review, research coordinators, establishing research policies and SOPs, databases, pharmacy and lab services. Note, these costs are not typically supported by provincial/territorial hospital budgets and funding agencies like CIHR do not provide funds to develop and maintain research infrastructure through the ebb and flow of studies. The CIHR Network of COVID-19 Clinical Trials Network is a step in this direction. The authors also suggest a model like the UK and the creation of the NIHR which provides dedicated research funding for hospitals. Hospitals in Canada could be incentivized to grow research programs by making research an accreditation standard through Accreditation Canada. | |
| WHO 2020 | Resource Allocation & Capacity Building | Address the brain drain by retaining trained scientists and building research capacity, particularly in low- and middle-income countries (LMIC). This involves providing training in research methodology, ethics, and priority-setting to enhance research capacity in LMICs and other regions | |
| Sohrabi 2021 | Safety | As research facilities begin to reopen, the safety of research participants and personnel is of paramount importance, and studies must be conducted in line with guidance such as the NIHR Restart Framework | Given current social distancing requirements and resource avail­ability, prioritisation of research, naturally favouring COVID-19-related work, is likely to continue for some time.  Institutions and research organisations should therefore work to ensure they provide appropriate physical space for work to be conducted. Space efficiency should be maximised by restructuring laboratories and establishing working partitions |
| Hanney et al., 2020 | Strategic planning | Develop a comprehensive and coherent National Health Research System strategy using 5 key policies: Five key policies are suggested for strengthening NHRSs:   - **Conduct Context Analyses**: Before implementing any strategies to strengthen NHRSs, it's essential to conduct context analyses. These analyses help identify the specific challenges, needs, and opportunities within a country's health research system. By understanding the local context, policymakers and research leaders can tailor their approaches to address the unique circumstances of their NHRSs. - **Develop Comprehensive Strategies**: NHRSs require comprehensive and coherent strategies to address the multifaceted challenges they face. These strategies should be outlined in policy documents or legislation to provide clear guidance and direction for strengthening the system. Comprehensive strategies encompass various components of NHRSs, including governance, financing, capacity-building, and research production. - **Engage Stakeholders**: The involvement of stakeholders is crucial in the development and implementation of NHRS strengthening strategies. Stakeholders may include policymakers, researchers, healthcare providers, patients, community representatives, and international partners. Engaging stakeholders ensures that the strategies are aligned with the needs and priorities of the healthcare system and the broader community. It also fosters ownership and commitment, leading to more effective implementation and sustainability of NHRS strengthening efforts. - **Adopt Monitoring and Evaluation Tools**: Effective monitoring and evaluation are essential for assessing the progress and impact of NHRS strengthening initiatives. Monitoring involves tracking the implementation of strategies, while evaluation focuses on assessing their outcomes and impacts. Adopting monitoring and evaluation tools allows policymakers and research leaders to identify areas of success, challenges, and areas for improvement. These tools should be aligned with the objectives of NHRSs, including improving health outcomes and promoting evidence-informed decision-making. - **Develop Partnerships**: Collaboration and partnerships are critical for strengthening NHRSs, especially in a globalized world where health challenges transcend national borders. Partnerships can be formed with international organizations, academic institutions, non-governmental organizations, and other stakeholders. These partnerships provide opportunities for resource mobilization, knowledge exchange, capacity-building, and collaborative research. By leveraging the expertise and resources of multiple partners, NHRSs can address complex health challenges more effectively. | |
| McMahon 2020 | Strategic Planning | The response emphasized the need for long-term planning to address the broader health system implications of the pandemic beyond immediate research needs. This includes considering the long-term consequences of the pandemic on health, health equity, and health system outcomes, as well as the post-COVID-19 health, social, and economic policies that are created. | |
| NIHR 2021 | Strategic Planning | The recommendation emphasizes the need to ensure that the entire research system is aligned and ready to develop, fund, and deliver pandemic research studies. This involves incentivizing greater use of platform studies to rapidly evaluate the effectiveness of multiple interventions to tackle new infectious diseases and supporting the work of the UK Health Security Agency through close partnership working | |
| OECD 2020 | Strategic Planning | Develop National Strategies: Governments and funding agencies should develop national strategies for research infrastructure management and operation. These strategies should align research infrastructure investments with national research priorities and ensure the efficient allocation of resources | |
| OECD 2023c | Strategic Planning | Promote Strategic Investment in Science and Innovation: The article underscores the critical role of science, technology, and innovation (STI) systems in responding to public health emergencies. This recommendation implies the need for sustained investment in R&D, scientific research, and innovation to enable proactive preparation and swift responses to future crises. Decision-makers may consider prioritizing strategic investment in STI systems to build resilience and enhance preparedness for global health challenges. | |
| Office of the Chief Science Advisor of Canada | Strategic Planning | The report recommends establishing effective science advisory mechanisms that are designed ahead of time, well understood by stakeholders, and can be quickly activated in an emergency. These mechanisms should include experts from academia, industry, and government and should be tailored to the nature of the emergency | The implication of this recommendation is that investing in research infrastructure, building research capacity, and fostering collaboration and coordination among stakeholders will enable the health research system to better respond to future pandemics. This will require sustained investment in research infrastructure and capacity building, as well as ongoing efforts to foster collaboration and coordination among stakeholders. |
| SRDC 2021 | Strategic Planning | The transformation of the health research ecosystem based on learnings from COVID-19 is crucial for ensuring its effective functioning in both emergency situations and on an ongoing basis. This transformation encompasses addressing challenges related to data sharing and integration, as well as inequities faced by women and BIPOC (Black, Indigenous, and People of Color) researchers.  Challenges for data sharing and integration have been highlighted during the COVID-19 pandemic, as the need for rapid and collaborative research has underscored the importance of efficient data sharing and integration across research initiatives. Transforming the health research ecosystem involves addressing these challenges by implementing improved data sharing protocols, promoting interoperability among research databases, and fostering a culture of collaboration and transparency in data sharing practices.  Inequities faced by women and BIPOC researchers have also been brought to the forefront during the pandemic, as these groups have experienced disproportionate challenges and barriers in accessing research opportunities and resources. Transforming the health research ecosystem involves addressing these inequities by implementing policies and initiatives that promote diversity, equity, and inclusion in research funding, career advancement, and representation within the research community. This can include measures such as targeted funding opportunities, mentorship programs, and initiatives to address systemic biases and barriers faced by underrepresented groups.  Overall, the transformation of the health research ecosystem based on learnings from COVID-19 is critical for ensuring its resilience and effectiveness in addressing public health challenges. This transformation involves addressing challenges related to data sharing and integration, as well as inequities faced by women and BIPOC researchers, to create a more inclusive, collaborative, and impactful research environment. | |
| Anderson 2022 | Structural components | "(a) organization and collaborations, (b) performance, (c) ethics and security, (d) scientific, (e) data, (f) information technology, and (g) patient outcomes." | |
| NHS 2021 | System Alignment | The recommendation emphasizes the need to prepare for future pandemics by ensuring that the whole research system is aligned and ready to develop, fund, and deliver pandemic research studies. This includes incentivizing greater use of platform studies to rapidly evaluate the effectiveness of multiple interventions to tackle new infectious diseases and supporting the work of the UK Health Security Agency through close partnership working | |
| Bouchard et al., 2023 | Training | Students and early career researchers and university administrators strongly emphasized the need for programs addressing the talent continuum to be simplified and harmonized to reduce burden on students, post-docs, early career researchers. | |

### APPENDIX F: Search Strings

**MEDLINE All (Ovid)**

| **#** | **Searches** | **Results** |
| --- | --- | --- |
| 1 | COVID-19/ | 247609 |
| 2 | exp COVID-19 Testing/ | 11918 |
| 3 | COVID-19 Vaccines/ | 22750 |
| 4 | SARS-CoV-2/ | 162313 |
| 5 | (nCoV or 2019nCoV or 19nCoV or COVID19 or COVID or SARS-COV-2 or SARSCOV-2 or SARS-COV2 or SARSCOV2 or SARS coronavirus 2 or Severe Acute Respiratory Syndrome Coronavirus 2 or Severe Acute Respiratory Syndrome Corona Virus 2).ti,ab,kf. | 382904 |
| 6 | or/1-5 | 393444 |
| 7 | limit 6 to yr="2020 -Current" | 393354 |
| 8 | ((research or evidence) and (response or infrastructure or fund or coordinat or generat or produc or conduct or consum or disseminat or use? or using or utiliz or utilis or mobiliz or mobilis)).ti. | 53554 |
| 9 | ((research or evidence) adj2 (response or infrastructure or fund or coordinat or generat or produc or conduct or consum or disseminat or use? or using or utiliz or utilis or mobiliz or mobilis)).ab. | 134671 |
| 10 | ((research or evidence) and (response or infrastructure or fund or coordinat or generat or produc or conduct or consum or disseminat or use? or using or utiliz or utilis or mobiliz or mobilis)).kf. | 8920 |
| 11 | Research Support as Topic/ | 22949 |
| 12 | or/8-11 | 208037 |
| 13 | (strength or strateg or success or achieve or accomplish or facilitat or enabl or effective or weakness or lesson? or learn or barrier? or challeng or opportunit or improve or obstacle? or difficult or constrain).ti,ab,kf. | 10551528 |
| 14 | 7 and 12 and 13 | 3708 |
| 15 | (Randomized Controlled Trial or Controlled Clinical Trial or Pragmatic Clinical Trial or Clinical Study or Adaptive Clinical Trial or Equivalence Trial).pt. | 701799 |
| 16 | (Clinical Trial or Clinical Trial, Phase I or Clinical Trial, Phase II or Clinical Trial, Phase III or Clinical Trial, Phase IV or Clinical Trial Protocol).pt. | 614411 |
| 17 | Multicenter Study.pt. | 340295 |
| 18 | Clinical Studies as Topic/ | 808 |
| 19 | exp Clinical Trial/ or exp Clinical Trials as Topic/ or Clinical Trial Protocol/ or Clinical Trial Protocols as Topic/ | 1290499 |
| 20 | Multicenter Study/ or Multicenter Studies as Topic/ or "Multicenter Study (topic)"/ | 360290 |
| 21 | Randomization/ | 107042 |
| 22 | Random Allocation/ | 107042 |
| 23 | Double-Blind Method/ | 176726 |
| 24 | Double Blind Procedure/ | 0 |
| 25 | Double-Blind Studies/ | 176726 |
| 26 | Single-Blind Method/ | 33081 |
| 27 | Single Blind Procedure/ | 0 |
| 28 | Single-Blind Studies/ | 33081 |
| 29 | Placebos/ | 35934 |
| 30 | Placebo/ | 0 |
| 31 | Control Groups/ | 2065 |
| 32 | Control Group/ | 2065 |
| 33 | Cross-Over Studies/ or Crossover Procedure/ | 55800 |
| 34 | (random or sham or placebo).ti,ab,kf. | 1631806 |
| 35 | ((singl or doubl) adj (blind or dumm or mask)).ti,ab,kf. | 199762 |
| 36 | ((tripl or trebl) adj (blind or dumm or mask)).ti,ab,kf. | 1708 |
| 37 | (control adj3 (study or studies or trial or group)).ti,ab,kf. | 1259266 |
| 38 | (clinical adj3 (study or studies or trial)).ti,ab,kf. | 800033 |
| 39 | (Nonrandom or non random or non-random or quasi-random or quasirandom).ti,ab,kf. | 55543 |
| 40 | (phase adj3 (study or studies or trial)).ti,ab,kf. | 142957 |
| 41 | ((crossover or cross-over) adj3 (study or studies or trial)).ti,ab,kf. | 53105 |
| 42 | ((multicent or multi-cent) adj3 (study or studies or trial)).ti,ab,kf. | 159052 |
| 43 | allocated.ti,ab. | 86233 |
| 44 | ((open label or open-label) adj5 (study or studies or trial)).ti,ab,kf. | 46219 |
| 45 | ((equivalence or superiority or non-inferiority or noninferiority) adj3 (study or studies or trial)).ti,ab,kf. | 12070 |
| 46 | (pragmatic study or pragmatic studies).ti,ab,kf. | 627 |
| 47 | ((pragmatic or practical) adj3 trial).ti,ab,kf. | 6296 |
| 48 | ((quasiexperimental or quasi-experimental) adj3 (study or studies or trial)).ti,ab,kf. | 12944 |
| 49 | trial.ti,kf. | 319849 |
| 50 | or/15-49 | 3709954 |
| 51 | 14 not 50 | 2970 |

**Embase (Elsevier)**

| **No.** | **Query** | **Results** |
| --- | --- | --- |
| 1 | 'coronavirus disease 2019'/de | 359654 |
| 2 | 'covid-19 testing'/exp | 10644 |
| 3 | 'sars-cov-2 vaccine'/de | 28772 |
| 4 | 'severe acute respiratory syndrome coronavirus 2'/de | 98172 |
| 5 | ncov:ti,ab,kw OR 2019ncov:ti,ab,kw OR 19ncov:ti,ab,kw OR covid19:ti,ab,kw OR covid:ti,ab,kw OR 'sars cov 2':ti,ab,kw OR 'sarscov 2':ti,ab,kw OR 'sars cov2':ti,ab,kw OR sarscov2:ti,ab,kw OR 'sars coronavirus 2':ti,ab,kw OR 'severe acute respiratory syndrome coronavirus 2':ti,ab,kw OR 'severe acute respiratory syndrome corona virus 2':ti,ab,kw | 430412 |
| 6 | #1 OR #2 OR #3 OR #4 OR #5 | 466292 |
| 7 | #6 AND [2020-2023]/py | 465999 |
| 8 | (research:ti OR evidence:ti) AND (response:ti OR infrastructure:ti OR fund:ti OR coordinat:ti OR generat:ti OR produc:ti OR conduct:ti OR consum:ti OR disseminat:ti OR use$:ti OR using:ti OR utiliz:ti OR utilis:ti OR mobiliz:ti OR mobilis:ti) | 61948 |
| 9 | ((research OR evidence) NEAR/2 (response OR infrastructure OR fund OR coordinat OR generat OR produc OR conduct OR consum OR disseminat OR use$ OR using OR utiliz OR utilis OR mobiliz OR mobilis)):ab | 186857 |
| 10 | (research:kw OR evidence:kw) AND (response:kw OR infrastructure:kw OR fund:kw OR coordinat:kw OR generat:kw OR produc:kw OR conduct:kw OR consum:kw OR disseminat:kw OR use$:kw OR using:kw OR utiliz:kw OR utilis:kw OR mobiliz:kw OR mobilis:kw) | 8628 |
| 11 | #8 OR #9 OR #10 | 204735 |
| 12 | strength:ti,ab,kw OR strateg:ti,ab,kw OR success:ti,ab,kw OR achieve:ti,ab,kw OR accomplish:ti,ab,kw OR facilitat:ti,ab,kw OR enabl:ti,ab,kw OR effective:ti,ab,kw OR weakness:ti,ab,kw OR lesson$:ti,ab,kw OR learn:ti,ab,kw OR barrier$:ti,ab,kw OR challeng:ti,ab,kw OR opportunit:ti,ab,kw OR improve:ti,ab,kw OR obstacle$:ti,ab,kw OR difficult:ti,ab,kw OR constrain:ti,ab,kw | 13483977 |
| 13 | #7 AND #11 AND #12 | 3250 |
| 14 | 'clinical study'/exp | 12435602 |
| 15 | 'clinical trial'/exp | 1868241 |
| 16 | 'clinical trial protocol'/de | 3324 |
| 17 | 'clinical trial (topic)'/exp | 449020 |
| 18 | 'randomization'/exp | 99169 |
| 19 | 'double blind procedure'/de | 212823 |
| 20 | 'single blind procedure'/de | 52454 |
| 21 | 'placebo'/de | 412114 |
| 22 | 'control group'/de | 109226 |
| 23 | 'crossover procedure'/de | 75760 |
| 24 | random:ti,ab,kw OR sham:ti,ab,kw OR placebo:ti,ab,kw | 2221924 |
| 25 | ((singl OR doubl) NEAR/1 (blind OR dumm OR mask)):ti,ab,kw | 284653 |
| 26 | ((tripl OR trebl) NEAR/1 (blind OR dumm OR mask)):ti,ab,kw | 2234 |
| 27 | (control NEAR/3 (study OR studies OR trial OR group)):ti,ab,kw | 1750995 |
| 28 | (clinical NEAR/3 (study OR studies OR trial)):ti,ab,kw | 1154700 |
| 29 | nonrandom:ti,ab,kw OR 'non random':ti,ab,kw OR 'quasi random':ti,ab,kw OR quasirandom:ti,ab,kw | 71150 |
| 30 | (phase NEAR/3 (study OR studies OR trial)):ti,ab,kw | 279857 |
| 31 | ((crossover OR 'cross over') NEAR/3 (study OR studies OR trial)):ti,ab,kw | 70229 |
| 32 | ((multicent OR 'multi cent') NEAR/3 (study OR studies OR trial)):ti,ab,kw | 261690 |
| 33 | allocated:ti,ab | 110956 |
| 34 | (('open label' OR 'open label') NEAR/5 (study OR studies OR trial)):ti,ab,kw | 87731 |
| 35 | ((equivalence OR superiority OR 'non inferiority' OR noninferiority) NEAR/3 (study OR studies OR trial)):ti,ab,kw | 17855 |
| 36 | 'pragmatic study':ti,ab,kw OR 'pragmatic studies':ti,ab,kw | 947 |
| 37 | ((pragmatic OR practical) NEAR/3 trial):ti,ab,kw | 8138 |
| 38 | ((quasiexperimental OR 'quasi experimental') NEAR/3 (study OR studies OR trial)):ti,ab,kw | 15825 |
| 39 | trial:ti,kw | 450706 |
| 40 | #14 OR #15 OR #16 OR #17 OR #18 OR #19 OR #20 OR #21 OR #22 OR #23 OR #24 OR #25 OR #26 OR #27 OR #28 OR #29 OR #30 OR #31 OR #32 OR #33 OR #34 OR #35 OR #36 OR #37 OR #38 OR #39 | 14620729 |
| 41 | #13 NOT #40 | 2290 |

**Google**

Search string 1:

intitle:(COVID19 | COVID) AND intitle:(research | evidence) AND intitle:(response | infrastructure | fund | generate | produce | consume | disseminate | use | using | utilize | utilise | mobilize | mobilise) filetype:pdf

Search string 2:

intitle:(COVID19 | COVID) AND intitle:(research | evidence) AND (strength | strategy | success | achieve | accomplish | facilitate | enable | effective | weakness | lesson | learn | barrier | challenge | opportunity | improve | obstacle | difficult | constrain) filetype:pdf

**Eligibility Criteria**

1. **Patient/Population:**
   - Publicly funded Health research organizations involved in Canada's health research system, as well as similar counterparts in other high income countries.
   - NOT: private or academic organizations holding their own research funding, or covid response
     - But do include lessons learned from these organizations that are funded by public and evaluate that
2. **Intervention:**
   - Conducting an environmental scan of both academic and grey literature that analyzes and assesses the response of the health research system to the COVID-19 pandemic (or previous public health emergency).
   - Does not have to speak to specific program (i.e., could be recommendations/future state)
3. **Outcome:**
   - Understanding the strengths, limitations, and lessons learned from the health research system response to COVID-19 in various countries.
   - Identify and describe organizational, programmatic, and governance structures and mechanisms necessary for an effective research response during health emergencies.
     - Not interested in detailing each organization but instead whether any of these worked or didn’t work well
   - Provide recommendations for an emergency-ready health research system in Canada to support effective decision-making and response during future health emergencies.
